# Novel NO-TZDs and trimethoxychalcone-based DHPMs: design, synthesis, and biological evaluation as potential VEGFR-2 inhibitors

**DOI:** 10.1080/14756366.2024.2358934

**Published:** 2024-06-21

**Authors:** Mater H. Mahnashi, Mohammed Nahari, Hassan Almasoudi, Abdulaziz Alhasaniah, Sara Elgazwi, Mahrous A. Abou-Salim

**Affiliations:** a Department of Pharmaceutical Chemistry, College of Pharmacy, Najran University, Najran, Saudi Arabia; b Department of Clinical Laboratory Sciences, College of Applied Medical Sciences, Najran University, Najran, Saudi Arabia; c Department of Chemistry, University of Derna, Derna, Libya; d Pharmaceutical Organic Chemistry, Faculty of Pharmacy, Al-Azhar University, Assiut, Egypt

**Keywords:** NO-TZD, 3,4,5-trimethoxychalcone, 1,4-dihydropyrimidine, OpenEye, five-dose

## Abstract

Novel series of nitric oxide-releasing thiazolidine-2,4-diones (**NO-TZD-3a-d,5,6**) and 3,4,5-trimethoxychalcone-based multifunctional 1,4-dihydropyrimidines (**CDHPM-10a-g**) have been designed and synthesised as potent broad-spectrum anticancer agents with potential VEGFR-2 inhibition. The designed analogs were evaluated for their anticancer activities towards a full panel of NCI-60 tumour cell lines and **CDHPM-10a-g** emerged mean %inhibitions ranging from 76.40 to 147.69%. Among them, **CDHPM-10e** and **CDHPM-10f** demonstrated the highest MGI% of 147.69 and 140.24%, respectively. Compounds **CDHPM-10a,b,d-f** showed higher mean %inhibitory activity than the reference drug sorafenib (MGI% = 105.46%). Superiorly, the hybrid **CDHPM-10e** displayed the highest potencies towards all the herein tested subpanels of nine types of cancer with MGI_50_ of 1.83 µM. Also, it revealed potent cytostatic single-digit micromolar activity towards the herein examined cancer cell lines. The designed compounds **CDHPM-10a-g** were exposed as potent non-selective broad-spectrum anticancer agents over all NCI subpanels with an SI range of 0.66–1.97. In addition, the target analog **CDHPM-10e** revealed potency towards VEGFR-2 kinase comparable to that of sorafenib with a sub-micromolar IC_50_ value of 0.11 µM. Also, **CDHPM-10e** could effectively induce Sub-G1-phase arrest and prompt apoptosis *via* caspase and p53-dependent mechanisms. Furthermore, **CDHPM-10e** revealed significant anti-metastatic activity as detected by wound healing assay. The modelling study implies that **CDHPM-10e** overlaid well with sorafenib and formed a strong H-bond in the DFG binding domain. The ADMET studies hinted out that **CDHPM-10e** met Pfizer’s drug-likeness criteria. The presented novel potent anticancer agent merits further devotion as a new lead product in developing more chalcone-based VEGFR-2 inhibitors.

## GRAPHICAL ABSTRACT

**Figure:**
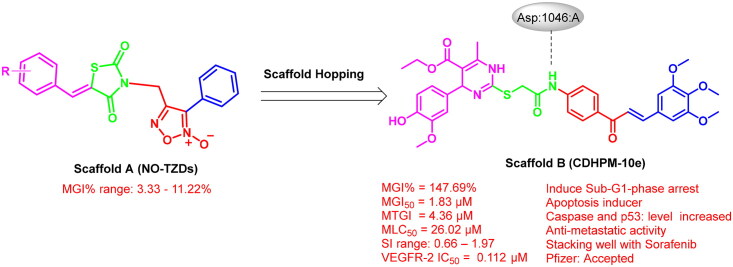

## Introduction

Cancer is an enormous burden for all worldwide. About 10 million people die annually, with more than $1.16 trillion cost^1–3^. Metastasis represents the major cause of death and is regulated mainly by angiogenesis, implying proliferation, metastasis, and endothelial differentiation^4^. Tumour aggressiveness and angiogenesis are strongly associated with KDR (Flk-1/VEGFR-2) kinase^5^. KDR has arisen increasing attention due to overexpression in many cancer types such as renal^6^^,^^7^, liver^6^^,^^7^, non-small cell lung (NSCLC)^6^^,^^8–10^, cervical^6^^,^^8^, breast^11^^,^^12^, colon^6^^,^^12^, and ovarian^13–15^ cancers. In addition, vascular endothelial growth factor VEGF is overexpressed in solid tumours, initiating the activation of KDR^16^ and hence triggering Raf/MEK/ERK downstream signalling cascade, leading ultimately too extreme acceleration of angiogenesis, tumour growth, and metastasis^7^^,^^16–18^. Sorafenib (SF) is a well-known potent KDR, c-Kit, PDGFR-ß, and VEGFR-3 kinases inhibitor targeting downstream MAPK cascade with potent activation of downstream apoptotic markers^19–21^. Interestingly, sorafenib and the next-generation anti-angiogenic drugs sunitinib, cabozantinib, regorafenib, nintedanib, and Lenvatinib have attracted increasing attention due to their activity towards a vast array of cancer types^22^. Regrettably, the major leading causes of concern are resistance, dose-limiting adverse effects, and impact on patient’s health, such as thrombotic effects, cardiotoxicity, and arterial hypertension^22^^,^^23^. Therefore, this study endeavours to develop a novel class of low-toxicity KDR TKIs and enrich the anti-angiogenic potential.

**Figure 1.**
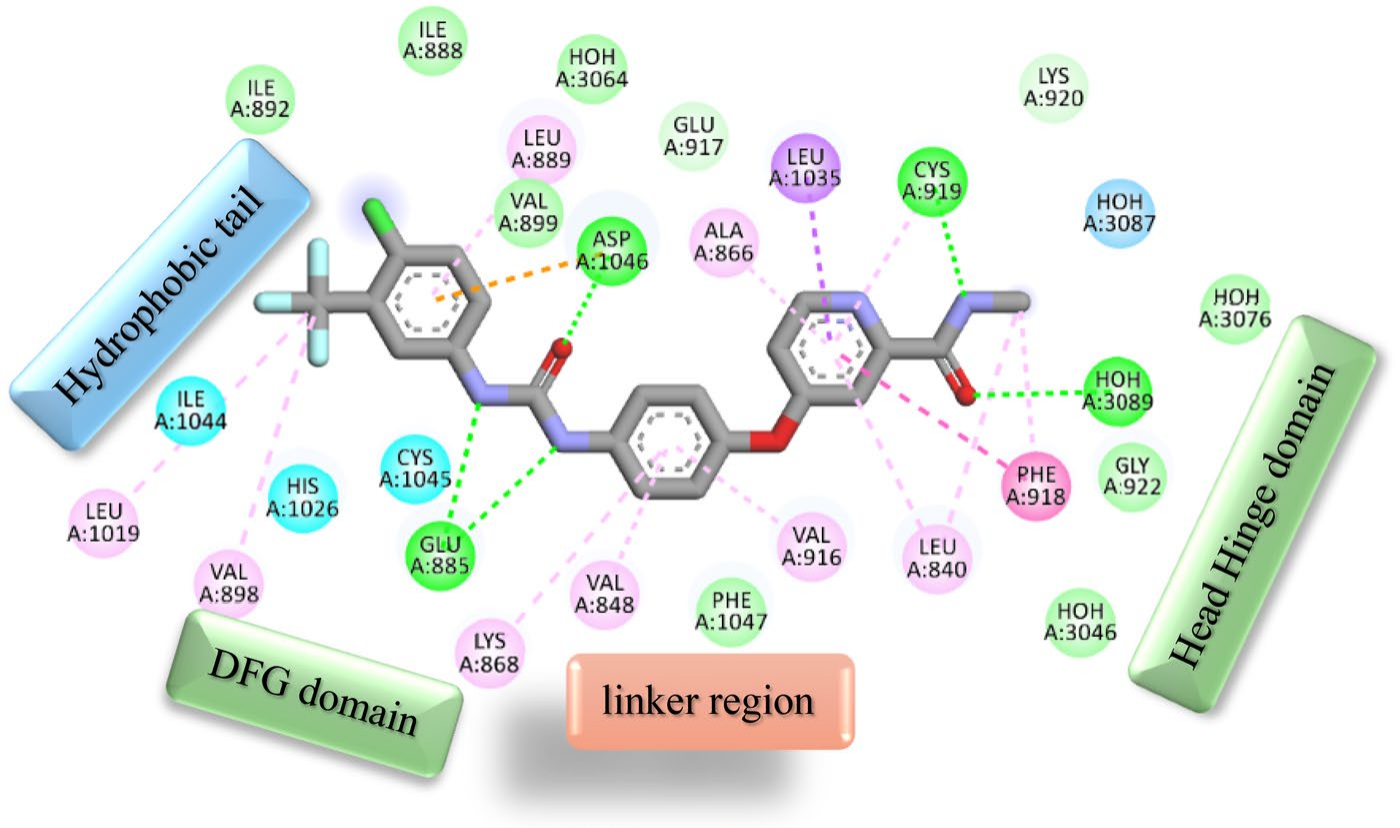
Basic pharmacophoric properties of KDR inhibitor sorafenib.

The straightforward pharmacophoric characteristics of sorafenib and the next-generation KDR inhibitors are summarised as follows. Firstly, the hydrophobic tail which occupies the allosteric binding site. Secondly, the H-bond acceptor and donor group interact with DFG domain residues (Glu:885 and Asp:1046). Thirdly, the central het/aryl moiety occupies the spacer domain. Fourthly, the heterocyclic scaffold interacts with the ATB binding domain, as seen in Figure 1. Therefore, developing a novel generation with these pharmacophoric characteristics has increased attention.

**Figure 2.**
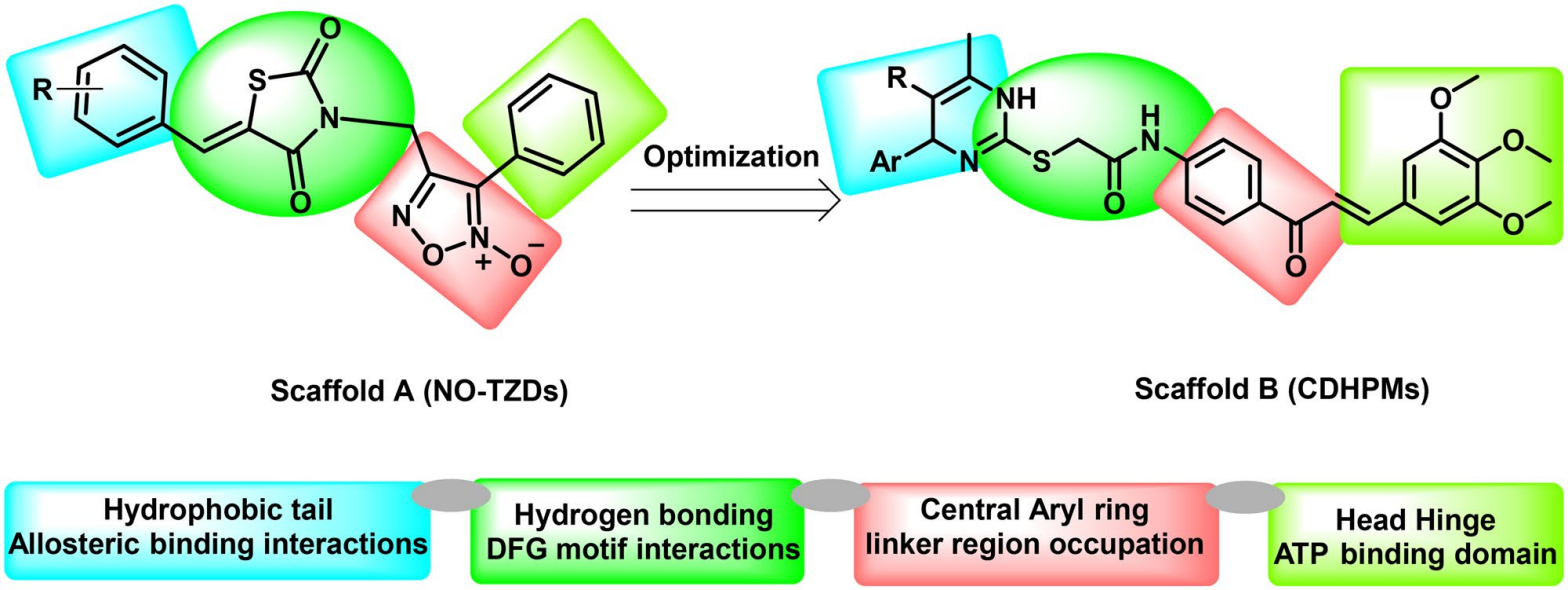
Scaffold hopping of target compounds NO-TZDs and CDHPMs.

Based on the electrostatic potential similarities and physically realistic shapes utilising OpenEye’s EON module^24^^,^^25^, scaffolds “A” and “B” (Figure 2), have been identified as promising motifs for designing a novel generation of angiogenesis inhibitors with enhanced bioavailability and improved biological activity^20^. The pharmacophoric properties of scaffold “A” are characterised by furoxan moiety which occupies the linker region, the TZD group which inserts into the DFG motif, the aryl moiety which interacts with the hydrophobic region and the terminal phenyl group which occupies head hinge region. Interestingly, the chemical fragments, furoxan, and TZD, have been confounded in numerous hybrids as potential anticancer agents^20^^,^^26^. Our group has recently conferred that furoxan-based pyrazolo[3,4-d]pyrimidines showed potential anti-VEGFR-2 activity^20^. In addition, Taghour et al.^27^ and Upadhyay et al.^28^ have unveiled some TZDs hybrids as potential VEGFR-2 inhibitors. Therefore, scaffold “A” could serve as a promising target for angiogenesis inhibition.

On the other hand, EON scaffold hopping offered scaffold “B” which is rationalised as follows. Firstly, the incorporation of 3,4,5-trimethoxyphenyledine base scaffold to fit tightly in the ATP-binding region. Secondly, the introduction of a phenyl ketone group to occupy the linker region. Thirdly, the open *S*-acetamide bridge allows hydrogen bonding formation with the DFG domain. Fourthly, endowing with 1,4-dihydropyrimidine scaffold as a hydrophobic tail to allow deep insertion into the allosteric active site. Finally, steric, and electronic effects, hydrophilicity, and hydrophobicity were also explored.

It is worth stressing that the isosteric 1,4-dihydropyrimidine scaffold has been reported in numerous anti-tumour agents^29^^,^^30^. Particularly, monastrol is a well-known anticancer drug with a 1,4-dihydropyrimidine-2-thione core^31–33^. Also, Marzouk et al. described a potent KDR inhibitor (compound I) with a 1,6-dihydropyrimidine-2-thio scaffold^33^. Therefore, the privileged 1,4-dihydropyrimidine core has been deemed a promising motif for discovering a novel VEGFR-2 inhibitor generation.

Furthermore, the 3,4,5-trimethoxychalcone motif has been exposed to be beneficial for antitumor action in earlier SAR studies^34–37^. Remarkably, this flavonoid class plays a pivotal role in exhibiting promising biological effects towards tumour hallmarks such as proliferation^4^, stemness^4^, inflammation^3^^,^^4^^,^^37^ angiogenesis^4^^,^^37^, metastasis^4^, invasion^4^, regulation of tumour epigenetics^35^^,^^36^, cell cycle arrest and apoptosis^37^, inhibition of multidrug resistance proteins^37^^,^^38^ as well as potent inhibitory activity towards VEGFR-2^39^. Interestingly, besides methoxylated chalcones, other marine symmetric bromophenol natural products derived from macroalgae displayed potent anticancer activities^40^. Of particular interest, a chalcone bromophenol analog BDDE with a three-atom bridging two phenyl groups has drawn considerable attention due to its potential antitumor activity. BDDE displayed broad-spectrum *in vitro* anticancer activities with potent induction of apoptosis through mitochondrial cascade^40^. BDDE has also been found to inhibit VEGF, VEGFR, and topoisomerase I, thus potentially repressing angiogenesis^41^. Therefore, the 3,4,5-trimethoxychalcone scaffold spaced with enone moiety in the three-atom linker may significantly impact tumour growth and metastasis^33^.

Enlightened by those above and in continuation to our efforts^20^, we have been inspired to develop furoxan-based thiazolidine-2,4-diones and trimethoxychalcone-based dihydopyrimidines as a novel generation of VEGFR-2 TKIs based on isosterism and combination principles.

## Results and discussion

### Chemistry

**Scheme 1.**
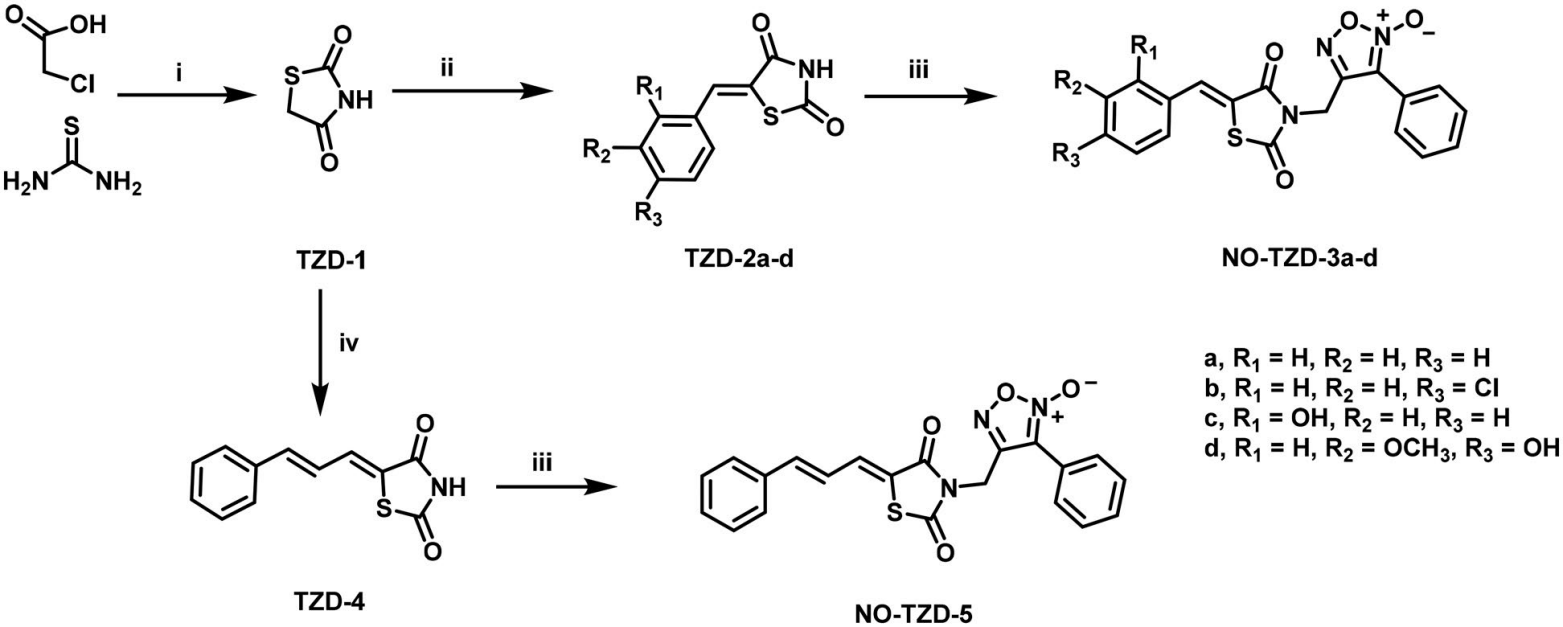
Synthesis of target furoxan-based thiazolidine-2,4-diones **NO-TZD-3a-d,5**. (i) HCl 33%, reflux; (ii) Araldehyde, piperazine (0.8 eq.), EtOH, 1–2 h, reflux; (iii) Furoxan mesylate, K_2_CO_3_, DMF, 65 °C, 1 h; (iv) cinnamaldehyde, piperazine (0.8 eq.), EtOH, 2 h, reflux.

**Scheme 2.**
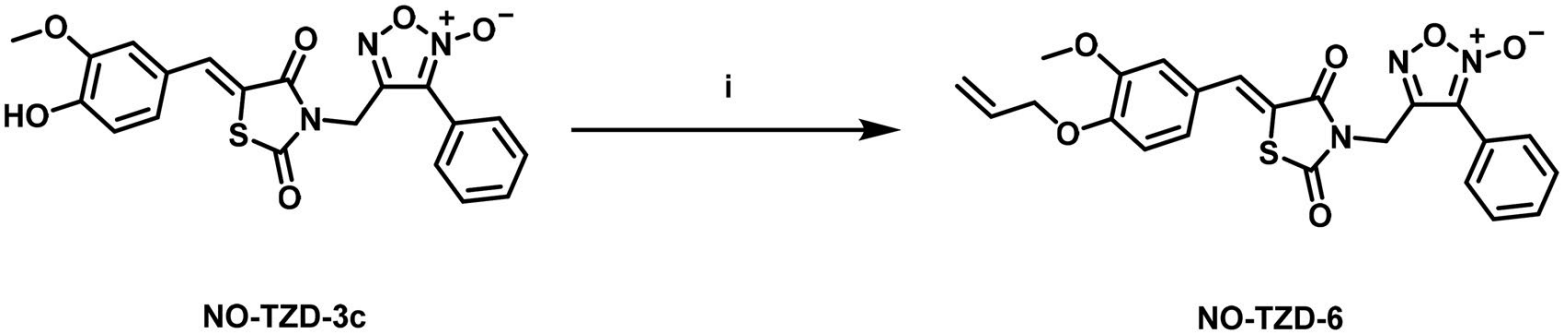
Synthesis of allyl derivative **NO-TZD-6**. (i) allyl bromide, K_2_CO_3_, DMF, 80 °C, 1 h.

Scheme 1 demonstrates the synthetic pathway of target compounds **NO-TZD-3a-d** and **NO-TZD-5**. Condensation of **TZD-1** with different aldehydes in absolute ethanol containing 0.8 equivalent of piperazine^42–44^ provided the key intermediates TZDs **2a-d** and **4**^43^^,^^45^^,^^46^ which underwent *N*-alkylation with furoxan mesylate in DMF containing potassium carbonate at 65 °C to yield **NO-TZD-3a-d** and **NO-TZD-5**. On the other hand, *O*-allylation of **NO-TZD-3c** with allyl bromide in DMF containing potassium carbonate at 80 °C afforded the allyl derivative **NO-TZD-6** (Scheme 2). Since the chemical shift of the exocyclic olefinic proton in the (*Z*)-isomer is relatively deshielded rather than the (*E*)-one, the *Z*-configuration of TZDs was verified by comparing with the incremented chemical shifts^43^^,^^45–47^. Hence, the existence of singlet signal corresponding to the vinylic proton at 7.93–8.09 ppm demonstrates the *Z*-configuration of TZDs.

**Scheme 3.**
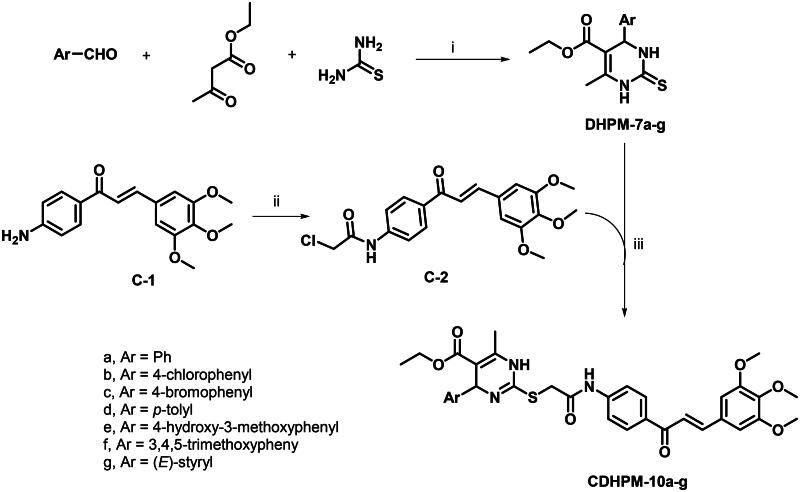
Synthetic route of target compounds **CDHPM-10a-g**. (i) EtOH/HCl, reflux, 6 h; (ii) chloroacetyl chloride, TEA, ACN, RT, 1 h; (iii) K_2_CO_3_, NaI, DMF, RT, 2 h.

The 3,4,5-trimethoxychalcone-based 1,4-dihydropyrimidine-2-thio analogs **CDHPM-10a-g** were prepared as depicted in Scheme 3. The reaction route started with monastrol analog precursors (**DHPM-7a-g**) which were prepared according to Biginelli’s one-pot reaction of araldehyde, ethyl acetoacetate, and thiourea in 0.13 M ethanolic HCl^48–53^. On the other hand, the condensation of *p*-aminoacetophenone with 3,4,5-trimethoxybenzaldehyde upon stirring with potassium hydroxide in absolute ethanol afforded 3,4,5-trimethoxychalconamine **C-1**^54^^,^^55^, which underwent acetylation with chloroacetyl chloride to yield the chalcone chloride intermediate **C-2**^56–58^. The target compounds 3,4,5-trimethoxychalcone-based 1,4-Dihydropyrimidine-2-thio analogs (**CDHPM-10a-g**) were then synthesised through *S*-alkylation of monastrol analogs and chalcone chloride **C-2** in presence of K_2_CO_3_ and catalytic amount of sodium iodide. Regarding the precursor **DHPM-7a** (Figure S13, Supplementary Material), the *R*-configuration around C4 was assigned by ^1^H NMR signal at about 5.19 ppm corresponding to pyrimidine C4-H upon comparing to the *R*-isomers reported by Alvim et al.^59^ at 5.10–514 ppm. In addition, the ^1^H NMR of methyl ester analog of DHPM-7a (Figure S14, Supplementary Material) characterised the C4-H at 5.19 ppm which augments the *R*-configuration assignments. On the other hand, Wang et al.^50^ reported the *S*-isomer which characterised by down field shifting of C4-H at 5.41 ppm. Moreover, the *E*-configuration of CDHPMs was verified by the doublet signals of CHs with *J*-coupling ≈ 15.4 Hz corresponding to the exocyclic olefinic proton. Spectra are given in Figures S1–S29 (see Supplementary Material) and have been entirely consistent with the synthesised analogs.

### Biological evaluation

#### NCI-USA anticancer activities

The herein designed conjugates **NO-TZD-3a-d,5,6** (NSCs: 838 404, 844 385, 844 376, 838 406, 838 405, and 845 060, respectively) and **CDHPM-10a-g** (NSCs: 842 638, 842 636, 842 637, 842 635, 842 634, 842 639, and 842 640, respectively) have been screened at NCI-DTP (MD, USA) for their *in vitro* antitumor activities towards a full panel of nine types of cancers comprising leukaemia, CNS, colon, ovarian, breast, melanoma, prostate, renal and NSCLC cancers using SRB assay following their standard protocol^60–62^. The herein designed analogs were examined firstly at 10 µM (single-dose screen) and then at 0.01–100 µM (five-dose screen) for those that demonstrated high potency at 10 µM concentration.

##### Preliminary NCI-USA one-dose screen

**Figure 3.**
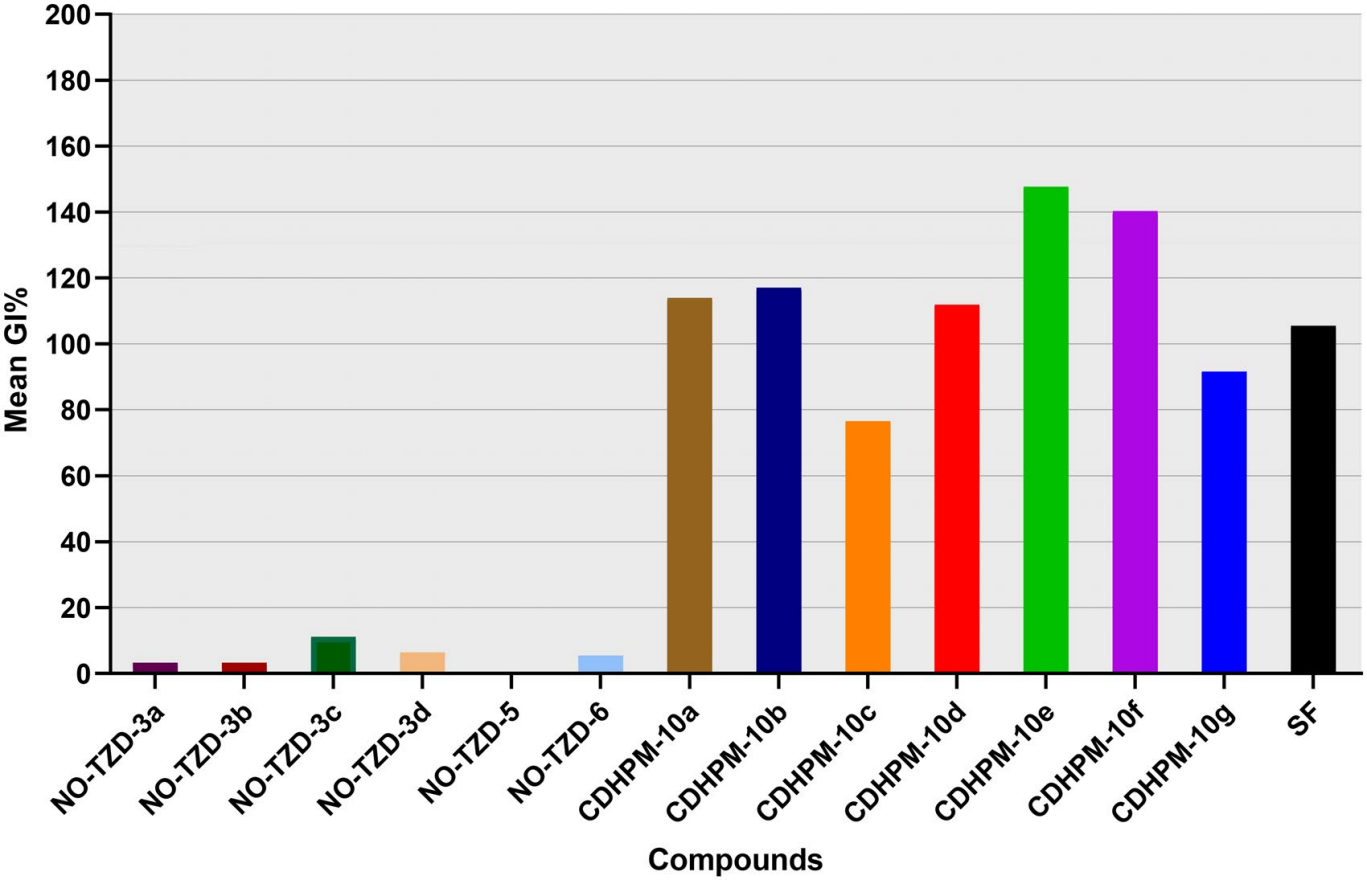
Mean %inhibitions of the designed analogs **NO-TZD-3a-d,5,6, CDHPM-10a-g** and reference drug sorafenib (SF) at 10 µM over the herein examined NCI cancer cells.

The accomplished results (Figure 3) unveiled that the target compounds **NO-TZD-3a-d** exhibited very low activity towards the full NCI cell panel with mean growth inhibition percentage (MGI%) ranging from 3.33 to 11.22%. Also, the modified hydrophobic tail targets **NO-TZD-5** and **NO-TZD-6** did not improve the overall activity displaying an MGI% of 5.49%. On the other hand, the herein examined 3,4,5-trimethoxychalcone-based multifunctional 1,4-dihydropyrimidines, **CDHPM-10a-g,** demonstrated high potency with significant MGI% spanning in the range: 76.40–147.69%. Superiorly, **CDHPM-10e**, and **CDHPM-10f** exhibited mean %inhibitions of 147.69 and 140.24%, respectively, whereas compounds **CDHPM-10a**, **CDHPM-10b**, and **CDHPM-10d** displayed MGI% of 113.88, 116.97, and 111.87%, respectively. Compared to the reference drug sorafenib (MGI% = 105.46%), compounds **CDHPM-10a**, **CDHPM-10b,** and **CDHPM-10d-f** were more potent. Moreover, the hybrids **CDHPM-10c** and **CDHPM-10g** exhibited significant MGI% of 76.40 and 91.51%, respectively, but lower than sorafenib. In addition, the maximum %inhibition achieved against the Leukaemia subpanel was 100%, while the other types of cancer were more. It is worth underlining that all the herein designed **CDHPM-10a-g** analogs demonstrated variable lethal effects (>100% growth inhibition) at a 10 µM screen. Compounds **CDHPM-10a-g** were lethal over 31, 33, 9, 29, 46, 44, and 15 cells, respectively. The influence of substitution at 1,4-dihydropyrimidine C4 was also studied. The results hinted out that the 4-hydroxy-3-methoxy derivative **CDHPM-10e** was more active than the corresponding 3,4,5-trimethoxy derivative **CDHPM-10f** as the *m-*methoxy group of the head phenyl may form an additional hydrogen bond inside the receptor active pocket leading to optimal binding interaction, and therefore higher anticancer action. Furthermore, the chloro derivative **CDHPM-10b** showed higher activities than the corresponding unsubstituted analog **CDHPM-10a,** which is more active than the tolyl analog **CDHPM-10d**. This may be ascribed to the influence of hydrophobic interactions within the receptor’s active site. Moreover, the styryl hybrid **CDHPM-10g** exerted a significant inhibitory activity than the bromo derivative **CDHPM-10c** since the styryl moiety may geometrically enable tight fitting in the receptor’s active site. Therefore, it can be inferred that the steric and electronic effect(s) of hydrophilic motifs at C4 of the privileged 1,4-dihydropyrimidine scaffold could impact the affinities towards the receptor’s active site. Interestingly, the 3,4,5-trimethoxyphenyledine head base scaffold could help to fit tightly in the head hinge ATP-binding domain, the phenyl ketone moiety forms hydrophobic interactions with the linker region, the open S-acetamide bridge allows hydrogen bonding formation with the DFG motif and finally endowing with multifunctional 1,4-dihydropyrimidine scaffold could deserve deep insertion into the allosteric active site of the herein target enzyme. The herein reported analogs, **CDHPM-10a-g**, displayed high mean %inhibitions with broad-spectrum anticancer activity over the NCI-tested panel (Table 1); thus, they were selected for five-dose testing.

**Table 1.** Percentage inhibitions of hybrids **CDHPM-10a-g** and sorafenib (SF) towards the herein tested NCI tumour cells.

Panel	Cell Line	CDHPM-							SF
		10a	10b	10c	10d	10e	10f	10g	
Leukaemia	CCRF-CEM	84.56	77.44	83.74	79.66	93.98	81.72	78.52	81.4
	HL-60(TB)	81.72	84.69	81.39	81.24	93.99	85.06	84.08	76.7
	K-562	88.15	86.31	81.07	86.34	89.30	88.32	86.95	70.8
	MOLT-4	75.56	74.53	69.60	74.52	84.77	83.03	82.68	74.2
	RPMI-8226	95.97	94.50	94.74	95.02	100.23	97.18	96.67	119.3
	SR	92.27	91.98	90.64	94.01	91.03	92.44	92.68	76.1
NSCLC	A549/ATCC	65.42	76.08	35.51	70.93	99.98	96.58	42.10	136.4
	EKVX	117.00	121.33	71.63	96.69	165.27	159.69	76.43	102.9
	HOP-62	86.44	87.44	38.41	81.65	134.88	115.87	61.25	77.5
	NCI-H226	125.13	130.08	80.70	130.25	152.83	160.26	98.46	123.1
	NCI-H23	155.80	156.87	72.17	140.82	166.88	174.44	88.78	119.9
	NCI-H322M	48.63	53.29	25.34	49.67	149.03	83.84	24.00	81.7
	NCI-H460	125.15	91.04	67.64	91.94	145.88	153.99	77.97	97.4
	NCI-H522	125.10	149.67	57.31	145.39	165.45	132.53	89.32	138.9
Colon cancer	COLO 205	81.26	92.26	12.84	85.25	166.44	154.26	30.91	124.7
	HCC-2998	194.52	195.72	177.73	191.96	195.79	197.20	192.95	NT
	HCT-116	165.42	176.49	168.64	171.11	159.82	160.82	162.65	90.8
	HCT-15	170.24	174.96	169.80	169.83	173.37	163.89	169.91	94.5
	HT29	98.53	99.43	93.98	110.44	123.05	97.03	93.12	90.2
	KM12	168.45	166.46	95.54	168.48	180.67	186.03	118.62	114.7
	SW-620	136.09	108.16	92.51	99.58	156.30	146.23	100.98	94.6
CNS cancer	SF-268	96.65	80.54	60.75	65.65	109.35	116.52	65.66	92.7
	SF-295	91.78	82.20	40.10	83.98	169.36	166.73	63.21	93.9
	SF-539	185.02	189.33	125.67	191.85	195.07	191.02	182.28	89.2
	SNB-19	118.25	106.92	51.37	100.40	145.50	102.40	63.85	79.4
	SNB-75	66.17	31.45	51.54	49.00	90.30	161.36	81.98	78.2
	U251	162.23	167.78	85.18	141.90	155.63	170.36	146.50	99.9
Melanoma	LOX IMVI	188.15	191.60	188.17	185.87	194.05	195.82	194.48	155.1
	MALME-3M	104.85	115.55	42.96	106.10	163.08	161.34	60.62	47
	M14	128.26	140.34	71.96	143.37	169.03	153.53	94.42	94.7
	MDA-MB-435	141.05	114.68	96.77	127.26	156.17	136.67	122.86	135.2
	SK-MEL-2	125.74	149.39	80.83	134.48	144.48	138.56	100.37	142.9
	SK-MEL-28	138.09	164.66	101.21	158.01	184.01	164.46	146.57	126.7
	SK-MEL-5	147.66	159.48	64.19	146.36	195.64	195.43	72.41	156.6
	UACC-257	91.08	127.20	40.42	104.91	143.43	134.23	49.02	NT
	UACC-62	140.48	145.58	71.31	133.45	191.60	156.28	78.11	125.5
Ovarian cancer	IGROV1	138.45	124.89	70.15	123.88	151.87	148.95	83.44	170.7
	OVCAR-3	163.18	141.28	134.77	165.99	184.92	161.32	152.63	96.8
	OVCAR-4	63.15	52.86	39.17	50.00	77.63	98.87	56.58	74.8
	OVCAR-5	120.81	144.66	10.60	143.26	174.99	154.93	34.04	84.6
	OVCAR-8	96.65	99.11	92.72	99.32	111.94	110.70	95.28	116.5
	NCI/ADR-RES	114.67	122.26	92.15	115.51	120.73	134.94	87.17	112.5
	SK-OV-3	22.17	16.10	−2.61	27.99	154.92	152.99	14.03	87.5
Renal cancer	786-0	158.61	127.94	68.19	97.97	186.71	188.32	84.98	84.5
	A498	24.82	30.27	−30.76	31.38	162.70	142.67	3.92	121.1
	ACHN	93.63	97.65	69.44	93.22	182.77	181.65	90.24	100
	CAKI-1	81.49	90.88	57.09	71.66	177.45	169.87	69.77	132.6
	RXF 393	190.02	186.38	166.90	186.42	193.37	194.80	184.40	156.3
	SN12C	86.19	97.41	61.26	78.48	147.18	121.20	68.84	107.2
	TK-10	67.15	94.99	15.41	77.79	94.16	91.60	38.06	74.2
	UO-31	174.83	170.88	65.84	176.73	199.50	196.71	89.01	181.1
Prostate cancer	PC-3	73.86	73.51	67.67	72.47	97.06	83.66	73.30	109.7
	DU-145	148.74	159.15	84.66	135.94	192.17	153.49	93.60	86.3
Breast cancer	MCF7	96.78	127.55	94.72	99.92	178.91	154.61	94.38	96.3
	MDA-MB-231/ATCC	107.83	174.85	123.08	168.55	139.73	100.33	167.11	134.5
	HS 578 T	60.91	53.74	59.92	54.89	68.73	79.01	70.37	108.1
	BT-549	132.17	138.83	95.92	144.31	185.34	161.20	103.08	75.6
	T-47D	82.07	103.50	61.70	85.21	87.39	96.87	81.99	92.5

–: no growth inhibition over the course of the experiment; 0–100: growth inhibition %; >100% growth inhibition: the tested compounds demonstrated variable lethal effects; NT: not tested.

##### In vitro five-dose screening

The promising designed hybrids, **CDHPM-10a-g**, were further tested to determine their 50% cell growth inhibition (GI_50_), total cell growth inhibition (TGI), and median lethal concentration (LC_50_) against the herein examined nine cancer types using a five-dose assay according to NCI standard protocol; the data are presented in Table 2 and Tables S1, S2 (see Supplementary Material).

**Table 2.** GI_50_ (µM) of target analogs **CDHPM-10a-g** and sorafenib (SF).

Panel	Cell line	CDHPM-							SF
		10a	10b	10c	10d	10e	10f	10g	
Leukaemia	CCRF-CEM	2.42	2.25	2.22	1.94	1.88	2.17	2.72	1.99
	HL-60(TB)	1.67	2.28	2.93	2.32	2.1	2.22	1.55	1.58
	K-562	2.36	2.85	4.17	3.35	2.61	3.05	3.24	3.16
	MOLT-4	2.33	2.91	2.29	2.58	2.39	1.91	2.46	3.16
	RPMI-8226	2.07	1.17	2.11	1.75	1.41	2.05	1.73	1.58
	SR	1.85	1.97	2.74	2.44	1.72	2.18	1.77	3.16
NSCLC	A549/ATCC	3.14	3.73	10.22	6.47	2.38	3.08	9.36	3.16
	EKVX	2.6	2.98	10.19	4.8	1.97	2.08	7.11	2.51
	HOP-62	2.48	3.17	6.88	3.96	1.45	2.29	5.12	1.99
	HOP-92	2.34	2.22	3.41	3.3	2.14	2.88	3.5	1.58
	NCI-H226	2.09	2.22	8.89	3.19	1.81	1.73	5.97	1.99
	NCI-H23	2.27	2.42	10.08	4.48	1.7	1.94	6.88	1.99
	NCI-H322M	3.86	3.6	10.7	7.91	1.79	3.71	6.21	2.51
	NCI-H460	2.55	3.1	6.93	4.33	1.82	1.93	5.2	2.51
	NCI-H522	1.56	1.99	3.23	1.76	1.41	1.68	2.31	1.99
Colon cancer	COLO 205	3.72	3.41	10.79	7.67	1.89	2.26	10.21	1.99
	HCC-2998	1.74	1.71	3.38	1.92	1.7	1.68	2.17	3.16
	HCT-116	2.58	1.51	1.73	1.79	1.62	1.42	1.71	1.58
	HCT-15	1.63	1.57	1.7	1.64	1.55	1.83	1.79	2.51
	HT29	1.83	2.01	2.26	1.86	1.83	1.91	1.99	1.99
	KM12	1.7	1.87	3.52	2.19	1.38	1.65	2.63	1.58
	SW-620	2	2.05	2.46	2.22	1.7	2	2.14	2.51
CNS cancer	SF-268	2.61	2.69	4.18	4.47	1.76	2.22	3.1	2.51
	SF-295	2.94	3.2	5.53	3.93	1.79	1.68	4.81	1.58
	SF-539	1.67	1.71	3.23	1.88	1.53	1.65	1.83	1.58
	SNB-19	2.87	2.9	6.53	3.26	2.54	2.22	4.7	3.16
	SNB-75	1.54	ND	0.83	1.38	1.38	1.17	1.49	3.16
	U251	1.63	1.69	3.48	1.72	1.65	1.7	2.32	1.99
Melanoma	LOX IMVI	1.71	1.64	2.22	1.74	1.46	1.68	1.84	1.58
	MALME-3M	1.86	1.63	10.13	2.56	1.41	2.01	4.41	1.99
	M14	2.6	2.28	7.17	3.36	1.98	1.93	3.3	1.99
	MDA-MB-435	1.77	1.73	2.44	1.83	1.95	1.89	1.86	1.58
	SK-MEL-2	2.41	2.08	10.03	3.82	1.8	2.13	3.11	1.99
	SK-MEL-28	2.03	1.89	3.93	2.11	1.93	1.87	2.31	2.51
	SK-MEL-5	1.83	2.16	4.16	3.56	1.63	1.66	4.46	1.58
	UACC-257	2.19	2.33	9.24	4.66	1.72	2.23	5.29	1.99
	UACC-62	2.26	2.3	4.73	2.44	1.56	1.7	4.03	1.58
Ovarian cancer	IGROV1	1.66	1.66	2.21	2.03	1.55	1.87	1.67	2.51
	OVCAR-3	1.71	1.77	3.5	1.93	1.65	1.93	2.19	3.16
	OVCAR-4	3.26	3.54	7.65	4.69	2.7	2.49	5.36	3.16
	OVCAR-5	2.16	1.94	10.17	3.09	1.79	1.84	5.18	3.16
	OVCAR-8	1.81	2	3.66	3.3	2.11	1.92	3.17	3.16
	NCI/ADR-RES	2.37	2.13	3.48	2.76	2.08	2.45	3.47	2.51
	SK-OV-3	6.18	8.59	20.1	10.17	1.59	2.08	10.88	2.51
Renal cancer	786-0	2.5	2.34	7.52	4.14	1.95	1.86	4.32	3.16
	A498	10.41	10.27	10.78	10.59	2.55	10.62	10.49	2.51
	ACHN	2.19	1.89	10.02	2.15	1.69	1.79	3.45	2.51
	CAKI-1	1.67	1.84	3.82	3.39	1.64	2.02	3.7	3.16
	RXF 393	1.81	1.36	1.48	1.55	1.17	1.43	1.64	2.51
	SN12C	2.19	3.13	4.3	2.96	1.67	1.67	3.36	2.51
	TK-10	4.09	3.92	20.09	10.25	2.34	2.63	10.05	3.98
	UO-31	1.48	1.54	3.1	1.7	1.32	1.61	1.68	2.51
Prostate cancer	PC-3	3.13	2.85	3.38	3.63	2.09	2.43	3.16	1.99
	DU-145	3.25	1.95	9.33	3.47	1.63	3.24	5.62	3.16
Breast cancer	MCF7	2.68	1.75	2.96	3.1	1.6	2.49	3.07	2.51
	MDA-MB-231/ATCC	1.95	1.62	2.86	2.01	1.83	2.27	2.13	1.26
	HS 578T	ND	ND	ND	ND	2.41	3.61	3.01	2.51
	BT-549	2.45	1.53	2.38	2.08	1.61	1.37	3.05	3.16
	T-47D	2.65	2.32	4.85	3.56	2.21	2.59	3.34	1.58
	MDA-MB-468	1.79	1.77	2.58	2.11	1.67	1.65	2.36	1.99

ND: not determined.

###### GI_50_

**Figure 4.**
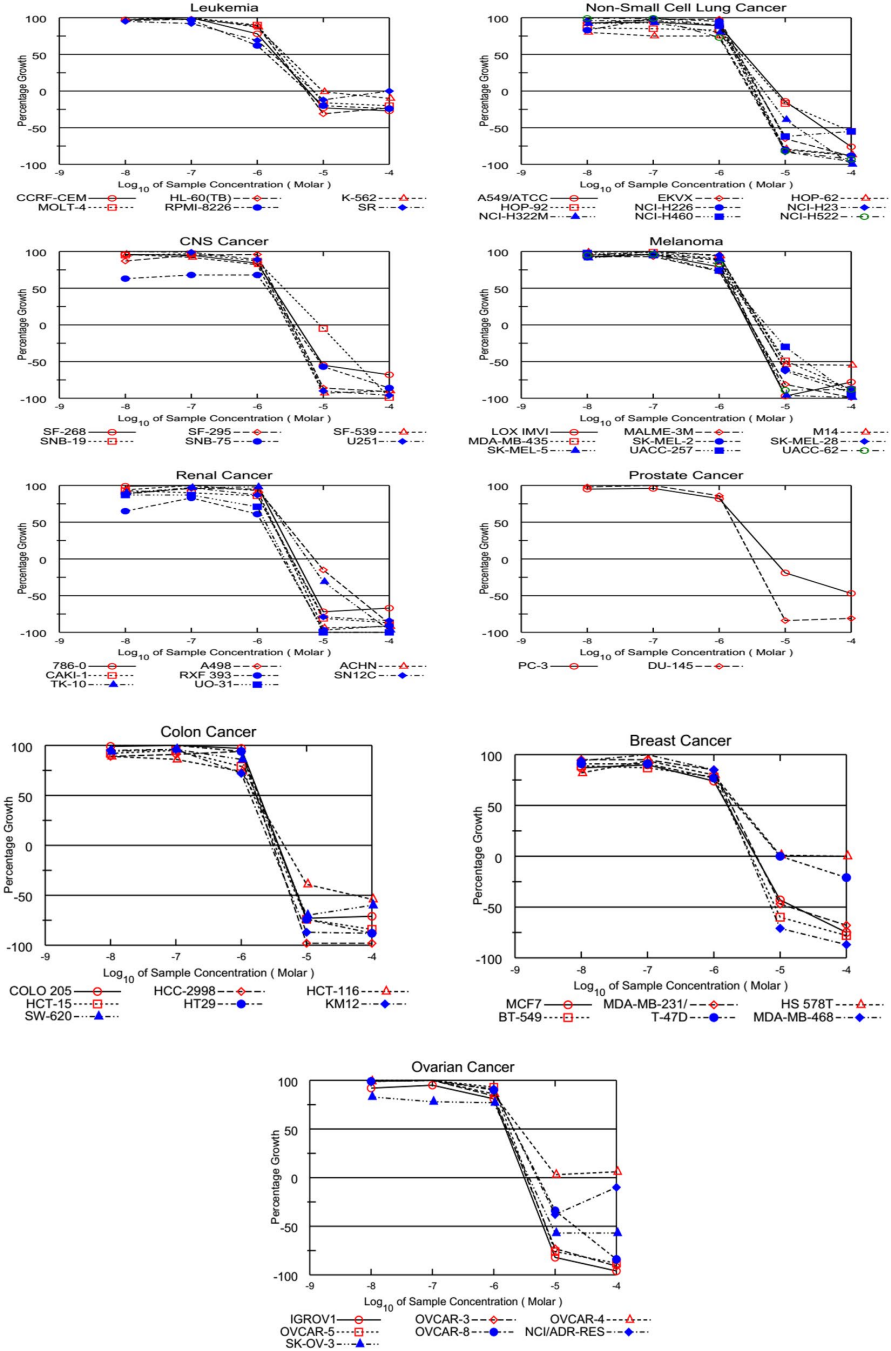
Dose-response curves for compound **CDHPM-10e**.

The GI_50s_ of the herein examined hybrids in a five-dose screen against all nine subpanels are described in Table 2, using the reference drug sorafenib. The data demonstrated that Colon HCT-15 and Renal RXF 393 tumour cells were the most susceptible cell lines to all the examined conjugates with a 1-digit micromolar GI_50_ scale. In addition, the CNS SNB-75 tumour cell was the most sensitive cell line among the herein examined 60 cell lines with a sub-micromolar GI_50_ value of 0.83 µM. Among all the tested targets, **CDHPM-10e** with hydrophilic moiety showed the highest potencies over all the herein examined 60 tumour cells with single-digit micromolar scale. Compound **CDHPM-10e** was also found to be more potent than the reference drug sorafenib for the nine types of cancer. At the same time, the least potent hybrid was found to be the lipophilic-containing moiety **CDHPM-10c**. Notably, the more hydrophilic moiety endows with, the more potent antiproliferative activity will be. Interestingly, the hybrid drug candidate **CDHPM-10e** certainly has dose-dependent activity towards the herein examined cancer cell lines (Figure 4).

###### TGI

In addition, most compounds showed variable cytostatic activity ranging from potent to no impact (Table S1, see Supplementary Material). Melanoma (MDA-MB-435 and LOX IMVI), Colon (SW-620, HT29, HCT-15, and HCT-116), Ovarian (OVCAR-4 and IGROV1), Renal (RXF 393) and Breast (MDA-MB-468) tumour cells were the most susceptible to the target analogs with potent cytostatic effect at a 1-digit micromolar scale. Notably, the hybrid **CDHPM-10e** demonstrated potent cytostatic action at a 1-digit micromolar range with TGI spanning in the interval: 2.43–9.81 µM towards 57 cells belonging to all herein tested tumour panels (Table S1). While compound **CDHPM-10e,** whose TGI > 100 µM had no cytostatic effect towards Ovarian OVCAR-4 cancer cell lines, exhibited good activity towards Breast HS578T cells with a TGI of 30.79 µM. In addition, compound **CDHPM-10e** emerged with two-fold more significant cytostatic activity (mean TGI = 4.36 µM) than sorafenib (NSC: 747 971^63^; mean TGI = 9.11 µM). In contrast, compound **CDHPM-10c** had the least cytostatic activity towards the herein explored cell lines.

###### LC_50_

Furthermore, most target compounds emerged as non-lethal agents towards most of the herein examined tumour cells. All the target analogs were found to be non-lethal towards Leukaemia (RPMI-8226, MOLT-4, K-562, HL-60(TB), CCRF-CEM and SR), Ovarian (OVCAR-4 and NCI/ADR-RES), Breast (T-47D and HS 578 T) and Prostate (PC-3) tumour cells with LC_50_ > 100 µM. Among the target compounds, **CDHPM-10e** displayed LC_50_ values spanning in the interval: 2.04–70.60 µM (Table S2, see Supplementary Material). Also, the mean LC_50_ value of **CDHPM-10e** was 26.02 µM while sorafenib (NSC: 747 971^63^) had 43.10 µM.

**Figure 5.**
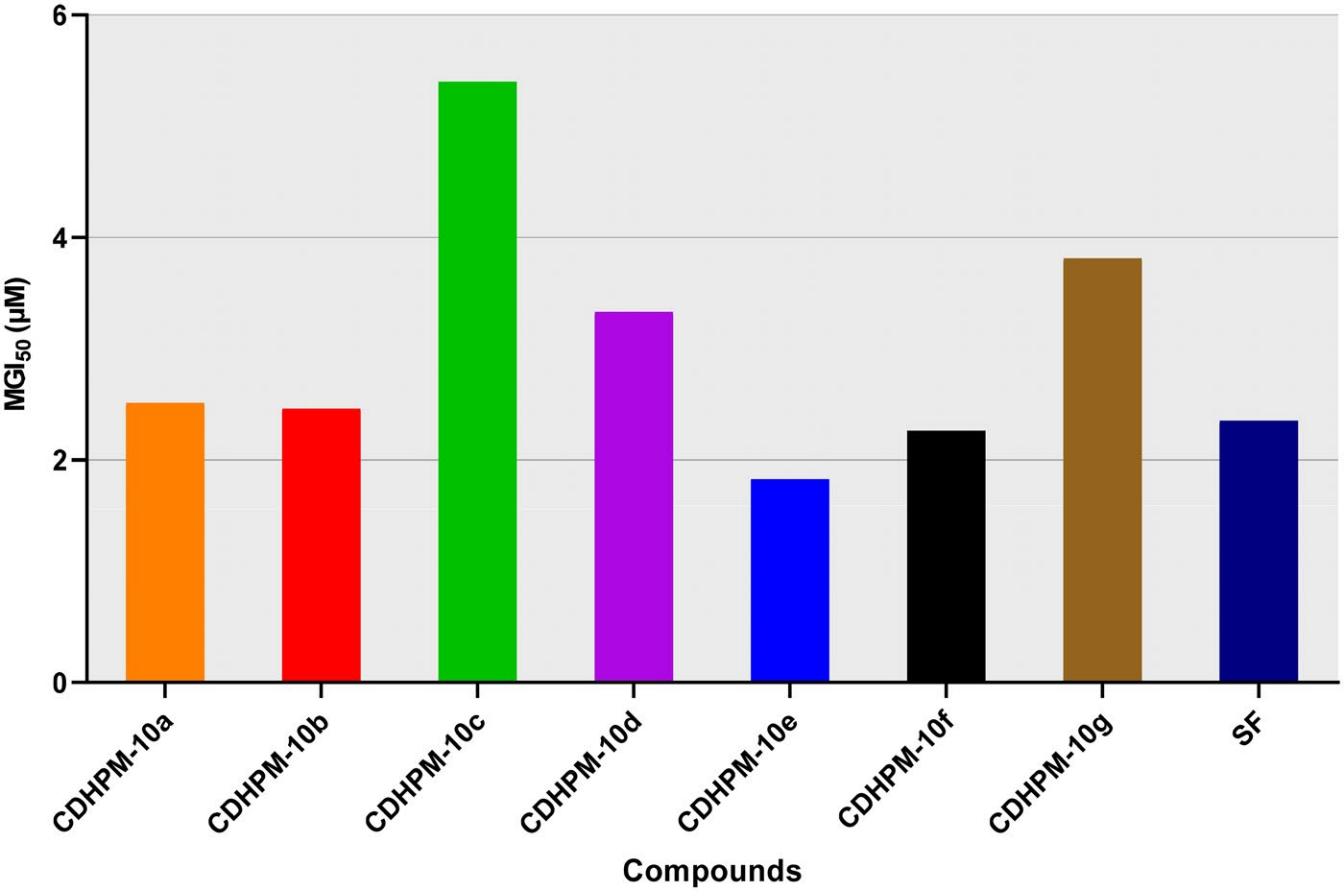
Full panel MGI_50_ of target hybrids **CDHPM-10a-g** and SF.

Moreover, all the herein tested compounds revealed high potency with a single-digit micromolar mean GI_50_ scale against all the nine cancer types (Figure 5). All the MGI_50_ values were spanning in the range from 1.83 to 5.40 µM. Of particular interest, the hybrid **CDHPM-10e** with vanillin-like moiety displayed the highest potencies towards all the herein tested subpanels with an MGI_50_ of 1.83 µM. In comparison, the bromo derivative **CDHPM-10c** was the least active hybrid among the herein examined compounds with MGI_50_ of 5.40 µM. In addition, derivative **CDHPM-10e** was more potent than sorafenib, while compounds **CDHPM-10a**, **CDHPM-10b**, and **CDHPM-10f** were approximately equipotent.

###### Selectivity index (SI)

Again, in Table 3, the SI was calculated by division of MGI_50_ (full panel) by MGI_50_ (subpanel). Non-selectivity is denoted by a value <3, while moderate and high selectivity are denoted by a range from 3 to 6 and a value >6, respectively^64^. The herein reported analogs **CDHPM-10a-g** have been exposed as potent non-selective broad-spectrum anticancer agents over all NCI subpanels with an SI range of 0.66–1.97, while sorafenib fell in the average of 0.99. Thus, the promising anti-proliferative profile of phenolic derivative **CDHPM-10e** over all the herein examined 60 tumour cells implied to explore its mechanism of action further.

**Table 3.** SI of target hybrids **CDHPM-10a-g** and the reference drug sorafenib towards nine cancer types.

Panel	CDHPM-							SF
	10a	10b	10c	10d	10e	10f	10g	
Leukaemia	1.18	1.10	1.97	1.39	0.91	1.00	1.69	1.23
NSCLC	0.99	0.87	0.69	0.74	1.00	0.95	0.66	1
Colon cancer	1.16	1.22	1.46	1.21	1.10	1.24	1.18	0.81
CNS cancer	1.14	1.01	1.36	1.20	1.03	1.28	1.25	1
Melanoma	1.21	1.23	0.90	1.15	1.06	1.19	1.12	1.26
Ovarian cancer	0.92	0.80	0.74	0.83	0.95	1.09	0.84	0.81
Renal cancer	0.76	0.75	0.71	0.73	1.02	0.77	0.79	0.82
Prostate cancer	0.79	1.03	0.85	0.94	0.98	0.80	0.87	0.91
Breast cancer	1.09	1.37	1.73	1.30	0.97	0.97	1.35	1.08

##### Structure-activity relationships (SAR)

**Figure 6.**
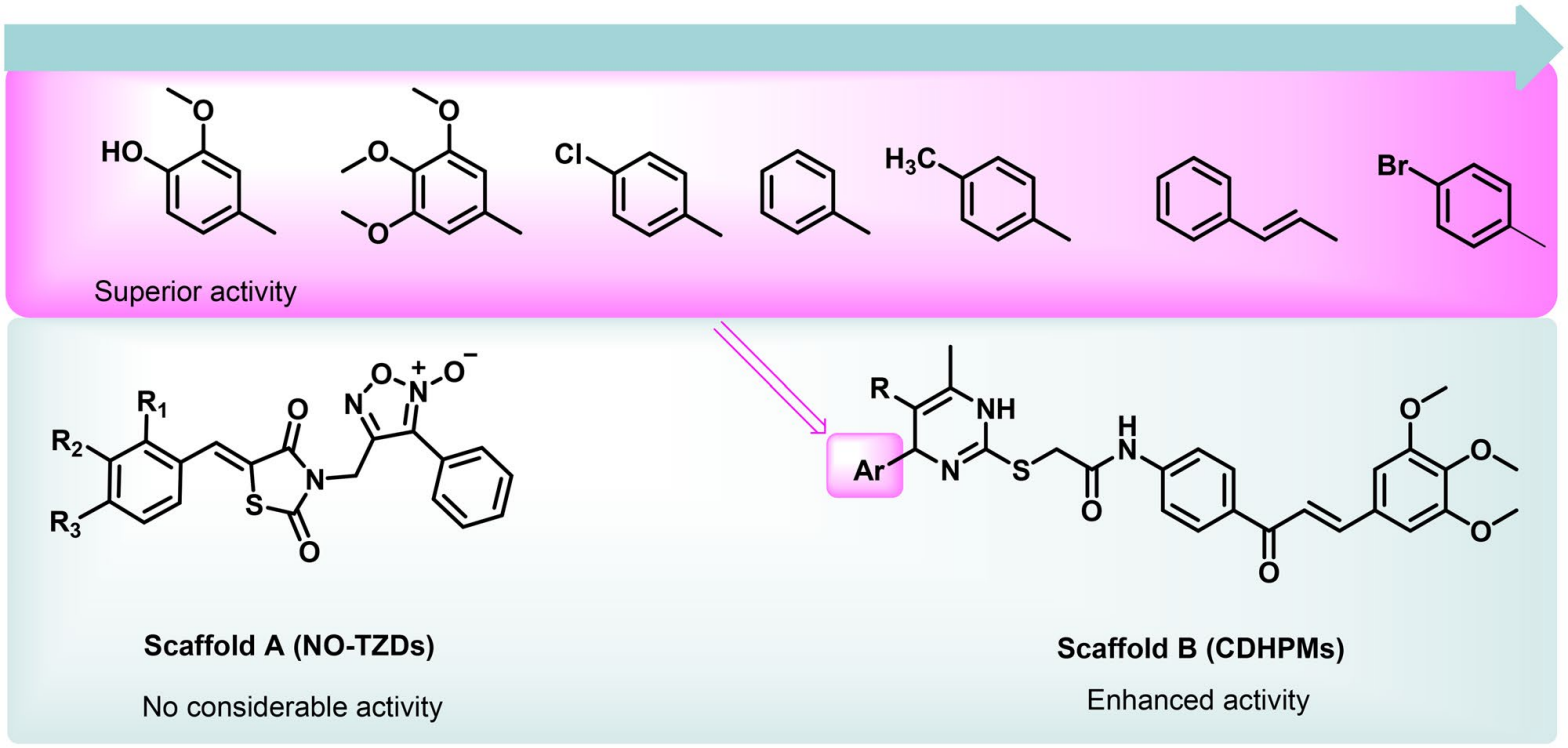
SAR study of designed hybrids **CDHPM-10a-g**.

Initially, the NO-TZDs (scaffold A) did not show considerable activity, therefore, the rational design has been directed to scaffold hopping yielding trimethoxychalcone-based dihydropyrimidines (scaffold B). This optimisation allowed better interaction within the receptor’s active site. Since the investigated analogs exhibited variable anticancer activities towards tumour cells, the SAR of scaffold “B” could be summarised as follows. Firstly, substituting the terminal phenyl group with 4-hydroxy-3-methoxyphenyl moiety (afforded compound **CDHPM-10e**) would significantly increase its anti-proliferative activity by 34.01%, while substitution with 3,4,5-trimethoxyphenyl motif as in **CDHPM-10f**, the %inhibition was increased by 26.39%. Secondly, substituting terminal phenyl moiety with chlorine atom at the para position (afforded compound **CDHPM-10b**) would increase the inhibitory activity by 3.09%. Thirdly, introducing the *p*-methyl group at the terminal phenyl group (afforded compound **CDHPM-10d**) would decrease the inhibitory activity by 2.01%. Fourthly, inserting an ethylene linker between the terminal phenyl group and the pyrimidine core, as in **CDHPM-10g**, led to a significant decrease in its anti-proliferative activity by 22.37%. In comparison, substituting the terminal phenyl moiety with a more lipophilic bromine atom at the para position, as in **CDHPM-10c**, led to a marked activity loss of 37.48%. Finally, **CDHPM-10e** established the most potent anticancer agent among the herein examined series towards the full cell panel. In brief, we could conclude that the installation of a more hydrophilic group at the terminal phenyl moiety of the pyrimidine core, which is endowed with 3,4,5-trimethoxychalcone through the *S*-acetamide bridge, could significantly improve the anti-proliferative activity as shown in Figure 6.

#### VEGFR-2 inhibitory activity

**Figure 7.**
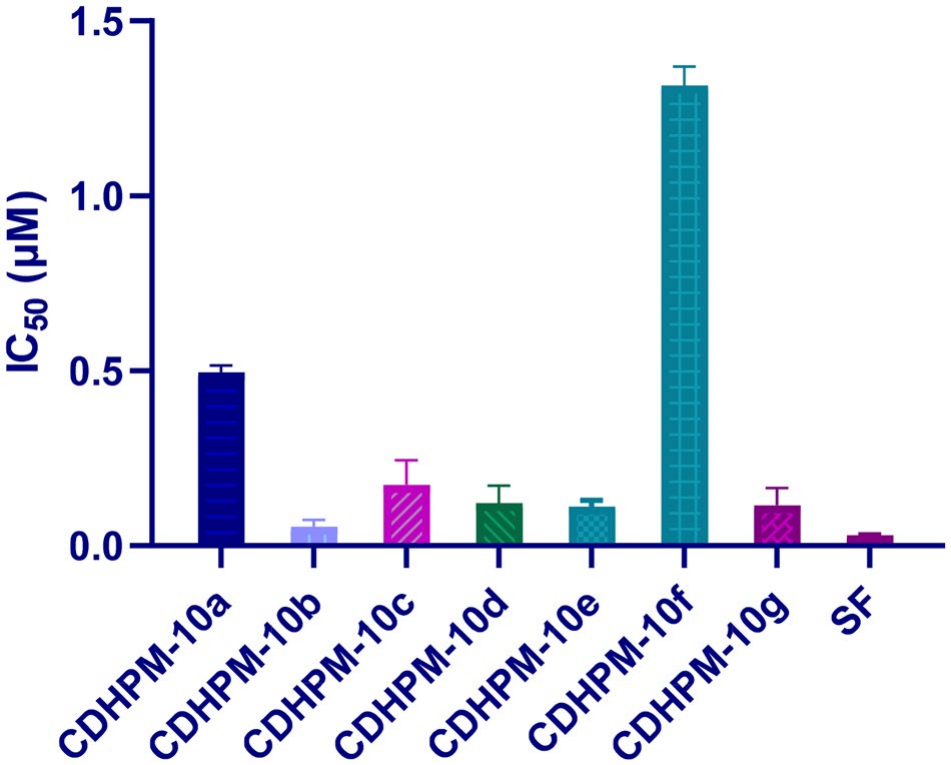
VEGFR-2 blockade of **CDHPM-10e** and the reference drug sorafenib.

The most active analogs, **CDHPM-10a-g**, were examined for their inhibitory action towards VEGFR-2 utilising VEGFR-2 Kinase assay following the manufacturer’s instruction and sorafenib as a reference. The data showed that the hybrids **CDHPM-10b-e** and **CDHPM-10g** revealed potency comparable to that of sorafenib with sub-micromolar IC_50_ values of 0.054–0.174 µM (Figure 7), demonstrating the anti-angiogenic action of them.

#### In vitro *anti-proliferative activities (SRB assay)*

The most promising analog, **CDHPM-10e**, was evaluated for its growth inhibitory activity towards the MCF-7 cells, a highly expressing VEGFR-2 kinase^12^. Serial dilutions of hybrid **CDHPM-10e** were incubated with MCF-7 cells for 72 h. SRB assay was conducted to evaluate the cell viability utilising the reference drug SF^65^. The target compound **CDHPM-10e** registered two-fold more potent anti-proliferative activity than sorafenib since the IC_50_ values were 2.52 and 5.10 µM, respectively, towards the MCF-7 cell line with a selectivity index of 0.91 (Table 4).

**Table 4.** IC_50_ values for target analog **CDHPM-10e** and sorafenib towards breast cancer cells.

Comp.	IC_50_ (µM)		Selectivity index
	MCF-7	HSF	MCF-7
CDHPM-10e	2.52 ± 0.10	2.76 ± 0.12	0.91
SF	5.1 ± 0.40^20^	>50	–

#### MTT assay

**Figure 8.**
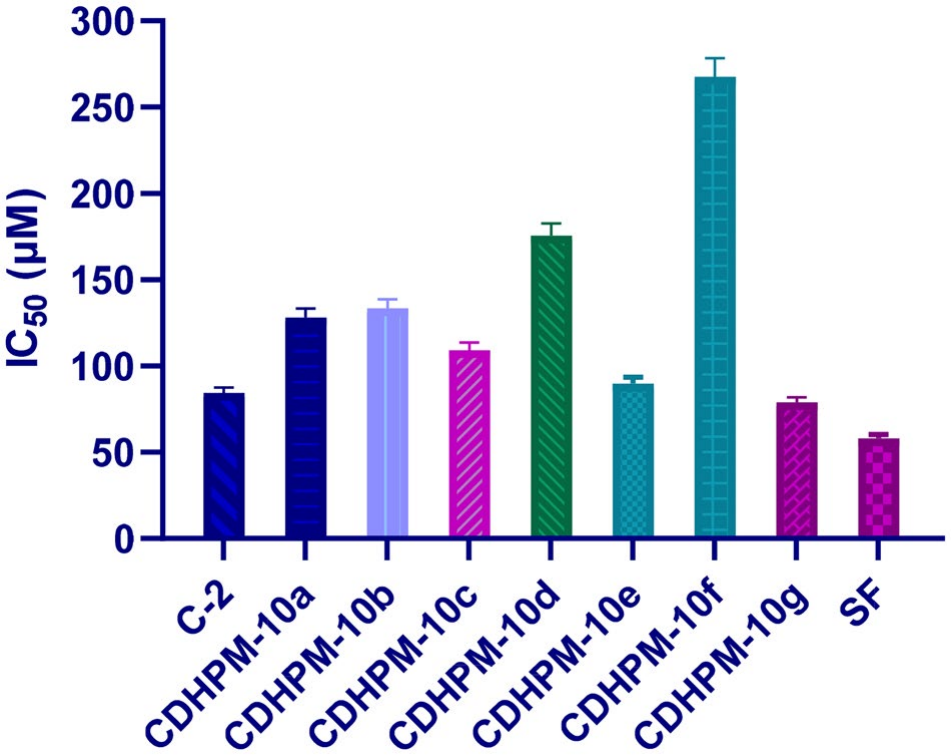
Cytotoxicity activity for chalcone chloride intermediate **C-2**, target analogs **CDHPM-10a-g** and reference drug sorafenib towards human umbilical vein endothelial cells (HUVECs).

The chalcone chloride intermediate **C-2** and the most promising analogs **CDHPM-10a-g** were evaluated for their growth inhibitory activity towards the human umbilical vein endothelial cells (HUVECs) as they are widely used as a source of primary endothelial cells for *in vitro* studies of the vasculature and angiogenesis using MTT assay along with sorafenib as a reference drug (Figure 8). Serial dilutions of chalcone chloride intermediate **C-2** and hybrids **CDHPM-10a-g** were incubated with HUVECs cells for 48 h using sorafenib as a reference. Among the target compounds, **CDHPM-10e** and **CDHPM-10g** exhibited antiproliferative activity comparable to that of the reference drug with IC_50s_ equal 89.91, 78.79, and 58.08 µM, respectively, towards the HUVECs cell line. In addition, chalcone chloride intermediate **C-2** was tested to examine the hybridisation of trimethoxy chalcone motif with dihydrpyrimidine scaffold. The results proved the importance of trimethoxy chalcone scaffold for biological activity with an IC_50_ of 84.29 µM. Furthermore, coupling the styryl derivative of DHPM with trimethoxy chalcone scaffold **C-2**, as in **CDHPM-10g**, improved the antiproliferative activity. Therefore, the results thoroughly delineate the hybrid candidates **CDHPM-10e** and **CDHPM-10g** as good lead compounds and anti-angiogenic agents for further study.

#### Cell cycle study

**Figure 9.**
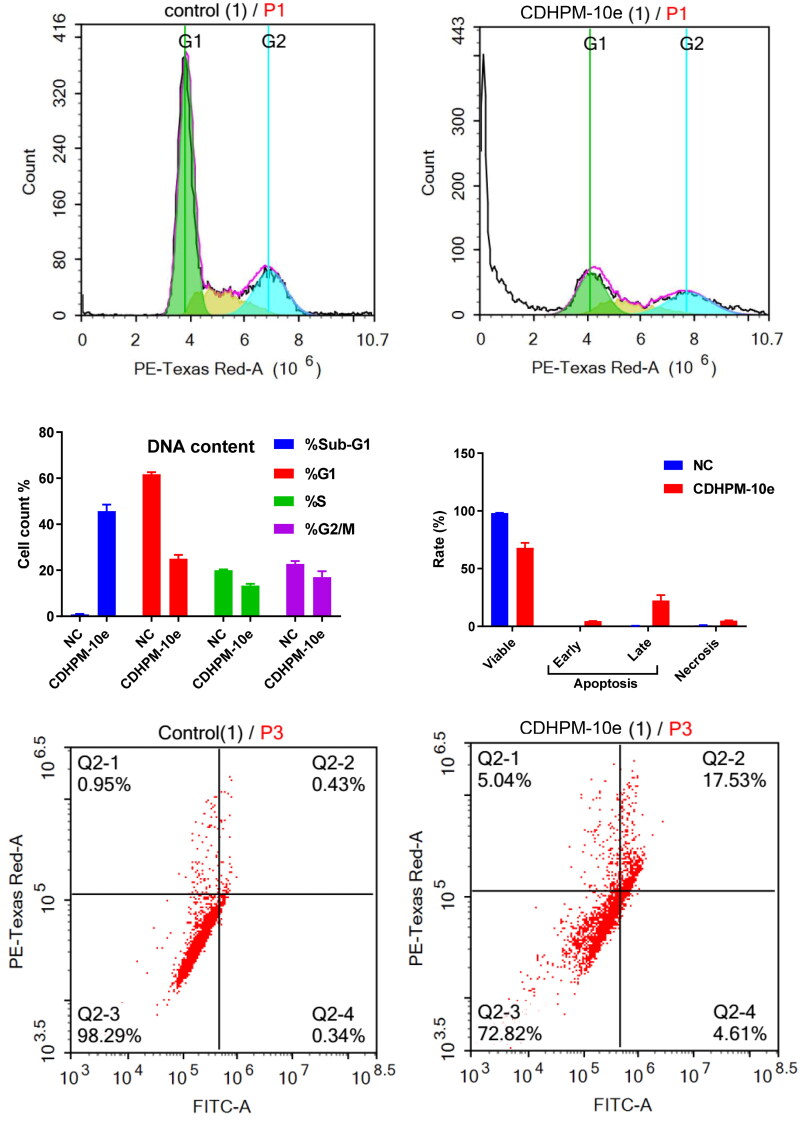
Flow cytometric studies: cell cycle analysis; (A) DMSO, (B) **CDHPM-10e**, (C) %Cells in cycle stages (G1, S, G2/M, Sub-G1); apoptosis induction; (D) histogram for induction of apoptosis, (E) NC, (F) **CDHPM-10e**.

The activity of **CDHPM-10e** on the distribution of the MCF-7 cell cycle was explored to verify its mechanism of action with propidium iodide (PI) using flow cytometry^66^^,^^67^. Cells were treated with 2.52 µM of **CDHPM-10e** and DMSO (NC) for 48 h. The data confounded that **CDHPM-10e** notably enhanced the sub-G1 phase cells compared to DMSO (negative control; NC). The %cells of the subG1/apoptosis phase increased to 45.64% for **CDHPM-10e** from 0.82% for NC (Figure 9(A–C)). Furthermore, the results revealed a decrease of the G2/M, G1, and S peaks, indicating the loss of DNA during apoptosis, thus leading to a lower PI intensity. Therefore, compound **CDHPM-10e** has the potential to induce Sub-G1-phase arrest.

#### Apoptosis analysis

The double staining assay annexin-V-FITC/propidium iodide (PI) was conducted to verify the mode of cell death induction in the MCF-7 cells at the sub-G1 area. At a concentration of 2.52 μM, the cells were treated with the examined analog **CDHPM-10e** along with NC for 24 h. The study disclosed that the total induced apoptosis percentage was 31.99%. There was an increase in the apoptotic cell rate in the early phase from 0.33% for NC up to 4.52%. In addition, there was a significant increase in the apoptotic rate in the late phase, to attain 22.50% compared to 0.49% in NC cells. These results exposed that analog **CDHPM-10e** induced the pre-G1 apoptotic stage and caused cell cycle arrest at the sub-G1 area (Figure 9(D–F)). Nonetheless, this ratio implied that the anticancer activity of derivative **CDHPM-10e** has relied on apoptosis. After 48 h, ACEA measurements employing MCF-7 revealed that **CDHPM-10e** mainly triggers apoptosis in which 22.50% of the cells were late apoptosis and 4.52% early apoptosis whereas only 4.97% were necrotic, indicating high sensitivity towards many types of cancers which augmented by its antiproliferative activity.

#### Effect of CDHPM-10e on Caspase-3 level

**Figure 10.**
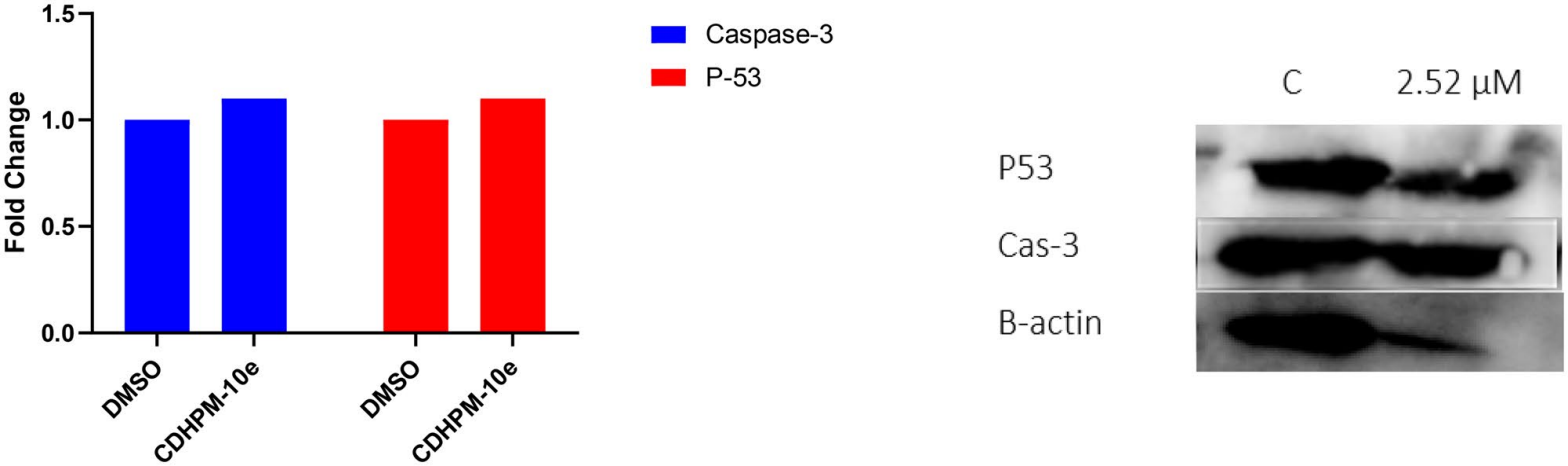
Western blot analysis of compound **CDHPM-10e** along with DMSO control.

The human active caspase-3 protein (a marker of apoptosis) level was examined utilising a western blot assay following the manufacturer’s protocol. The stimulation of Cas-3 plays a vital role in apoptosis induction. DNA fragmentation is a result of its activation through an initiator caspases^68^. It’s well known that VEGFR-2 TKIs increase the Cas-3 enzymatic activity, thus potentiating the intrinsic apoptotic cascade^68^^,^^69^. Therefore, to explore the effect of analog **CDHPM-10e** on the apoptotic Cas-3 level, the MCF-7 tumour cell line was treated with the herein examined analog at 2.52 µM for 24 h using DMSO (Negative Control; NC). The data indicated that hybrid **CDHPM-10e** exhibited a significant increase in the active Cas-3 level compared to NC (Figure 10).

#### Effect of CDHPM-10e on P53 level

P53 protein was proven to have a vital role in the induction of apoptosis, cell cycle arrest, DNA repair, and angiogenesis inhibition^70^. Further evaluation of **CDHPM-10e** at 2.52 µM towards MCF-7 cells clearly boosted the tumour suppressor protein p53 expression level (Figure 10). Therefore, the obtained data from western blot analysis exposed that compound **CDHPM-10e** might prompt apoptosis and cell cycle arrest *via* caspase and p53-dependent mechanisms.

#### Cell migration assay

**Figure 11.**
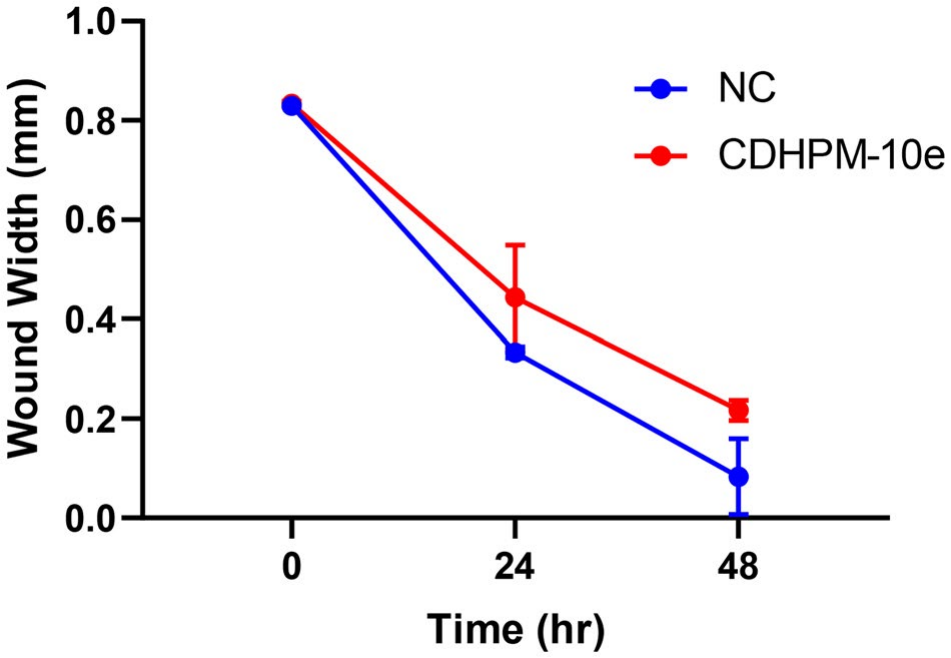
Wound healing inhibition in **CDHPM-10e**-treated MCF-7 cell line.

**Figure 12.**
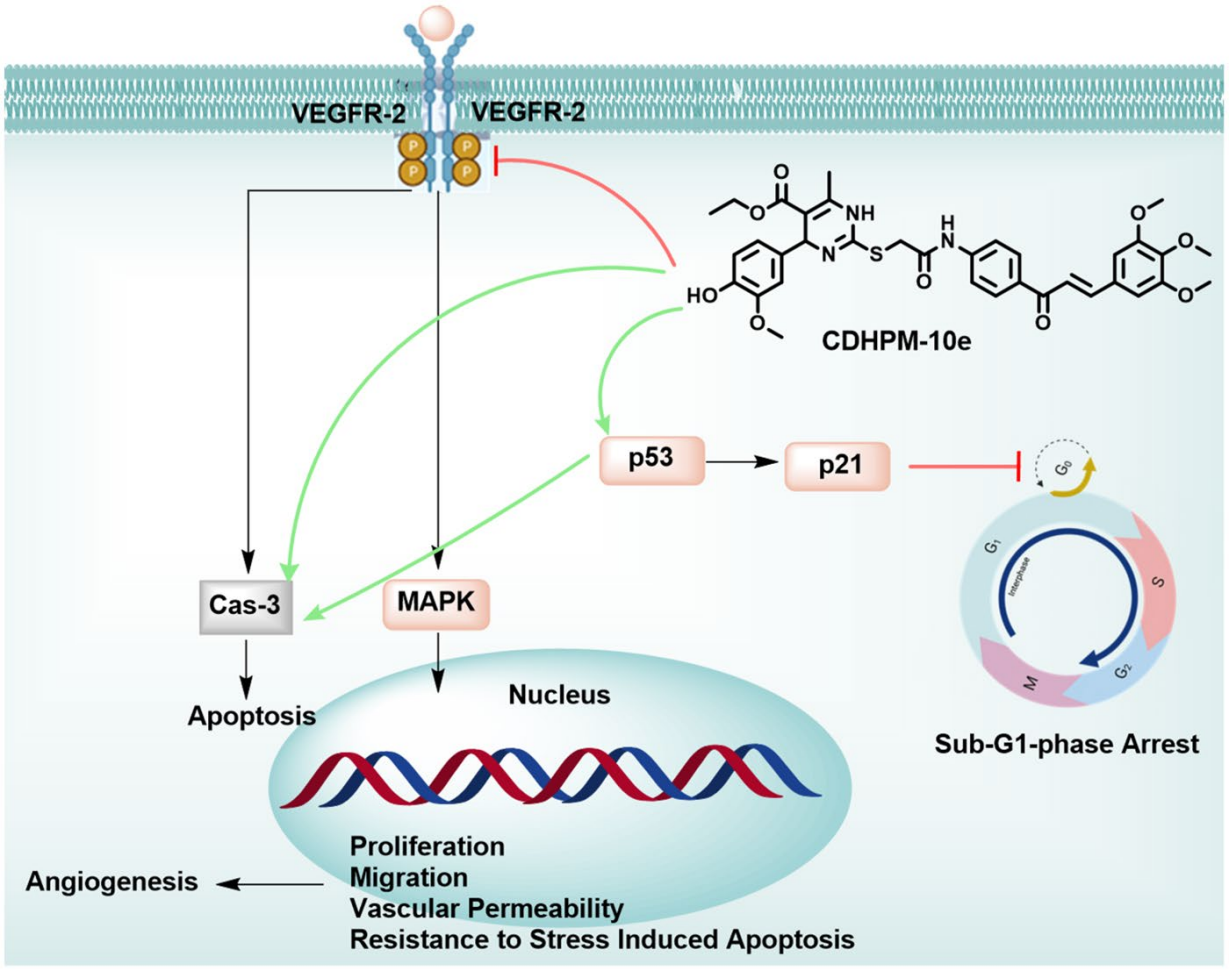
Potential anticancer mechanisms of **CDHPM-10e**.

The anti-metastatic action of **CDHPM-10e** was also investigated using a simple and economical wound-healing assay. MCF-7 cells were incubated with derivative **CDHPM-10e** in serum-free medium (SFM) for 48 h at a concentration of 2.52 µM. Figure 11 shows that hybrid **CDHPM-10e** markedly decreased the cell migration rate in SFM compared to NC cells. Therefore, the results hinted out that hybrid **CDHPM-10e** is a potent anticancer agent with anti-metastatic activity. Overall, the anticancer mechanisms of **CDHPM-10e** associated with VEGFR-2 TK inhibition are illustrated in Figure 12.

### In-silico *molecular modelling studies*

#### EON scaffold hopping

Concerning sorafenib as an existing anti-angiogenic drug, OpenEye’s EON module utilises electrostatic potential similarities and physically realistic shapes to discover new molecular targets utilising full Poisson–Boltzmann (PB) electrostatics to generate electrostatic Tanimoto (ET) grids^24^^,^^25^. The formal charges and pKa state majorly affect the electrostatics, so the database and query drug are examined in a neutral pH model. EON module bestows substantially to lead discovery and design. Results were visualised and analysed by the EON view tool of OpenEye’s VIDA visualisation module^71^. In addition, the query drug sorafenib was comprised in the library for results validation. EON ranks the designed compounds based on their electrostatic similarity and shape to the query drug. The top-scoring analogs, **NO-TZD-3a-d,5,6** and **CDHPM-10g**, offered the best alignment with the query are cited in Table 5.

**Table 5.** EON electrostatic potential similarity and shape of the herein examined analogs concerning sorafenib as a query drug.

VIDA name	ET-PB^a^	ET-Coul^b^	ET-Combo^c^	Shape Tanimoto^d^	Rank
NO-TZD-3a	0.192	0.202	0.255	0.063	1
NO-TZD-3b	0.18	0.182	0.248	0.067	2
NO-TZD-3d	0.068	0.079	0.214	0.146	3
NO-TZD-5	0.097	0.084	0.179	0.082	4
NO-TZD-3c	0.033	0.06	0.171	0.138	5
CDHPM-10g	−0.011	−0.017	0.04	0.052	6
CDHPM-10f	0.024	0.002	0.029	0.006	7
NO-TZD-6	0.017	0.017	0.017	0	8
CDHPM-10e	−0.016	−0.028	−0.011	0.004	9
CDHPM-10a	−0.019	−0.03	−0.016	0.002	10
CDHPM-10d	−0.019	−0.031	−0.017	0.002	11
CDHPM-10c	−0.021	−0.031	−0.019	0.002	12
CDHPM-10b	−0.022	−0.031	−0.019	0.002	13

^a^ Electrostatic Tanimoto utilising full Poisson–Boltzmann (PB) electrostatics.

^b^ Electrostatic Tanimoto utilising only PB electrostatics coulombic part.

^c^ Sum of EON shape Tanimoto and ET-PB.

^d^ Shape Tanimoto between sorafenib and the tested compound.

#### Docking studies

**Figure 13.**
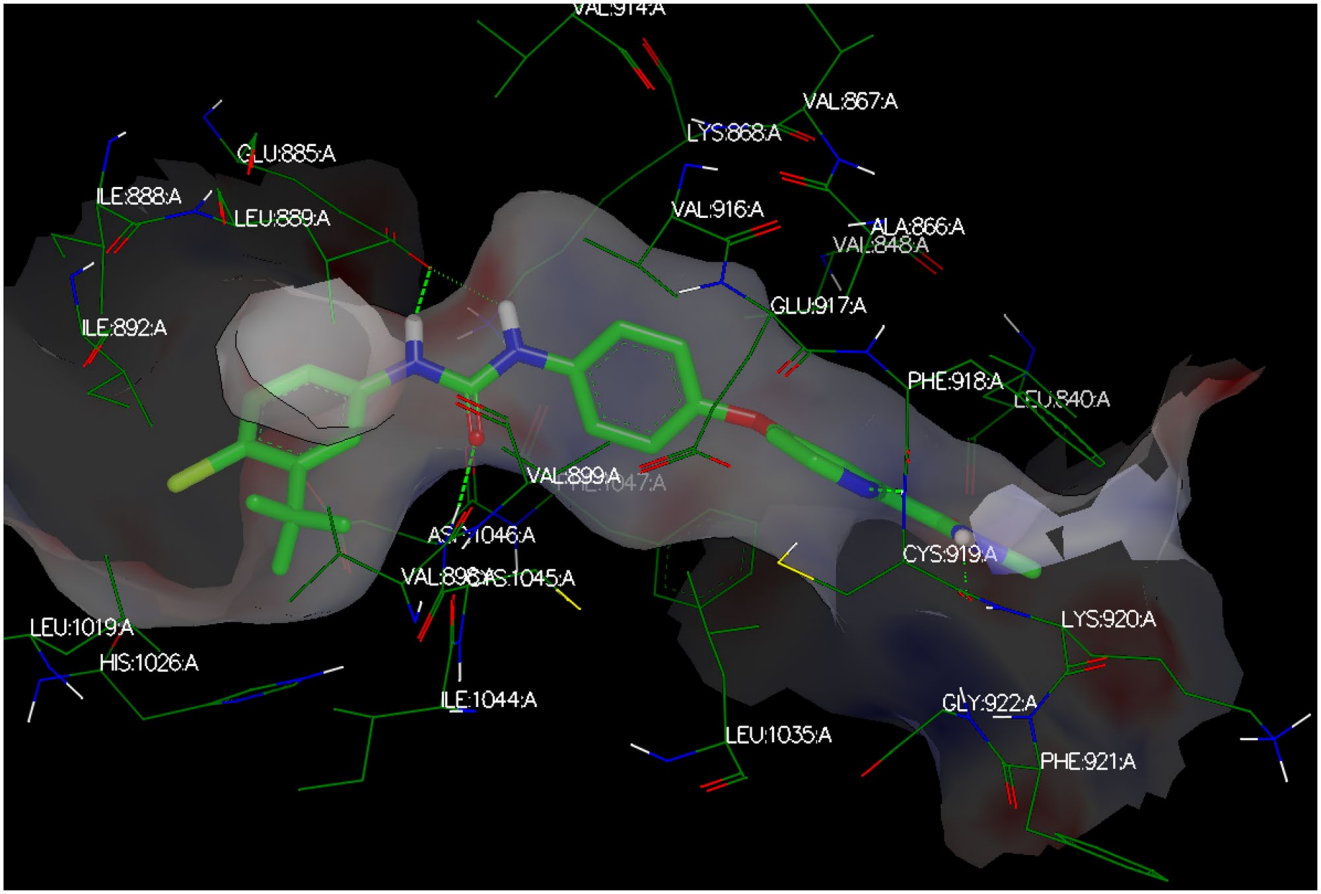
3D binding interactions of sorafenib (green) in the VEGFR-2 pocket. Green dash lines denote H-bonds.

Modelling studies utilised OpenEye^®^ Scientific Software (2023) to investigate the interacting poses of the most potent herein designed analogs, **CDHPM-10a-g**, with the KDR binding pocket whose co-crystallised drug sorafenib (PDB ID: 3WZE). The sum of hydrogen bonding, desolvation, and shape energies (FRED Chemgauss4 scores) were utilised to examine compounds in which the lowest scores indicate the best binding poses, as shown in Table 6. Sorafenib was re-docked utilising the standard method to rationalise the protein–ligand poses of the herein examined analogs in the VEGFR-2 active site and validate the results. The data demonstrated that the docking interaction of sorafenib was the same as that of the co-crystallised ligand (Figure 13).

**Table 6.** Chemgauss4 scores of the designed analogs **CDHPM-10a-g** and sorafenib.

Compound	Chemgauss4 score
CDHPM-10a	−12.6031
CDHPM-10b	−12.4090
CDHPM-10c	−12.4345
CDHPM-10d	−11.9907
CDHPM-10e	−11.1431
CDHPM-10f	−10.1780
CDHPM-10g	−11.9732
SF	−18.408

**Figure 14.**
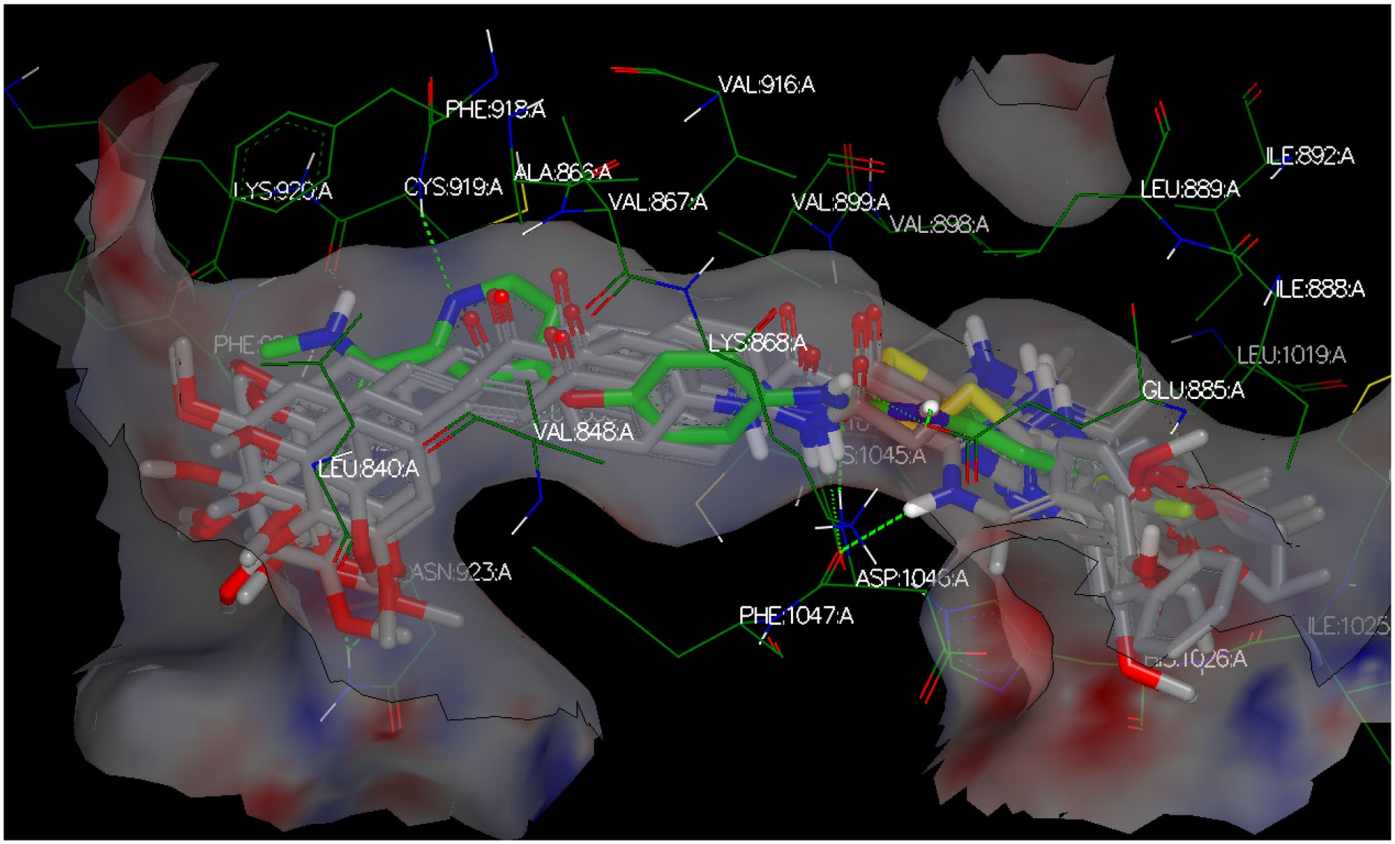
3D binding interactions show an overlay between target compounds (grey) and sorafenib (green) in the VEGFR-2 pocket. Green dash lines denote H-bonds.

The target compounds spatially stacked well together, showing an overly with sorafenib (Figure 14). Compounds **CDHPM-10d-f** displayed hydrogen bonds coming from acetamide NH and Asp:1046 residue in the DFG binding domain with lengths of 1.85, 2.09, and 2.41 Å, correspondingly, while compounds **CDHPM-10a**, **CDHPM-10b**, **CDHPM-10c**, and **CDHPM-10g** revealed mainly electrostatic and hydrophobic interactions within the active site. In addition, compounds **CDHPM-10d** and **CDHPM-10e** revealed an additional hydrogen bond coming from *m-*methoxy group of the head phenyl and NH of Asn:923:A with lengths of 2.22 and 2.12 Å, respectively. The results displayed an excellent fitting of the designed analogs to the binding pocket of KDR with FRED Chemgauss4 score interval from −10.1780 to −12.6031 compared to −18.408 for sorafenib.

**Figure 15.**
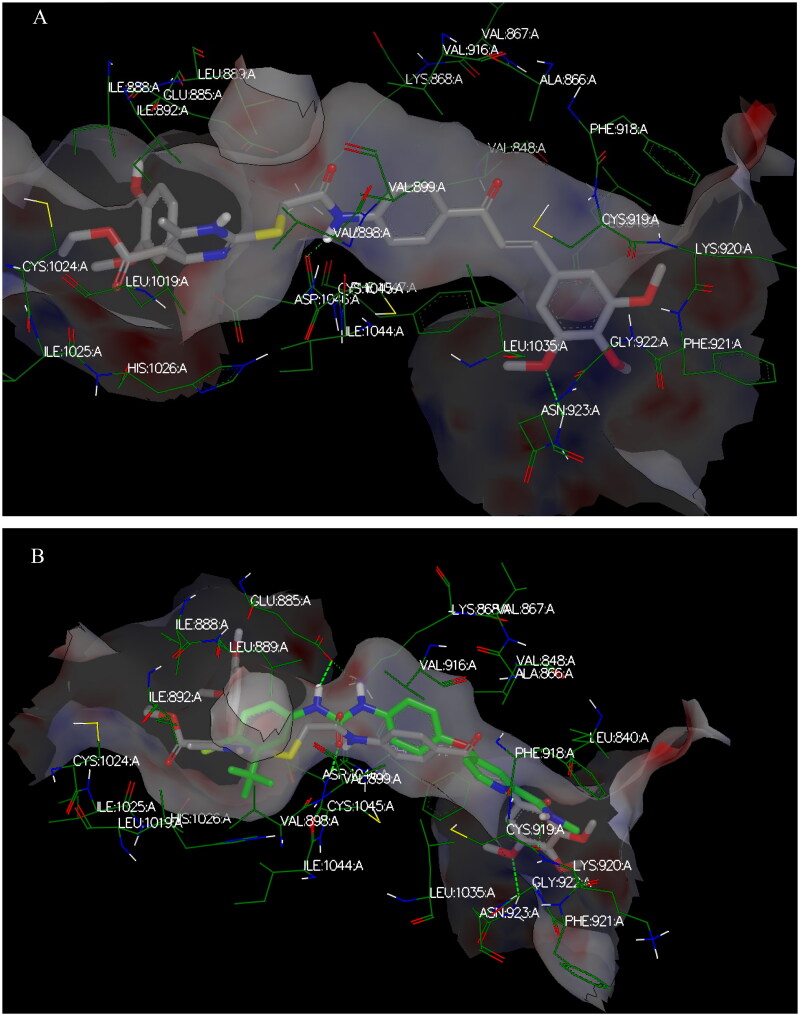
3D interactions; (A) docking pose of **CDHPM-10e** (grey) inside the binding region of VEGFR-2 demonstrating hydrogen bonding (green dash line) with Asp:1046:A and hydrophobic interactions; (B) overlay of **CDHPM-10e** with the reference drug sorafenib (green).

**Figure 16.**
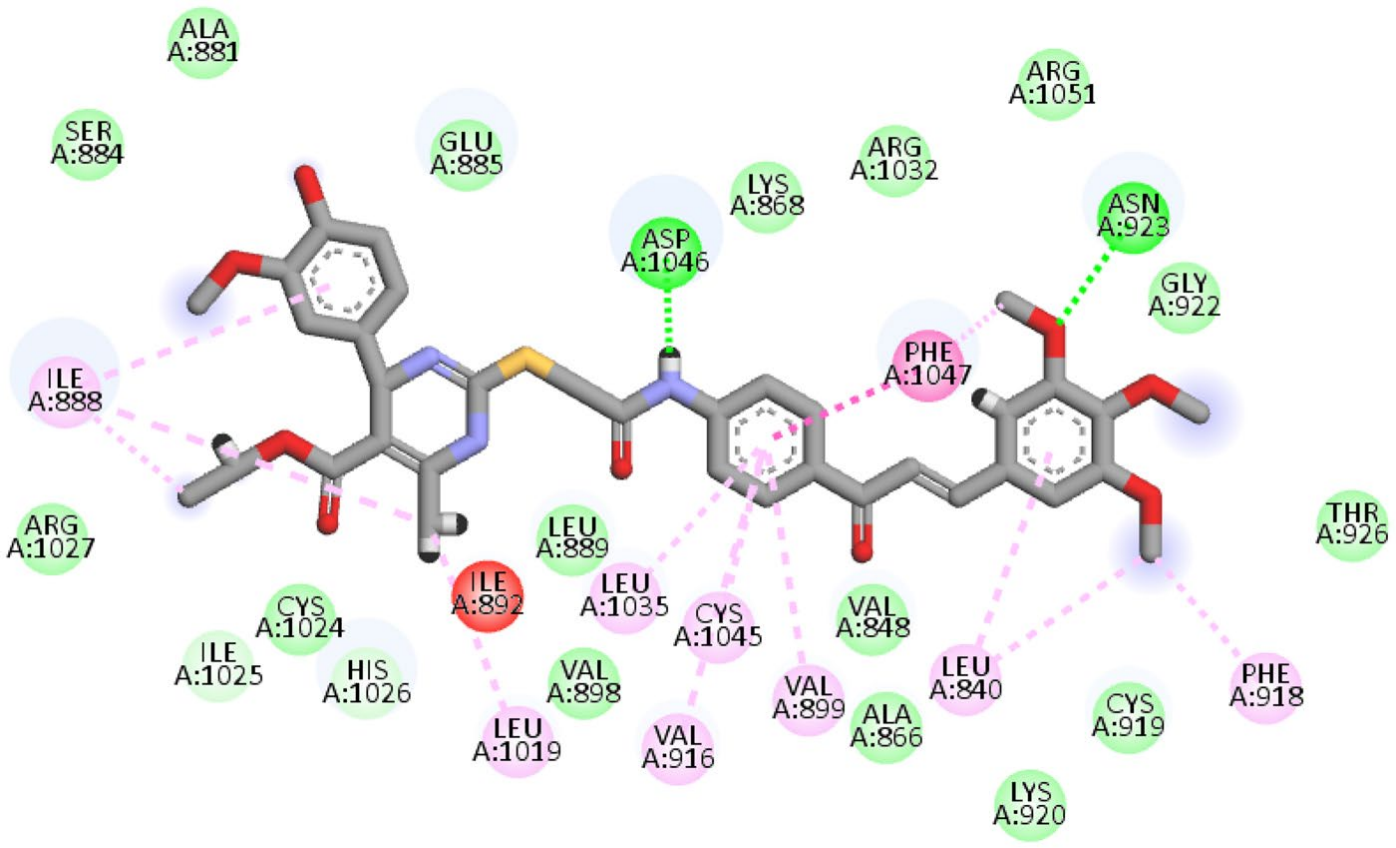
2D depiction of **CDHPM-10e** showing hydrophilic and hydrophobic interactions inside VEGFR-2 active site.

Again, the most promising hybrid towards NCI-60 tumour cells with broad-spectrum antiproliferative activity, **CDHPM-10e**, was chosen as the most plausible interacting conformation, so we studied its interacting modes within the active site. The analog **CDHPM-10e** presented a unique interacting pose that could inspire its broad-spectrum antitumor activity. As illustrated in Figure 15(A,B), compound **CDHPM-10e** presented a strong H-bond within the DFG binding region and ATB binding domain overlaid well with the reference drug, and fit tightly inside the VEGFR-2 binding region. In addition, the 2D interacting pose of the KDR with **CDHPM-10e** shown in Figure 16 was generated by Discovery Studio Visualiser^72^^,^^73^. Compound **CDHPM-10e** showed hydrophobic and electrostatic interactions with the contacted amino acids. The pyrimidine C-4 phenyl group formed Pi-alkyl hydrophobic interaction with Ile:888:A, while the linker’s phenyl group formed pi–pi T-shaped hydrophobic interactions with Phe:1047:A. Furthermore, the ethyl and *m*-methoxy groups formed alkyl interactions with Ile:888:A and Leu:840:A, respectively. Also, the linker’s phenyl group formed pi-alkyl hydrophobic interactions with Leu:1035:A, Val:899:A, Val:916, and Cys:1045:A. In addition, the two *m*-methoxy groups of the head phenyl and pyrimidine C6 methyl formed pi-alkyl hydrophobic interactions with Phe:1047:A, Phe:918:A, and Leu:1019:A. Additionally, **CDHPM-10e** developed other mixed hydrophobic and Van der Waals interactions.

Furthermore, the dock report tool provided an additional property for **CDHPM-10e** optimisation at vanillin-like moiety, pyrimidine C-5 ester group, pyrimidine N1-H, and central aryl moiety, demonstrating protein-ligand desolvation, hydrogen bonding, and shape energies, recognised by Chemgauss4 score (Figure S49, see Supplementary Material). The analog **CDHPM-10e** showed a FRED chemgauss4 score of −11.1431 comparable to that of sorafenib (-18.408). Furthermore, the modelling study implies that **CDHPM-10e** was a potent VEGFR-2 inhibitor but was less potent than sorafenib, which is aligned with the enzyme assay inhibitory data.

### Drug-likeness characteristics and ADMET prediction

Drug-likeness and pharmacokinetics ADMET features for the most promising hybrid, **CDHPM-10e**, were predicted using the ADMETlab2.0 web-based tool^74^^,^^75^. Table 7 shows that **CDHPM-10e** disclosed 2 violations from Lipinski’s rule (MW > 500, N or O > 10) while Pfizer accepted its drug-likeness properties. The ADMET prediction data exposed that **CDHPM-10e** has a good synthetic accessibility score, a high GIT absorption rate, and moderate solubility in water. Besides, it showed >20% bioavailability and >90% plasma protein binding. Additionally, it could be CYP2D6, CYP2C19, and CYP1A2 enzymes non-inhibitor, implying a low possibility of producing drug-drug interaction. Moreover, acute toxicity and carcinogenicity tests demonstrated that hybrid **CDHPM-10e** is neither toxic nor carcinogenic, proving a good profile of ADMET properties (Table 8).

**Table 7.** Lipinski’s rule of five characteristics for hybrid **CDHPM-10e**.

Parameter							Pfizer rule	SAscore
MW^a^	*n*RB^b^	Log P^c^	*n*HBA^d^	*n*HBD^e^	TPSA^f^	Log S^g^		
675.230	16	3.60	12	3	154.01	−5.48	Accepted	3.46

^a^ Molecular weight.

^b^ Rotatable bonds.

^c^ Calculated lipophilicity.

^d^ H-bond acceptors.

^e^ H-bond donors.

^f^ Total polar surface area.

^g^ Log aqueous solubility.

**Table 8.** ADMET parameters for hybrid **CDHPM-10e**.

Absorption		Distribution			Metabolism	Excretion	Toxicity	
HIA%^a^	F_20%_^b^	PPB%^c^	VD^d^	BBB^e^	CYP1A2 CYP2C19 CYP2D6 Inhibition	CL^f^	Carcinogenicity	Acute toxicity rule
≥90	≥60	98.56	0.26	−ve	Non	11.44	Non	Non

^a^ Human intestinal absorption.

^b^ Bioavailability.

^c^ Plasma protein binding.

^d^ Volume distribution.

^e^ Blood-brain barrier penetration.

^f^ Clearance.

## Materials and methods

### Chemistry

^1^H and ^13^C NMR spectra were acquired on a Bruker AVANCE and Varian Unity INOVA-400 MHz NMR spectrometer. TLC was applied on pre-coated silica gel 60 F254 (Merck) sheets and visualised by UV (254 nm). Column chromatography was used to purify the synthesised compounds using silica gel 150–250 μm. All melting points were measured on digital melting point device (FALC Instruments s.r.l., Model MPD-03, Italy) and were uncorrected. Mass spectra were carried out on Direct Inlet part to mass analyser in Thermo Scientific GC-MS model ISQ (Waltham, MA, USA). Vario elemental analyser III (Vario, Germany) was used for C, H, and N analysis. The results agreed with the designed compounds within ±0.4% of the theoretical values. All reagents and solvents were acquired from commercially available suppliers. The intermediates **TZD-1**^76^^,^^77^, **TZD-2a-d**^43^^,^^45^^,^^46^, **TZD-4**^43^, **DHPM-7a-g**^48–53^, **C-8**^48–53^, **C-9**^56–58^ were prepared as previously described.

#### General procedure for synthesis of NO-TZD-3a-d and NO-TZD-5

To a stirred solution of TZDs **2a-d** and **4** (1 mmol) in dry DMF (2 ml), furoxan mesylate (1.1 mmol, 0.297 g) and K_2_CO_3_ (1 mmol, 0.138 g) were added. The reaction mixture was heated at *65 °C* for 1 h as indicated by TLC. The crude mixture was poured onto ice-cold water (50 ml) and then decanted. The resulting crude product was boiled in 70% ethanol and then filtered, dried, and crystallised from absolute ethanol to afford the target compounds **NO-TZD-3a-d** and **NO-TZD-5** in good yields.

##### (*Z*)-4-((5-benzylidene-2,4-dioxothiazolidin-3-yl)methyl)-3-phenyl-1,2,5-oxadiazole 2-oxide (NO-TZD-3a)

Yield (75%); yellow powder; Melting point: 169.6–170.4 °C; ^1^H NMR (400 MHz, DMSO-*d_6_*) *δ* 7.93 (s, 1H, CH-Olefinic), 7.81 (dd, *J* = 7.4, 1.9 Hz, 2H, Ar-H), 7.69–7.59 (m, 5H, Ar-H), 7.54 (q, *J* = 7.5, 6.9 Hz, 3H, Ar-H), 5.04 (s, 2H, CH_2_); ^13^C NMR (101 MHz, DMSO) *δ* 167.10, 165.27, 157.74, 134.60, 133.13, 131.82, 131.44, 130.72, 129.92, 129.76, 128.55, 126.06, 120.51, 112.03, 34.92; GC-MS m/z calcd for [M]^+^ C_19_H_13_N_3_O_4_S: 379.39, found: 379.57.

##### (*Z*)-4-((5–(4-chlorobenzylidene)-2,4-dioxothiazolidin-3-yl)methyl)-3-phenyl-1,2,5-oxadiazole 2-oxide (NO-TZD-3b)

Yield (86%); yellowish white powder; Melting point: 162.6–163 °C; ^1^H NMR (400 MHz, DMSO-*d_6_*) *δ* 7.93 (s, 1H, CH-Olefinic), 7.81 (d, *J* = 7.0 Hz, 2H, Ar-H), 7.62 (d, *J* = 5.2 Hz, 7H, Ar-H), 5.03 (s, 2H, CH_2_); ^13^C NMR (101 MHz, DMSO) *δ* 166.83, 165.15, 157.71, 136.05, 133.27, 132.31, 132.03, 131.80, 129.97, 129.74, 128.54, 126.07, 121.29, 111.94, 34.96; GC-MS m/z calcd for [M]^+^ C_19_H_12_ClN_3_O_4_S: 413.83, found: 413.75.

##### (*Z*)-4-((5–(2-hydroxybenzylidene)-2,4-dioxothiazolidin-3-yl)methyl)-3-phenyl-1,2,5-oxadiazole 2-oxide (NO-TZD-3c)

Yield (51%); yellow powder; Melting point: 201.1–201.5 °C; ^1^H NMR (400 MHz, DMSO-*d_6_*) *δ* 10.68 (s, 1H, OH, D_2_O exchangeable), 8.09 (s, 1H, CH-Olefinic), 7.84–7.80 (m, 2H, Ar-H), 7.63 (dd, *J* = 9.2, 6.4 Hz, 3H, Ar-H), 7.55 (dd, *J* = 8.1, 6.4 Hz, 1H, Ar-H), 7.37–7.31 (m, 1H, Ar-H), 6.99–6.94 (m, 1H, Ar-H), 6.77 (t, *J* = 5.6 Hz, 1H, Ar-H), 5.02 (s, 2H, CH_2_); ^13^C NMR (101 MHz, DMSO) *δ* 167.44, 165.51, 157.90, 133.39, 131.81, 131.54, 129.76, 129.54, 128.55, 128.37, 120.30, 120.02, 118.97, 116.72, 114.70, 112.08, 34.84; GC-MS m/z calcd for [M]^+^ C_19_H_13_N_3_O_5_S: 395.39, found: 395.48.

##### (Z)-4-((5–(4-hydroxy-3-methoxybenzylidene)-2,4-dioxothiazolidin-3-yl)methyl)-3-phenyl-1,2,5-oxadiazole 2-oxide (NO-TZD-3d)

Yield (55%); yellow powder; Melting point: 164–164.5 °C; ^1^H NMR (400 MHz, DMSO-*d_6_*) *δ* 7.97 (s, 1H, OH, D_2_O exchangeable), 7.96 (s, 1H, CH-Olefinic), 7.80 (d, *J* = 7.2 Hz, 2H, Ar-H), 7.61 (q, *J* = 7.5, 6.9 Hz, 4H, Ar-H), 7.14 (s, 1H, Ar-H), 7.07 (d, *J* = 8.2 Hz, 1H, Ar-H), 6.89 (d, *J* = 8.3 Hz, 1H, Ar-H), 5.02 (s, 2H, CH_2_), 3.81 (s, 3H, OCH_3_); ^13^C NMR (101 MHz, DMSO) *δ* 167.30, 165.37, 157.73, 148.71, 135.45, 131.81, 129.74, 129.66, 128.53, 128.41, 126.05, 125.33, 116.90, 114.71, 112.12, 56.06, 34.81; GC-MS m/z calcd for [M]^+^ C_20_H_15_N_3_O_6_S: 425.42, found: 425.62.

##### 4-(((*Z*)-2,4-dioxo-5-((*E*)-3-phenylallylidene)thiazolidin-3-yl)methyl)-3-phenyl-1,2,5-oxadiazole 2-oxide (NO-TZD-5)

Yield (62%); yellow powder; Melting point: 137.6–138.5 °C; ^1^H NMR (400 MHz, DMSO-*d_6_*) *δ* 7.81–7.78 (m, 1H, CH-Olefinic), 7.71–7.68 (m, 1H, Ar-H), 7.62 (ddd, *J* = 9.1, 6.0, 3.5 Hz, 4H, Ar-H), 7.42 (d, *J* = 7.1 Hz, 3H, Ar-H), 7.36 (d, *J* = 15.1 Hz, 2H, Ar-H), 6.99 (dd, *J* = 15.2, 11.5 Hz, 2H, 2 x CH-Olefinic), 5.00 (s, 2H, CH_2_); ^13^C NMR (101 MHz, DMSO) *δ* 167.00, 164.71, 157.69, 145.40, 135.88, 134.94, 131.82, 130.52, 129.74, 129.43, 128.52, 126.01, 123.55, 121.52, 112.06, 34.82; GC-MS m/z calcd for [M]^+^ C_21_H_15_N_3_O_4_S: 405.43, found: 405.46.

#### Synthesis of (Z)-4-((5–(4-(allyloxy)-3-methoxybenzylidene)-2,4-dioxothiazolidin-3-yl)methyl)-3-phenyl-1,2,5-oxadiazole 2-oxide (NO-TZD-6)

To a stirred solution of **NO-TZD-3c** (1 mmol, 0.425 g) in DMF (2 ml), allyl bromide (5 mmol, 0.42 ml), K_2_CO_3_ (1 mmol, 0.138 g) were added. The reaction mixture was heated at 80 °C for 1 h. The crude mixture was poured onto ice-cold water, filtered, dried, and crystallised from ethanol absolute to afford *O*-allyl derivative **NO-TZD-6** in good yield.

Yield (53%); yellow powder; Melting point: 154.4–155 °C; ^1^H NMR (400 MHz, DMSO-*d_6_*) *δ* 7.96 (s, 1H, CH-Olefinic), 7.83 (d, *J* = 22.2 Hz, 2H, Ar-H), 7.68–7.56 (m, 3H, Ar-H), 7.32–7.03 (d, *J* = 23.0 Hz, 3H, Ar-H), 6.16–5.94 (m, 1H, CH-Olefinic), 5.42 (d, *J* = 17.2 Hz, 1H, CH-Olefinic), 5.34–5.19 (m, 1H, CH-Olefinic), 5.03 (s, 2H, NCH_2_), 4.75–4.54 (m, 2H, OCH_2_), 3.82 (s, 3H, OCH_3_); ^13^C NMR (101 MHz, DMSO) *δ* 167.16, 165.30, 157.72, 150.55, 149.63, 134.91, 133.66, 131.81, 129.74, 128.54, 126.04, 125.96, 124.41, 118.60, 117.42, 114.22, 113.94, 112.05, 69.40, 56.12, 34.89; GC-MS m/z calcd for [M]^+^ C_23_H_19_N_3_O_6_S: 465.10, found: 465.03.

#### General procedure for the synthesis of the designed analogs CDHPM-10a-g

To a mixture of monastrol analogs **DHPM-7a-g** (1 mmol) in DMF (3 ml), K_2_CO_3_ (1 mmol, 0.138 g), NaI (0.050 g), and trimethoxychalcone chloride **C-9** (1 mmol, 0.389 g) were added. The chemical mixture was stirred at RT for 2 h as monitored by TLC and then added portion-wise to ice-cold water. The formed precipitate was filtered off and dried. Column chromatography (EtAc: Hex 1:1) afforded the target analogs **CDHPM-10a-g** in good yields.

##### Ethyl (*R,E*)-6-methyl-2-((2-oxo-2-((4–(3-(3,4,5-trimethoxyphenyl)acryloyl)phenyl)amino)ethyl)thio)-4-phenyl-1,4-dihydropyrimidine-5-carboxylate (CDHPM-10a)

Yield (85%); Light yellow powder; Melting point: 99–100 °C; ^1^H NMR (DMSO-*d_6_*, *δ* = ppm, 400 MHz) *δ* 10.59 (s, 1H, NH amide), 9.82 (s, 1H, Py-NH), 8.15 (d, *J* = 7.9 Hz, 2H, Ar-H), 7.91 (d, *J* = 15.2 Hz, 1H, COCH = C*H*), 7.72–7.66 (m, 3H, Ar-H and COC*H*=CH), 7.25–7.22 (m, 7H, Ar-H), 5.55 (s, 1H, Py-CH), 4.07–3.96 (m, 4H, SCH_2_ and CH_3_CH_2_), 3.89 (s, 6H, two *m*-OCH_3_), 3.74 (s, 3H, *p*-OCH_3_), 2.25 (s, 3H, Py-CH_3_), 1.14 (t, *J* = 7.1 Hz, 3H, CH_3_CH_2_); ^13^C NMR (DMSO-*d_6_*, *δ* = ppm, 101 MHz) *δ* 187.98, 167.47, 166.67, 153.61, 150.58, 146.23, 145.71, 144.38, 143.65, 140.23, 132.98, 130.81, 130.33, 128.98, 128.61, 127.18, 127.05, 126.67, 121.65, 118.90, 107.00, 99.20, 60.63, 59.61, 59.35, 56.64, 35.48, 17.98, 14.60; GC-MS m/z calcd for [M]^+^ C_34_H_35_N_3_O_7_S: 629.73, found: 629.46; Anal. Calcd.: C, 64.85; H, 5.60; N, 6.67; Found: C, 65.07; H, 5.74; N, 6.89.

##### Ethyl (*R,E*)-4–(4-chlorophenyl)-6-methyl-2-((2-oxo-2-((4–(3-(3,4,5-trimethoxyphenyl)acryloyl)phenyl)amino)ethyl)thio)-1,4-dihydropyrimidine-5-carboxylate (CDHPM-10b)

Yield (83%); Light yellow powder; Melting point: 163.2–164 °C; ^1^H NMR (DMSO-*d_6_*, *δ* = ppm, 400 MHz) *δ* 10.51 (s, 1H, NH amide), 9.86 (s, 1H, Py-NH), 8.14 (d, *J* = 8.3 Hz, 2H, Ar-H), 7.90 (d, *J* = 15.2 Hz, 1H, COCH = C*H*), 7.69 (d, *J* = 15.3 Hz, 1H, COC*H*=CH), 7.63 (d, *J* = 8.4 Hz, 2H, Ar-H), 7.24 (s, 2H, Ar-H), 7.21 (s, 4H, Ar-H), 5.51 (s, 1H, Py-CH), 4.01 (q, *J* = 15.4, 11.9 Hz, 4H, SCH_2_ and CH_3_CH_2_), 3.88 (s, 6H, two *m*-OCH_3_), 3.73 (s, 3H, *p*-OCH_3_), 2.25 (s, 3H, Py-CH_3_), 1.12 (t, *J* = 7.0 Hz, 3H, CH_3_CH_2_); ^13^C NMR (DMSO-*d_6_*, *δ* = ppm, 101 MHz) *δ* 187.92, 167.40, 166.50, 153.60, 150.69, 146.58, 144.61, 144.31, 143.62, 140.16, 132.94, 131.73, 130.82, 130.29, 128.94, 128.60, 128.55, 121.61, 118.85, 106.93, 98.67, 60.62, 59.67, 58.86, 56.61, 35.44, 18.00, 14.59; GC-MS m/z calcd for [M]^+^ C_34_H_34_ClN_3_O_7_S: 663.18, found: 663.82; Anal. Calcd.: C, 61.49; H, 5.16; N, 6.33; Found: C, 61.65; H, 5.37; N, 6.59.

##### Ethyl (*R,E*)-4–(4-bromophenyl)-6-methyl-2-((2-oxo-2-((4–(3-(3,4,5-trimethoxyphenyl)acryloyl)phenyl)amino)ethyl)thio)-1,4-dihydropyrimidine-5-carboxylate (CDHPM-10c)

Yield (81%); Yellow powder; Melting point: 110–111 °C; ^1^H NMR (DMSO-*d_6_*, *δ* = ppm, 400 MHz) *δ* 10.53 (s, 1H, NH amide), 9.87 (s, 1H, Py-NH), 8.15 (d, *J* = 8.4 Hz, 2H, Ar-H), 7.90 (d, *J* = 15.5 Hz, 1H, COCH = C*H*), 7.69 (d, *J* = 15.4 Hz, 1H, COC*H*=CH), 7.63 (d, *J* = 8.3 Hz, 2H, Ar-H), 7.35 (d, *J* = 8.0 Hz, 2H, Ar-H), 7.24 (s, 2H, Ar-H), 7.15 (d, *J* = 8.1 Hz, 2H, Ar-H), 5.50 (s, 1H, Py-CH), 4.06–3.97 (m, 4H, SCH_2_ and CH_3_CH_2_), 3.88 (s, 6H, two *m*-OCH_3_), 3.73 (s, 3H, *p*-OCH_3_), 2.25 (s, 3H, Py-CH_3_), 1.12 (t, *J* = 7.1 Hz, 3H, CH_3_CH_2_); ^13^C NMR (DMSO-*d_6_*, *δ* = ppm, 101 MHz) *δ* 187.91, 167.40, 166.49, 153.59, 150.73, 146.61, 145.00, 144.30, 143.63, 140.13, 132.93, 131.47, 130.82, 130.30, 129.32, 121.61, 120.26, 118.85, 106.91, 98.57, 60.62, 59.69, 58.90, 56.61, 35.43, 18.00, 14.59; GC-MS m/z calcd for [M]^+^ C_34_H_34_BrN_3_O_7_S: 707.13, found: 707.06; Anal. Calcd.: C, 57.63; H, 4.84; N, 5.93; Found: C, 57.90; H, 5.01; N, 6.12.

##### Ethyl (*R,E*)-6-methyl-2-((2-oxo-2-((4–(3-(3,4,5-trimethoxyphenyl)acryloyl)phenyl)amino)ethyl)thio)-4-(*p*-tolyl)-1,4-dihydropyrimidine-5-carboxylate (CDHPM-10d)

Yield (83%); Light yellow powder; Melting point: 114.7–115.6 °C; ^1^H NMR (DMSO-*d_6_*, *δ* = ppm, 400 MHz) *δ* 10.53 (s, 1H, NH amide), 9.77 (s, 1H, Py-NH), 8.14 (d, *J* = 8.4 Hz, 2H, Ar-H), 7.90 (d, *J* = 15.6 Hz, 1H, COCH = C*H*), 7.70 (d, *J* = 15.4 Hz, 1H, COC*H*=CH), 7.62 (d, *J* = 8.3 Hz, 2H, Ar-H), 7.25 (s, 2H, Ar-H), 7.09 (d, *J* = 7.6 Hz, 2H, Ar-H), 7.00 (d, *J* = 7.8 Hz, 2H, Ar-H), 5.49 (s, 1H, Py-CH), 4.04–3.96 (m, 4H, SCH_2_ and CH_2_CH_3_), 3.89 (s, 6H, two *m*-OCH_3_), 3.74 (s, 3H, *p*-OCH_3_), 2.24 (s, 3H, Py-CH_3_), 2.21 (s, 3H, *p*-CH_3_), 1.14 (t, *J* = 7.0 Hz, 3H, CH_3_CH_2_); ^13^C NMR (DMSO-*d_6_*, *δ* = ppm, 101 MHz) *δ* 187.93, 167.52, 166.67, 153.60, 150.32, 145.97, 144.35, 143.62, 142.88, 140.18, 136.22, 132.92, 130.81, 130.26, 129.49, 129.17, 127.01, 126.61, 121.60, 118.89, 106.96, 99.35, 60.62, 59.56, 59.17, 56.62, 35.40, 21.09, 17.96, 14.61, 14.58; GC-MS m/z calcd for [M]^+^ C_35_H_37_N_3_O_7_S: 643.76, found: 643.51; Anal. Calcd.: C, 65.30; H, 5.79; N, 6.53; Found: C, 65.24; H, 5.98; N, 6.71.

##### Ethyl (*R,E*)-4–(4-hydroxy-3-methoxyphenyl)-6-methyl-2-((2-oxo-2-((4–(3-(3,4,5-trimethoxyphenyl)acryloyl)phenyl)amino)ethyl)thio)-1,4-dihydropyrimidine-5-carboxylate (CDHPM-10e)

Yield (61%); Light yellow powder; Melting point: 111.6–112 °C; ^1^H NMR (DMSO-*d_6_*, *δ* = ppm, 400 MHz) δ10.56 (s, 1H, CONH), 9.76 (s, 1H, Py-NH), 8.84 (s, 1H, OH), 8.13 (d, *J* = 8.4 Hz, 2H, Ar-H), 7.89 (d, *J* = 15.5 Hz, 1H, COCH = C*H*), 7.69 (d, *J* = 15.5 Hz, 1H, COC*H*=CH), 7.59 (d, *J* = 8.4 Hz, 2H, Ar-H), 7.25 (s, 2H, Ar-H), 6.78 (d, *J* = 2.1 Hz, 1H, Ar-H), 6.62 (dd, *J* = 8.9, 7.0 Hz, 2H, Ar-H), 5.45 (s, 1H, Py-CH), 4.06–3.99 (m, 4H, SCH_2_ and CH_3_CH_2_), 3.89 (s, 6H, two *m*-OCH_3_), 3.73 (s, 3H, *p*-OCH_3_), 3.69 (s, 3H, *m*-OCH_3_), 2.23 (s, 3H, Py-CH_3_), 1.15 (t, *J* = 7.3 Hz, 3H, CH_3_CH_2_); ^13^C NMR (DMSO-*d_6_*, *δ* = ppm, 101 MHz) *δ* 187.96, 167.57, 166.77, 153.59, 150.27, 147.66, 146.00, 145.59, 144.37, 143.57, 140.15, 137.12, 132.97, 130.81, 130.31, 121.63, 119.43, 118.86, 115.69, 111.60, 106.94, 99.80, 60.62, 59.56, 59.01, 56.61, 55.95, 40.61, 40.40, 40.19, 39.98, 39.78, 39.57, 39.36, 35.32, 17.95, 14.66; GC-MS m/z calcd for [M]^+^ C_35_H_37_N_3_O_9_S: 675.75, found: 675.91; Anal. Calcd.: C, 62.21; H, 5.52; N, 6.22; Found: C, 62.39; H, 5.64; N, 6.48.

##### Ethyl (*R,E*)-6-methyl-2-((2-oxo-2-((4–(3-(3,4,5-trimethoxyphenyl)acryloyl)phenyl)amino)ethyl)thio)-4–(3,4,5-trimethoxyphenyl)-1,4-dihydropyrimidine-5-carboxylate (CDHPM-10f)

Yield (55%); Light yellow powder; Melting point: 114–115 °C; ^1^H NMR (DMSO-*d_6_*, *δ* = ppm, 400 MHz) *δ* 10.59 (s, 1H, NH amide), 9.86 (s, 1H, Py-NH), 8.12 (d, *J* = 8.2 Hz, 2H, Ar-H), 7.88 (d, *J* = 15.0 Hz, 1H, COCH = C*H*), 7.70–7.64 (m, 3H, Ar-H and COC*H*=CH), 7.23 (s, 2H, Ar-H), 6.49 (s, 2H, Ar-H), 5.51 (s, 1H, Py-CH), 4.15–4.02 (m, 4H, SCH_2_ and CH_3_CH_2_), 3.88 (s, 6H, two *m*-OCH_3_), 3.73 (s, 3H, *p*-OCH_3_), 3.70 (s, 6H, two *m*-OCH_3_), 3.58 (s, 3H, *p*-OCH_3_), 2.23 (s, 3H, Py-CH_3_), 1.18 (t, *J* = 7.2 Hz, 3H, CH_3_CH_2_); ^13^C NMR (DMSO-*d_6_*, *δ* = ppm, 101 MHz) *δ* 187.96, 167.48, 166.75, 153.60, 153.05, 145.95, 144.37, 143.66, 141.45, 140.15, 136.89, 132.95, 130.81, 130.32, 121.61, 118.81, 106.95, 104.22, 99.52, 60.62, 60.38, 59.69, 59.24, 56.62, 56.13, 35.34, 17.98, 14.71; GC-MS m/z calcd for [M]^+^ C_37_H_41_N_3_O_10_S: 719.81, found: 719.80; Anal. Calcd.: C, 61.74; H, 5.74; N, 5.84; Found: C, 61.82; H, 5.91; N, 6.03.

##### Ethyl (*R*)-6-methyl-2-((2-oxo-2-((4-((E)-3–(3,4,5-trimethoxyphenyl)acryloyl)phenyl)amino)ethyl)thio)-4-((*E*)-styryl)-1,4-dihydropyrimidine-5-carboxylate (CDHPM-10g)

Yield (58%); Yellow powder; Melting point: 98.2–98.5 °C; ^1^H NMR (DMSO-*d_6_*, *δ* = ppm, 400 MHz) *δ* 10.67 (s, 1H, CONH), 9.80 (s, 1H, Py-NH), 8.12 (d, *J* = 8.4 Hz, 2H, Ar-H), 7.87 (d, *J* = 15.3 Hz, 1H, COCH = C*H*), 7.78 (d, *J* = 8.3 Hz, 2H, Ar-H), 7.69 (d, *J* = 15.3 Hz, 1H COC*H*=CH), 7.35–7.12 (m, 7H, Ar-H), 6.27 (d, *J* = 15.8 Hz, 1H, PhC*H*=CH), 6.11 (dt, *J* = 16.3, 5.7 Hz, 1H, PhCH = C*H*), 5.13 (d, *J* = 5.5 Hz, 1H, Py-CH), 4.15–4.04 (m, 4H, SCH_2_ and CH_3_CH_2_), 3.88 (s, 6H, two *m*-OCH_3_), 3.73 (s, 3H, *p*-OCH_3_), 2.22 (s, 3H, Py-CH_3_), 1.21 (t, *J* = 7.2 Hz, 3H, CH_3_CH_2_); ^13^C NMR (DMSO-*d_6_*, *δ* = ppm, 101 MHz) *δ* 187.91, 167.61, 166.37, 153.60, 153.57, 151.02, 146.73, 144.34, 143.72, 140.16, 137.14, 132.97, 130.81, 130.62, 130.37, 128.91, 128.32, 126.64, 121.59, 118.94, 106.94, 97.79, 60.62, 59.65, 57.09, 56.61, 56.58, 35.55, 17.97, 14.75; GC-MS m/z calcd for [M]^+^ C_36_H_37_N_3_O_7_S: 655.24, found: 655.38; Anal. Calcd.: C, 65.94; H, 5.69; N, 6.41; Found: C, 66.13; H, 5.85; N, 6.59.

### Biological activity

#### NCI-60 cells screening

The herein designed compounds **CDHPM-10a-g** were screened at NCI-DTP (MD, USA) on a full panel of 60 tumour cell lines comprising 9 different tumour types: Breast, Prostate, Renal, Ovarian, Melanoma, CNS, Colon, Leukaemia, and NSCL cancers. All the target analogs were initially tested at 10 µM (single dose). All compounds represented by scaffold “B” expressed sufficient potency in one-dose assay so that they were examined further at five-dose mode (0.01–100 µM) following the SRB standard procedure of the Drug Evaluation Branch^62^^,^^78^. In the Supplementary Material, the experimental methods were specified.

#### SRB cytotoxicity assay

The assay was applied following the procedure described elsewhere^65^. MCF-7/HSF cell lines were purchased from Nawah Scientific Company, Egypt. Briefly, in a 96-well plate, MCF-7/HSF cells (100 μL of 5 × 10^3^ cells/well) were seeded and incubated for 24 h. Various concentrations of the examined analogs (100 μL) were added and incubated for 72 h. Plates were then fixed with 150 μL of 10% TCA and incubated at 4 °C for 1 h. Cells were washed five times with deionised water. After that, 70 μL of SRB solution (0.4% w/v) was added and incubated in the dark for 10 min at RT. Cells were washed thrice with acetic acid (1%) and air-dried overnight. Finally, the protein-bound SRB stain was dissolved by adding 150 μL TRIS (10 mM), and BMG LABTECH^®^-FLUOstar Omega microplate reader (Ortenberg, Germany) was used to measure the absorbance at 540 nm. The selectivity index (SI) was calculated from the following equation. The test compound and NC were examined in triplicate, and the results were denoted as ±*SD*.

$$\text{SI}=\frac{{\text{IC}}_{50}\text{ HSF calls}}{{\text{IC}}_{50}\text{ of cancer cells}}$$

#### MTT cytotoxicity assay

The assay was performed as described earlier^20^^,^^79–81^. Human umbilical vein endothelial cell line (HUVECs) was purchased from Vacsera, Egypt, of ATCC origin. Briefly, in a 96-well plate, HUVECs were seeded (10^6^ cells/cm^2^) and incubated overnight at 37 °C and CO_2_ (5%). The cells were then incubated for 48 h with serial dilutions of C-2, CDHPM-10a-g utilising SF as a reference drug. After 4 h incubation with MTT (Cat. No. M-5655) 5 mg/mL, anhydrous isopropanol containing 10% Triton X-100 plus 0.1 N HCl was added. The absorbance was read using a Bioline plate reader at λ_570- 630 _nm.

#### Cell cycle analysis

The assay was conducted following the manufacturer’s instructions^20^^,^^66^^,^^67^^,^^79^^,^^82^. Briefly, MCF-7 cells (10^5^ cells/mL) were treated with either test compound **CDHPM-10e** (IC_50_) or DMSO (NC) for 48 h. Then, MCF-7 cells were trypsinized, washed with PBS (ice-cold; pH 7.4), fixed with ice-cold ethanol (60%; 2 ml), and incubated for 1 h at 4 °C. The fixed cells were rewashed with PBS, stained with 50 µg/mL RNAase A and 10 µg/mL propidium iodide (PI), and incubated in the dark for 20 min at 37 °C. They were analysed utilising FL2 (λex/em 535/617 nm; 12 000 events) signal detector (ACEA Novocyte™ flow cytometer, ACEA Biosciences Inc., San Diego, CA, USA) for DNA contents. Cell cycle analysis was calculated utilising ACEA NovoExpress™ software (ACEA Biosciences Inc., San Diego, CA, USA). The test compound and NC were examined in triplicate, and the data were denoted as ±*SD*.

#### Apoptosis analysis

The MCF-7 cells (10^5^ cells/mL) were incubated with either 2.52 µM of **CDHPM-10e** or NC for 48 h. After that, cells were trypsinized, washed two times with PBS (ice-cold; pH 7.4), and treated with 0.5 ml Annexin V-FITC/PI solution in the dark at RT for 0.5 h. Cells were then injected *via* ACEA Novocyte™ flow cytometer (ACEA Biosciences Inc., San. Diego, CA, USA) and analysed fluorescent signals for FITC and PI utilising FL1 and FL2. Signal detector, correspondingly (*λ*_ex/em_ 488/530 nm for FITC and *λ*_ex/em_ 535/617 nm for PI). Twelve thousand events are acquired, and positive FITC and PI cells are quantified by quadrant analysis and calculated utilising ACEA NovoExpress™ software (ACEA Biosciences Inc., San Diego, CA, USA). The test compound and NC were examined in triplicate, and the data were denoted as ±*SD*.

#### VEGFR-2 inhibitory activity

The assay was conducted following the BPS Bioscience^®^ procedure^17^^,^^83–86^. Briefly, Kinase Buffer 1 (5×), ATP, and PTK substrate (50×) of KDR (Cat. No. 40325) were thawed, and 25 µl/well master mixture was prepared, added, and then the test compound **CDHPM-10e** was added using sorafenib a reference drug. To the Blank wells, Kinase Buffer 1 (1×) was added. VEGFR-2 enzyme in Kinase Buffer 1 (1×) was added to sorafenib and test compound wells after incubation for 45 min. At 30 °C, ADP-Glo reagent/Kinase-Glo Max reagent was added, and the plate was incubated at RT for 0.25 h. in a dark place. Tecan–spark^®^ reader was used to measure the luminescence immediately. From all readings, the “Blank” was subtracted. The test compound and sorafenib were examined in duplicate, and IC_50_ values were denoted as ±*SD*.

#### Western blotting

MCF-7 cell line (2.5*10^5^ cells/mL) was seeded and treated as described above. Cells were then lysed using RIPA buffer containing 1x protease and phosphatase inhibitor cocktail (Thermo Scientific™) after 48 h of incubation with hybrid **CDHPM-10e**. After centrifugation at 4 °C, the supernatant was collected. The Pierce BCA Protein Assay Kit (Thermo Scientific™, 23225) was utilised for protein concentration detection. Cas-3 and p53 were separated using SDS-PAGE for 45 min at 100 V. Then, they transferred to a nitrocellulose membrane, which was incubated with an appropriate primary antibody for 12 h. After that, an HRP-conjugated secondary antibody was added. The loading control B-actin was used. The membrane was then developed utilising WesternBright^®^ ECL (K-12045-D20), and the signals were recorded by ChemiDoc XRS^+^ (1708265, Bio-Rad). A CCD camera-based imager was used to capture the chemiluminescent signals. The band intensities were analysed using the image analysis software^20^^,^^87^. The antibodies were purchased from Cell Signalling Technology and Invitrogen, as depicted in Table 9.

**Table 9.** Antibodies used in the assay.

Antibody	Catalog No.	Company	Dilution used
Caspase-3	9662S	Cell signalling	1:1000
P53	PA5-27822	Invitrogen	1:1000
B-actin	MA5-11869	Invitrogen	1:1000

#### Cell migration assay

MCF-7 cell line (2 × 10^5^/well) was seeded in a coated 12-well plate for scratch cell migration assay and incubated in 5% FBS-DMEM for 12 h at 5% CO_2_ and 37 °C. Then, horizontal scratches were applied to the confluent monolayer. PBS was used to wash the plate thoroughly. Test wells were treated with fresh medium containing **CDHPM-10e,** while the control wells were replenished with fresh media. Images were captured utilising an inverted microscope at specified time intervals. The plate was then incubated at various intervals at 5% CO_2_ and 37 °C. MII ImageView software version 3.7 was used to analyse the captured images.

### In silico *molecular modelling studies*

The state-of-the-art OpenEye^®^ scientific software (2023) was utilised for *in silico* molecular modelling evaluation. The software comprises EON^24^, OMEGA^88^, Spruce^89^, FRED^90^, Docking-Report^91^, and VIDA^71^ modules. Initially, a library of the designed hybrids **CDHPM-10a-g** and SOR was energy minimised using a force field of MMFF94. SOR was used as a query ligand for the designed library in EON scaffold hoping. On the other hand, the docking scheme began with multi-conformers using the OMEGA^®^ application. Then, the Spruce^®^ application was used for VEGFR-2 (PDB ID: 3WZE) receptor preparation. After that, the FRED Chemgauss4 was generated utilising the FRED^®^ application. The Docking-Report was obtained from OpenEye’s Docking-Report application. Finally, visualisation of the examined compounds’ binding interaction towards the VEGFR-2 binding pocket was accomplished by the Vida^®^ module. Moreover, the 2D diagram of the compound’s pose was carried out by the Discovery Studio Visualisation application (DS).

### ADMET prediction and drug-likeness properties

The efficient ADMETlab 2.0, a web-based tool, was utilised to analyse ADMET and drug-likeness parameters for the most potent analog, **CDHPM-10e**^74^^,^^75^.

## Conclusions

In this study, a novel series of furoxan-based thiazolidine-2,4-diones and 1,4-dihydropyrimidines tethered to 3,4,5-trimethoxyxhalcone through *S*-acetamide bridge have been designed and synthesised. The target analogs were evaluated for their anticancer activity towards a full panel of 60 tumour cells. Among the tested hybrids, **CDHPM-10a-g** revealed mean %inhibitions range from 76.40 to 147.69%. Superiorly, compounds **CDHPM-10e** and **CDHPM-10f** exhibited the highest MGI% of 147.69 and 140.24%, respectively. Analogs **CDHPM-10a, CDHPM-10b,** and **CDHPM-10d-f** displayed higher mean %inhibitory activity than the reference drug sorafenib (MGI% = 105.46%). Five-dose testing also exposed that compound **CDHPM-10e** with hydrophilic moiety showed the highest potencies over all the herein examined 60 tumour cells with single-digit micromolar GI_50_ scale and mean GI_50_ value of 1.83 µM. Notably, the hybrid **CDHPM-10e** exhibited potent cytostatic effects at a single-digit micromolar range (TGI spanning in the interval: 2.43–9.81 µM) towards 58 tumour cells. All the target analogs were found to be non-lethal towards Leukaemia (CCRF-CEM, HL-60(TB), K-562, MOLT-4, RPMI-8226 and SR), Ovarian (NCI/ADR-RES and OVCAR-4), Breast (HS 578 T and T-47D) and Prostate (PC-3) cancer cells with LC_50_ > 100 µM. Among the target compounds, **CDHPM-10e** displayed LC_50_ values spanning in the interval: 2.04–70.60 µM. The designed compounds **CDHPM-10a-g** were exposed as potent non-selective broad-spectrum anticancer agents over all NCI subpanels with an SI range of 0.66–1.97. Also, compound **CDHPM-10e** revealed potency towards KDR comparable to that of sorafenib with a sub-micromolar IC_50_ of 0.112 µM. Furthermore, **CDHPM-10e** was more potent than sorafenib, with IC_50s_ of 2.52 and 5.10 µM towards the MCF-7 cell line. The data showed that **CDHPM-10e** could effectively enhance Sub-G1-stage arrest and prompt apoptosis *via* caspase and p53-dependent mechanisms. Compound **CDHPM-10e** revealed significant anti-metastatic activity as detected by wound healing assay. The modelling study implies that **CDHPM-10e** overlaid well with sorafenib and formed a strong H-bond in the DFG binding domain. The ADMET and Drug-likeness study exposed that compound **CDHPM-10e** met the criteria of Pfizer. Therefore, the presented novel potent anti-cancer agent merits further devotion in developing VEGFR-2 inhibitors.

## Supplementary Material

Supplemental Material

## Data Availability

Additional data may be requested from the authors.
