# Supplementary material for: Novel NO-TZDs and trimethoxychalcone-based DHPMs: design, synthesis, and biological evaluation as potential VEGFR-2 inhibitors
Source: J Enzyme Inhib Med Chem. 2024 Jun 21;39(1):2358934. doi: 10.1080/14756366.2024.2358934 (PMC467104; doi:10.1080/14756366.2024.2358934)
Supplement: Supplemental Material [file IENZ_A_2358934_SM8485.pdf]

Supporting Information

Novel NO-TZDs and Trimethoxychalcone-Based DHPMs: Design, Synthesis and Biological Evaluation as Potential VEGFR-2 Inhibitors

Mater Mahnashi [a], Mohammed H Nahari [b], Hassan Hussain Almasoudi [b], Abdulaziz Hassan Alhasaniah [b], Sara M. Elgazwi [c] and Mahrous A. Abou-Salim [d,*]

[a] Department of Pharmaceutical Chemistry, College of Pharmacy, Najran University, Najran, 1988, Saudi Arabia; [b] Department of Clinical Laboratory Sciences, College of Applied Medical Sciences, Najran University, 1988, Najran, Saudi Arabia; [c] Department of Chemistry, University of Derna, Derna, Libya; [d] Pharmaceutical Organic Chemistry, Faculty of Pharmacy, Al-Azhar University, Assiut 71524, Egypt

*Mahrous A. Abou-Salim, mahrousabousalim@azhar.edu.eg, Pharmaceutical Organic Chemistry, Faculty of Pharmacy, Al-Azhar University, P.O. Box 71524, Assiut, Egypt.

Contents

1 Spectral data 1

2 NCI-60 cell assay results 16

3 Docking report of compound CDHPM-10e 38

4 NCI-60 screening methodology 39

5 References 40

1 Spectral data

1.1 NO-TZD-3a

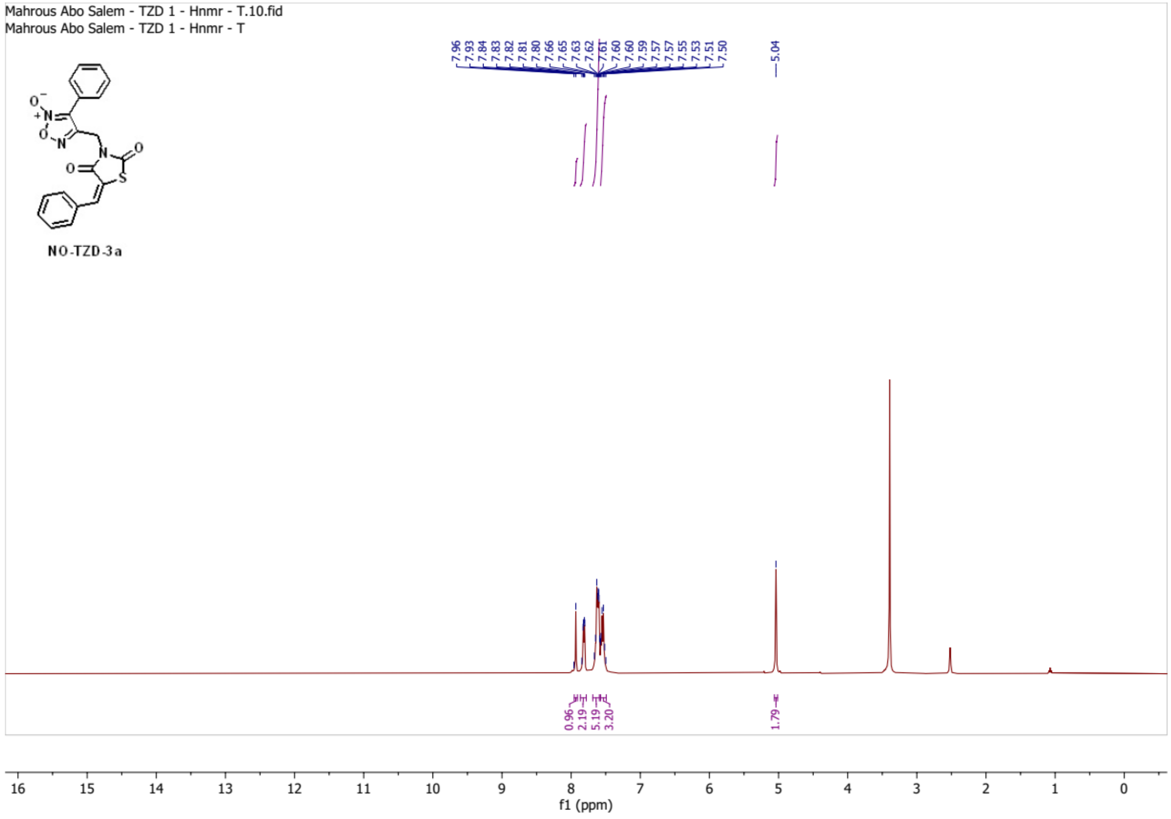

Figure S 1. ^{1}H NMR (400 MHz, DMSO-d_6) of **NO-TZD-3a**.

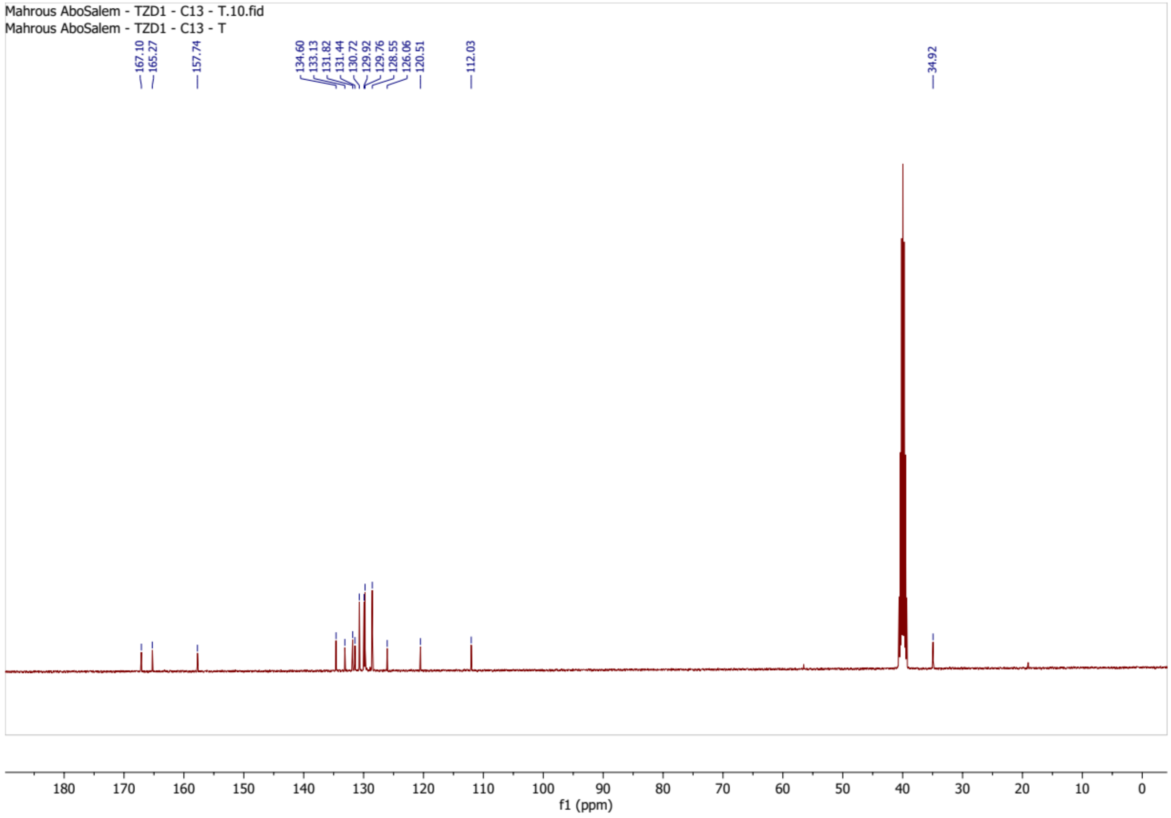

Figure S 2. ^{13}C NMR (101 MHz, DMSO-d_6) of **NO-TZD-3a.**

1.2 NO-TZD-3b

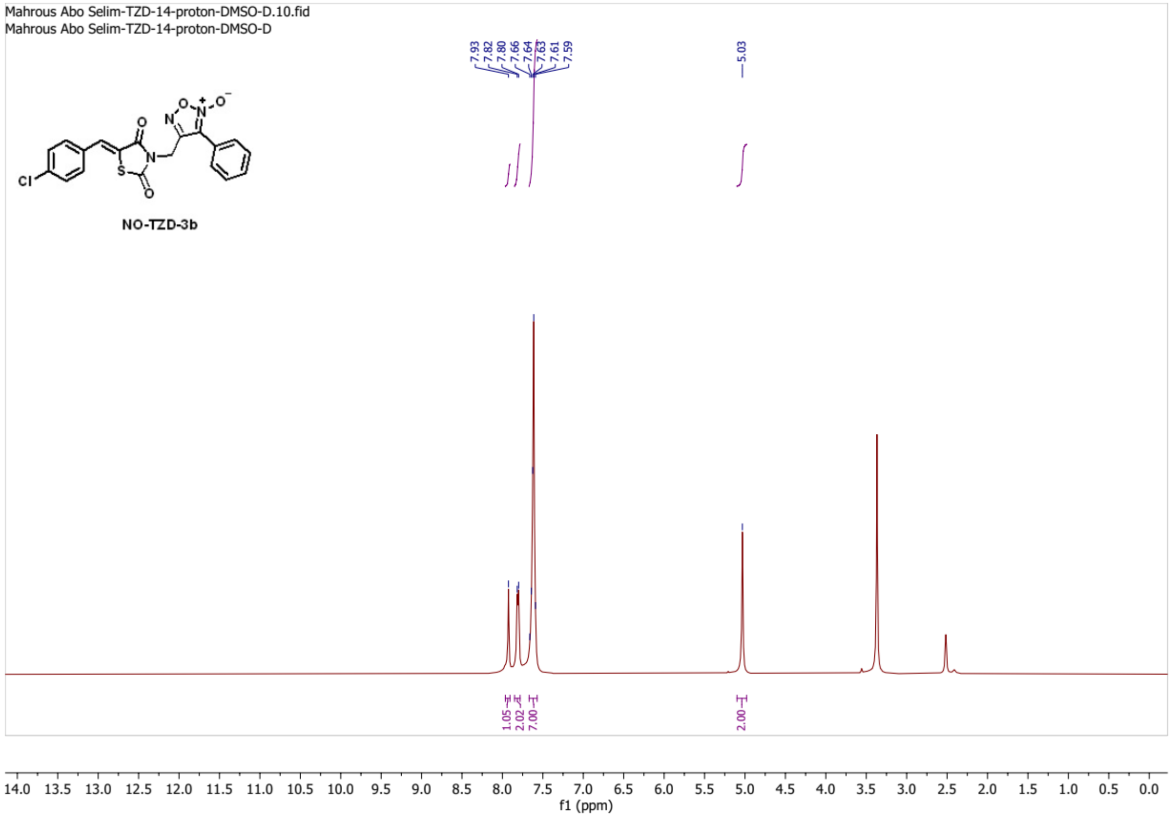

Figure S 3. ^{1}H NMR (400 MHz, DMSO-d_6) of **NO-TZD-3b.**

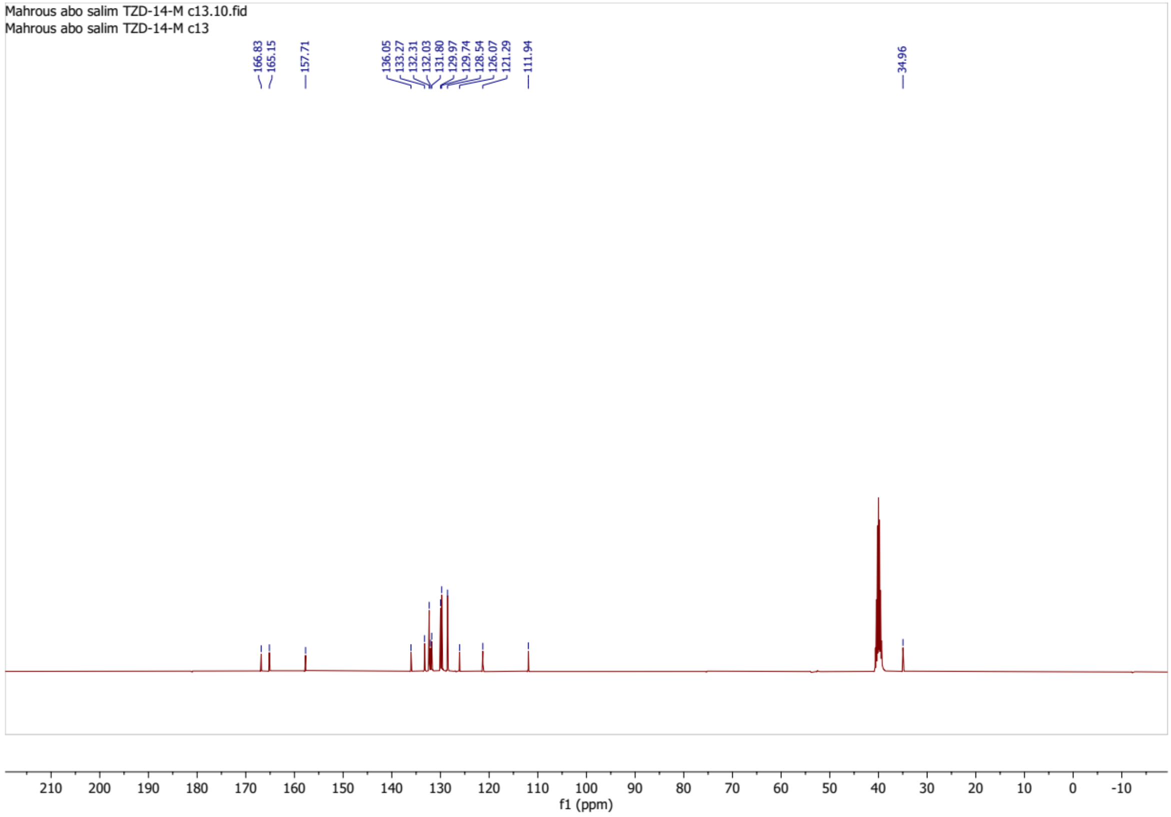

Figure S 4. ^{13}C NMR (101 MHz, DMSO-d_6) of **NO-TZD-3b.**

1.3 NO-TZD-3c

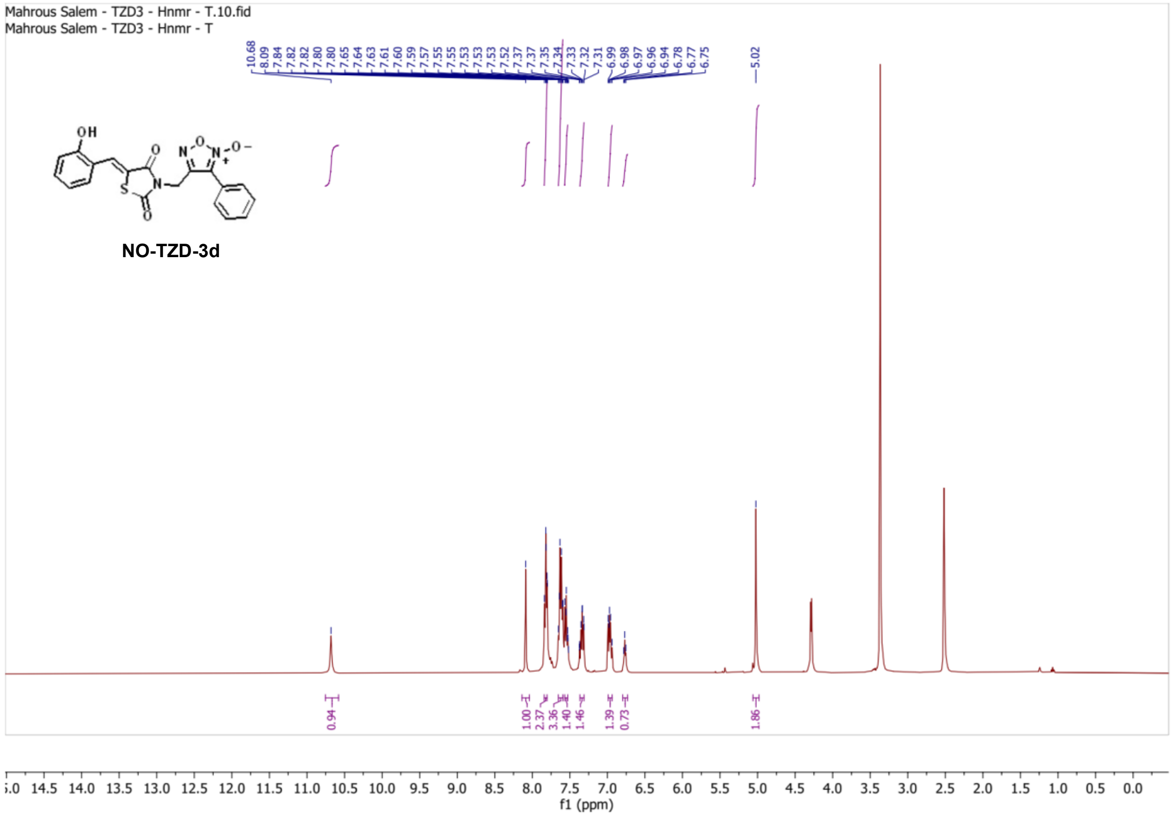

Figure S 5. ^{1}H NMR (400 MHz, DMSO-d_6) of **NO-TZD-3c.**

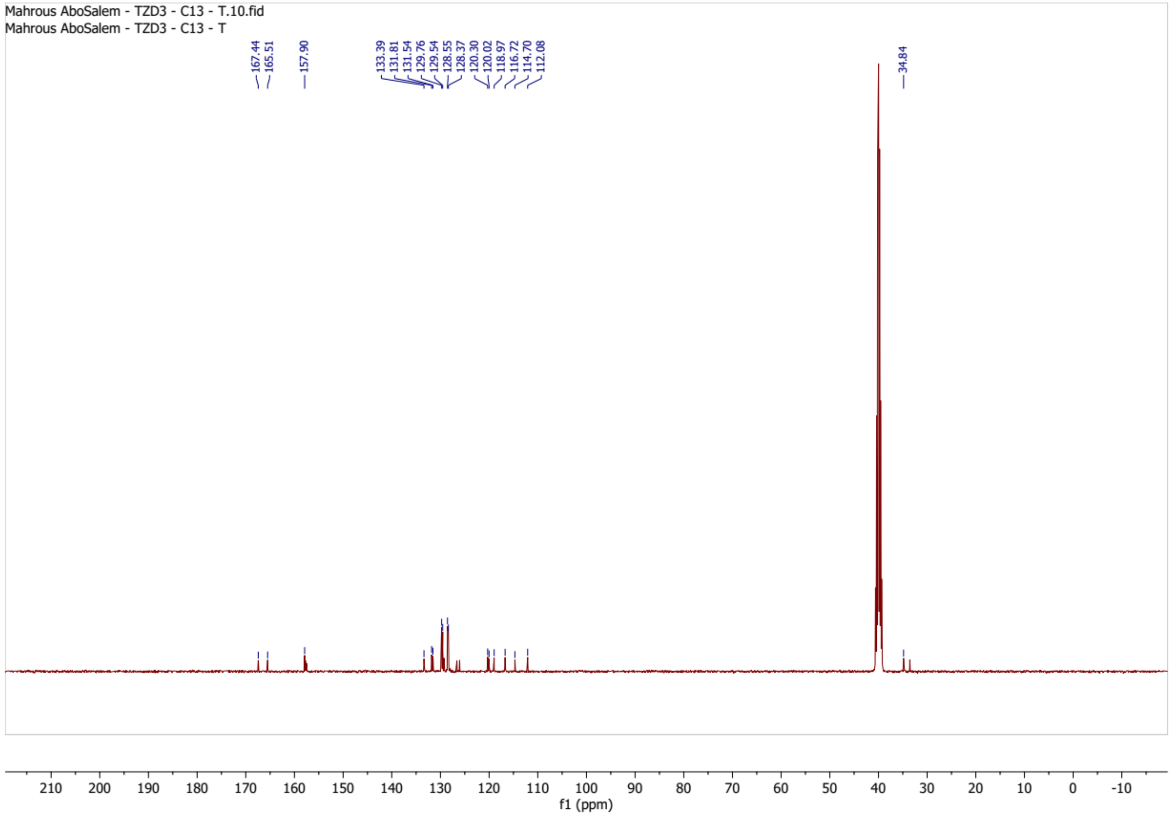

Figure S 6. ^{13}C NMR (101 MHz, DMSO-d_6) of **NO-TZD-3c.**

1.4 NO-TZD-3d

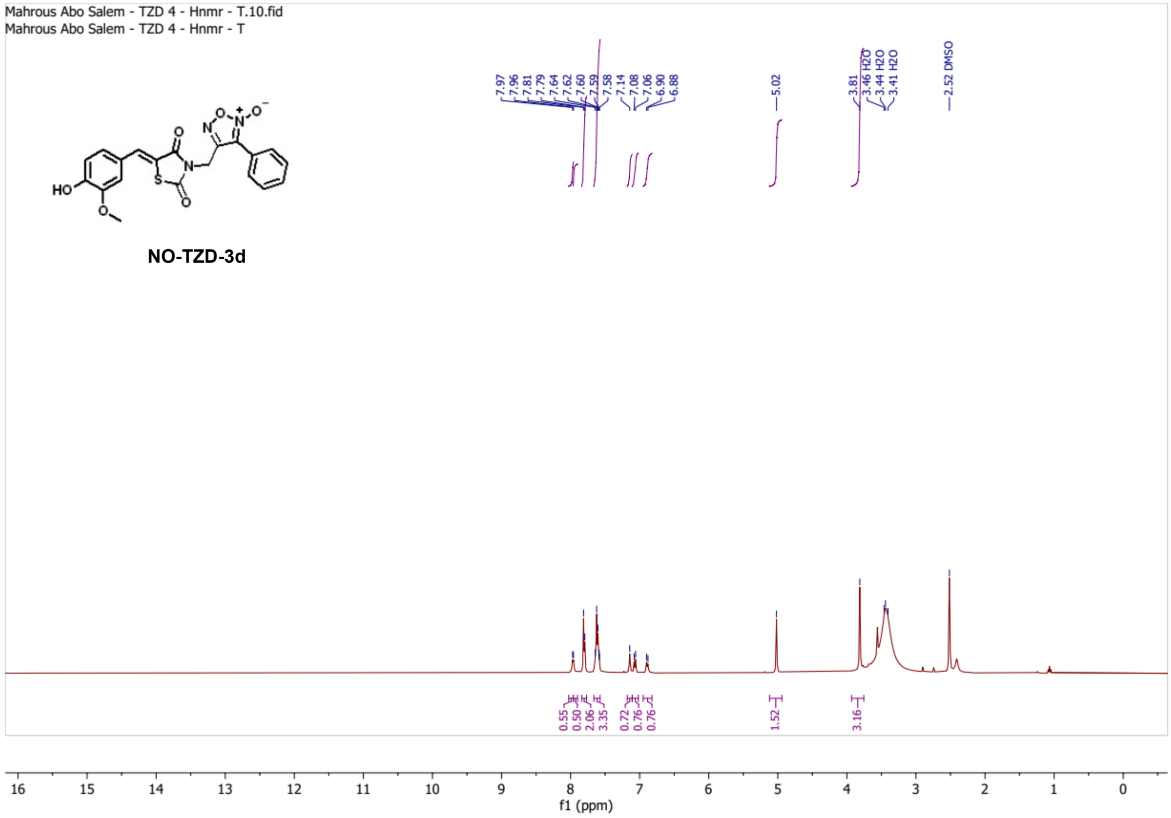

Figure S 7. ^{1}H NMR (400 MHz, DMSO-d_6) of **NO-TZD-3d.**

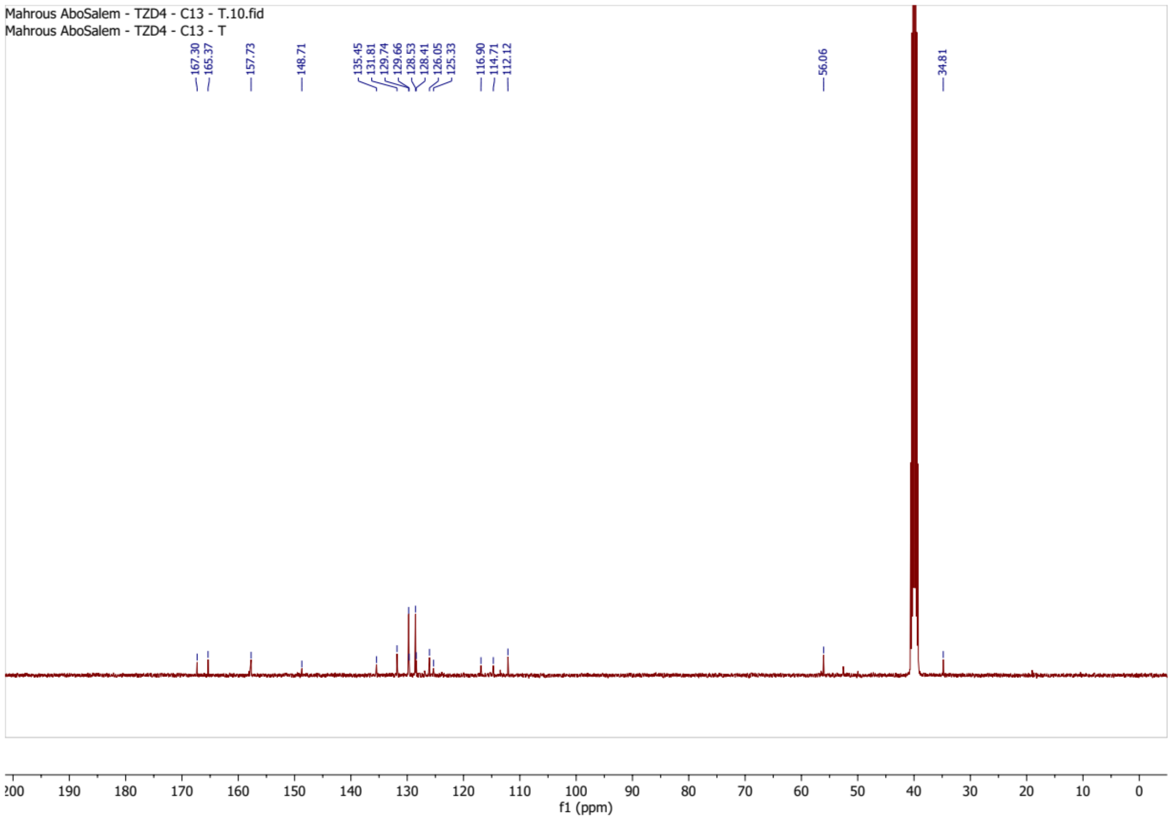

Figure S 8. ^{13}C NMR (101 MHz, DMSO-d_6) of **NO-TZD-3d.**

1.5 NO-TZD-5

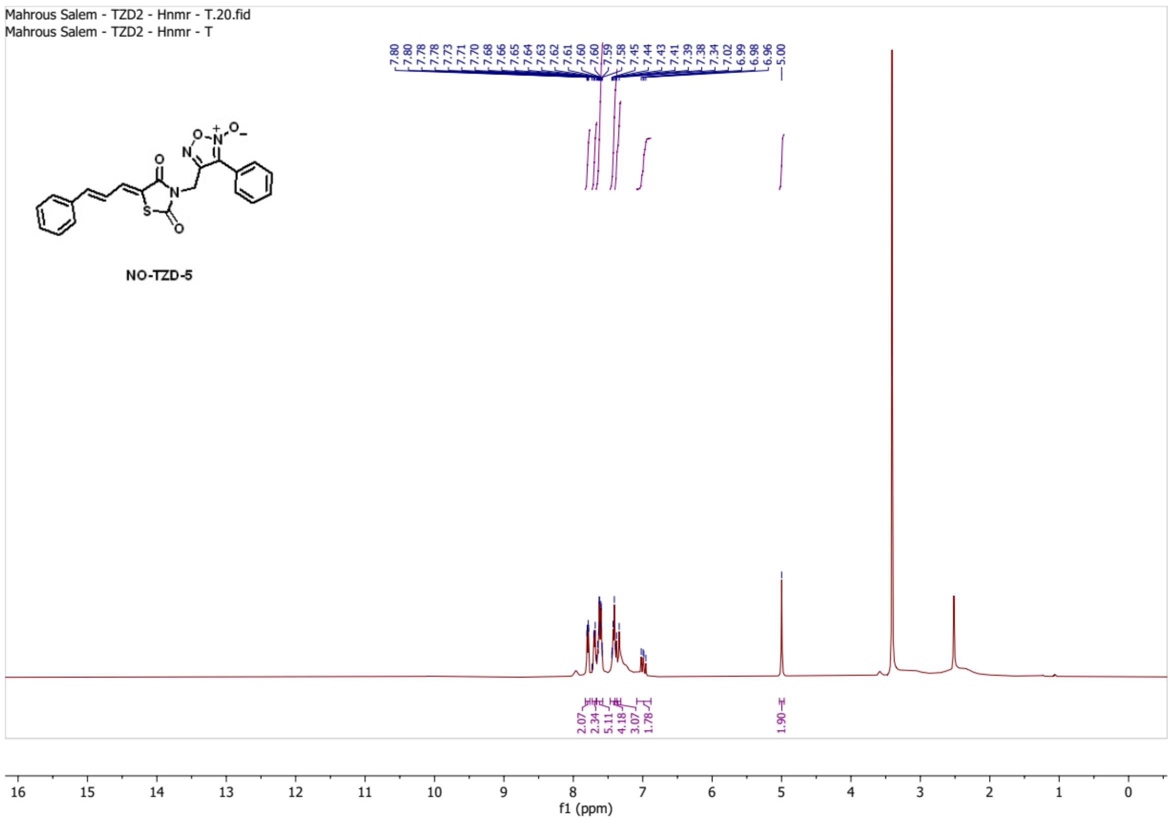

Figure S 9. ^{1}H NMR (400 MHz, DMSO-d_6) of **NO-TZD-5.**

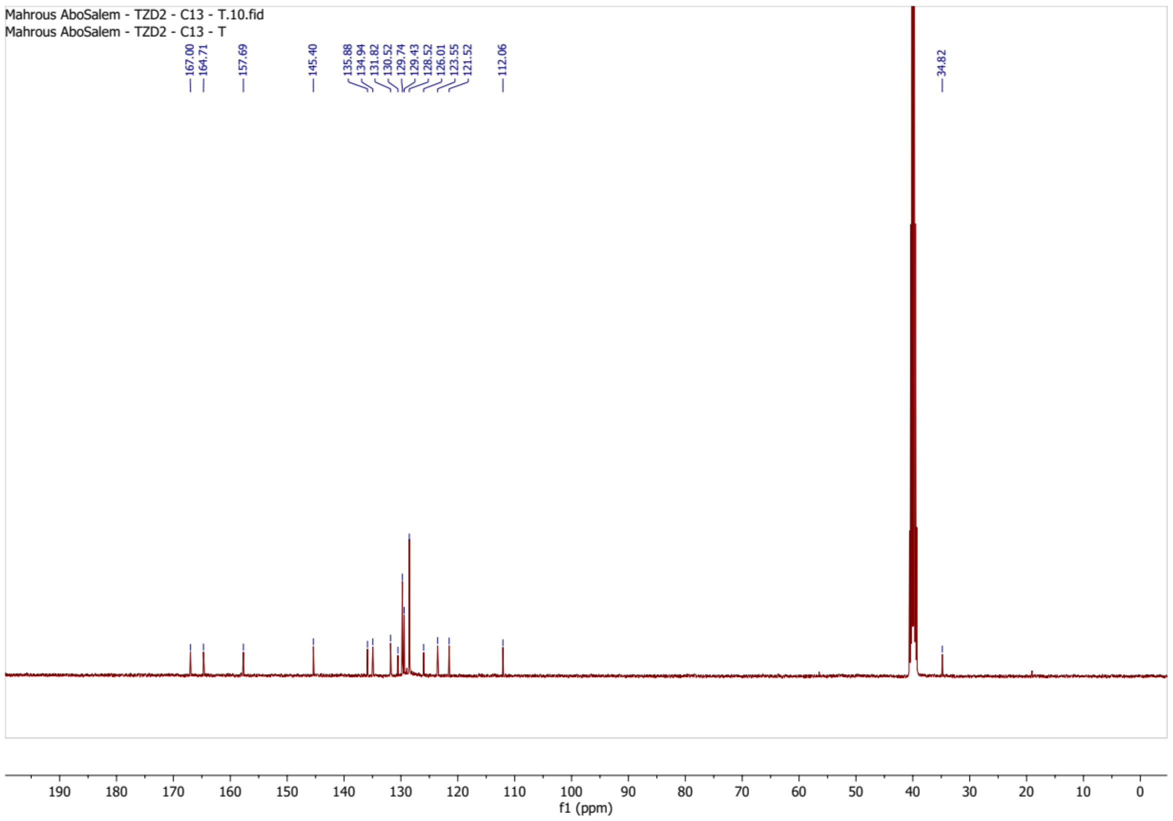

Figure S 10. ^{13}C NMR (101 MHz, DMSO-d_6) of **NO-TZD-5.**

1.6 NO-TZD-6

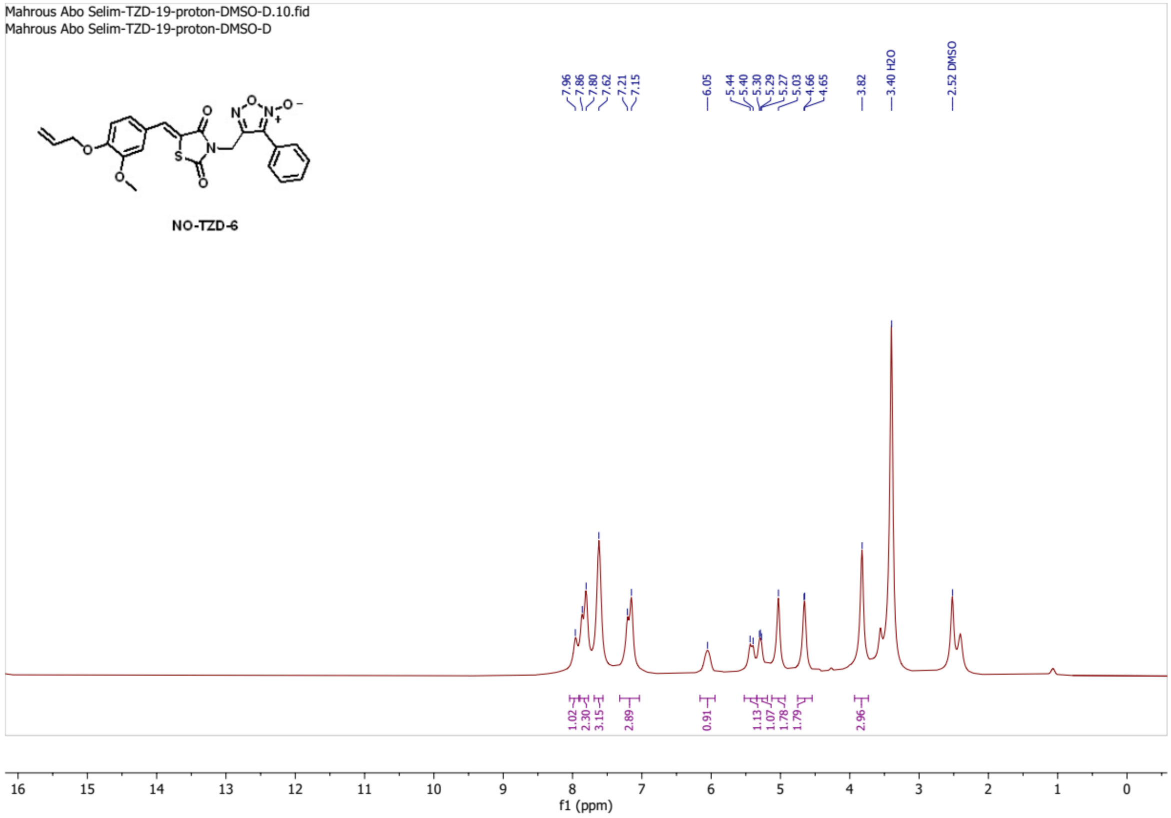

Figure S 11. ^{1}H NMR (400 MHz, DMSO-d_6) of **NO-TZD-6.**

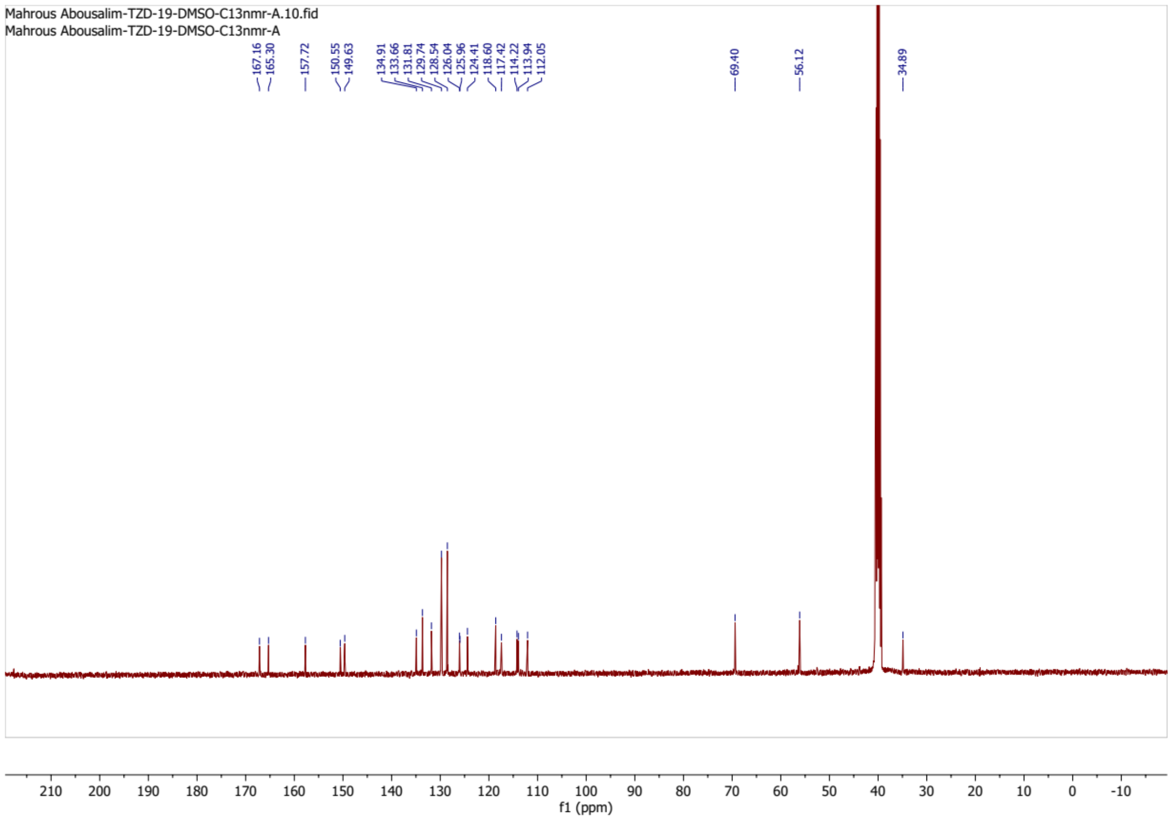

Figure S 12. ^{13}C NMR (101 MHz, DMSO-d_6) of **NO-TZD-6.**

1.7 DHPM-7a

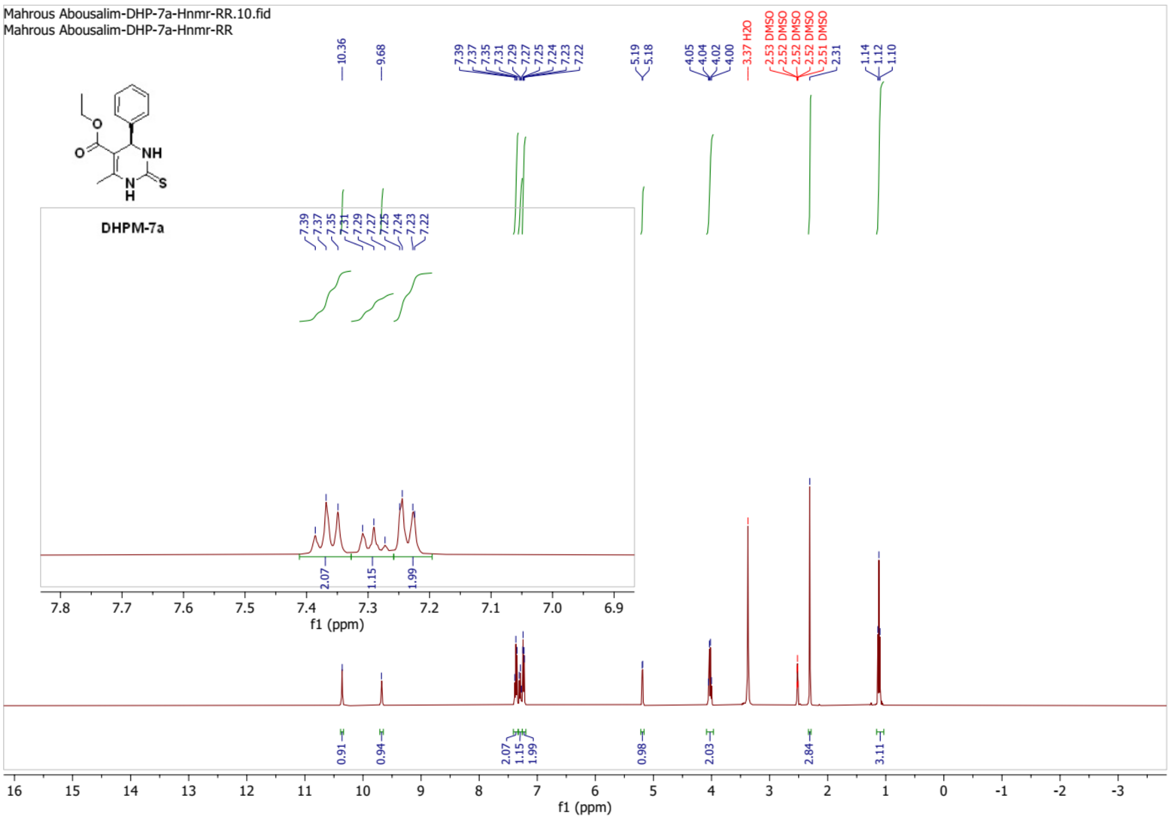

Figure S 13. ^{1}H NMR (400 MHz, DMSO-d_6) of **DHPM-7a**.

1.8 Methyl ester analog of DHPM-7a

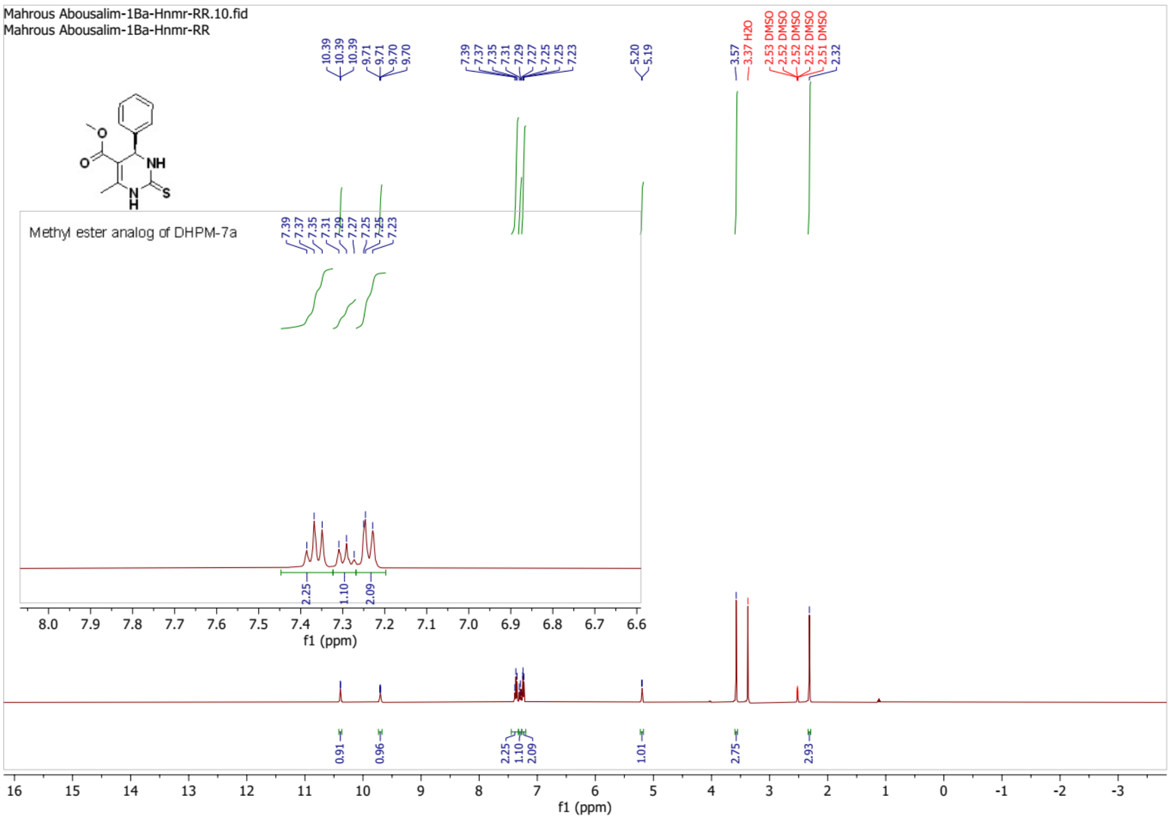

Figure S 14. ^{1}H NMR (400 MHz, DMSO-d_6) of methyl ester analog of **DHPM-7a**.

1.9 *CDHPM-10a*

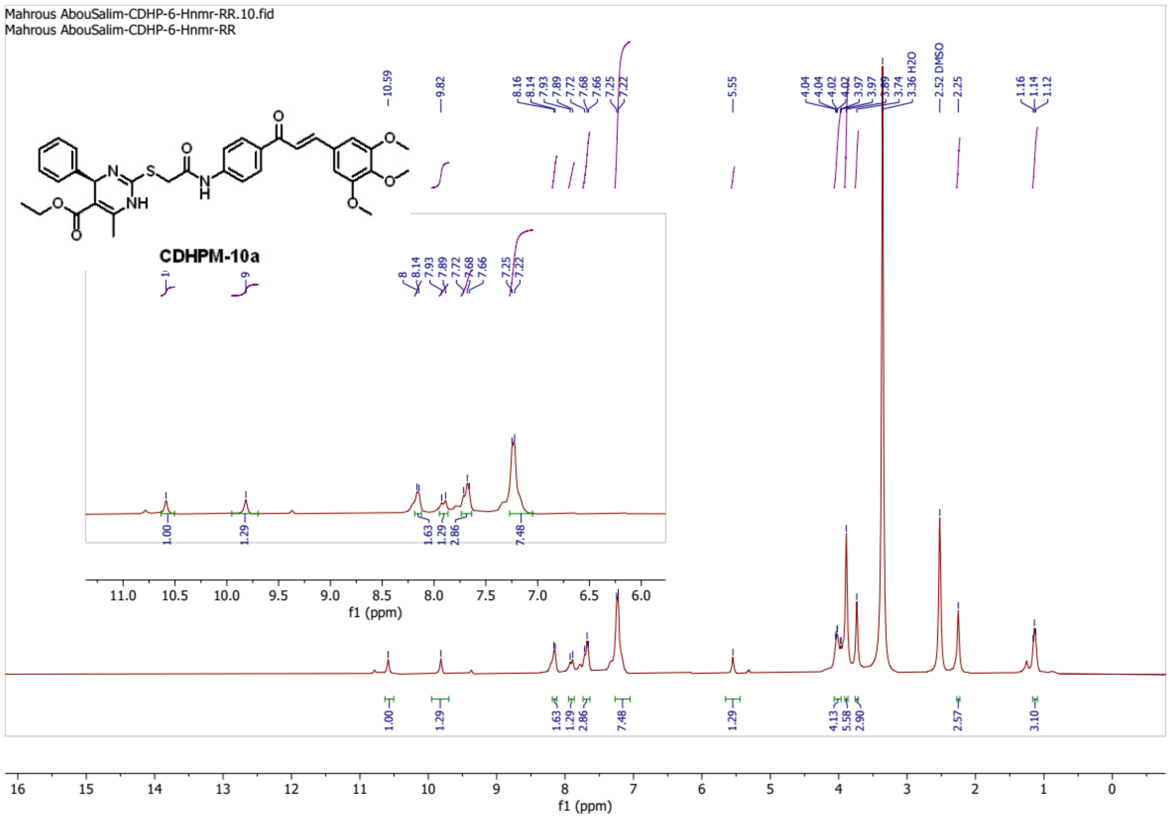

Figure S 15. ^{1}H NMR (400 MHz, DMSO-d_6) of **CDHPM-10a.**

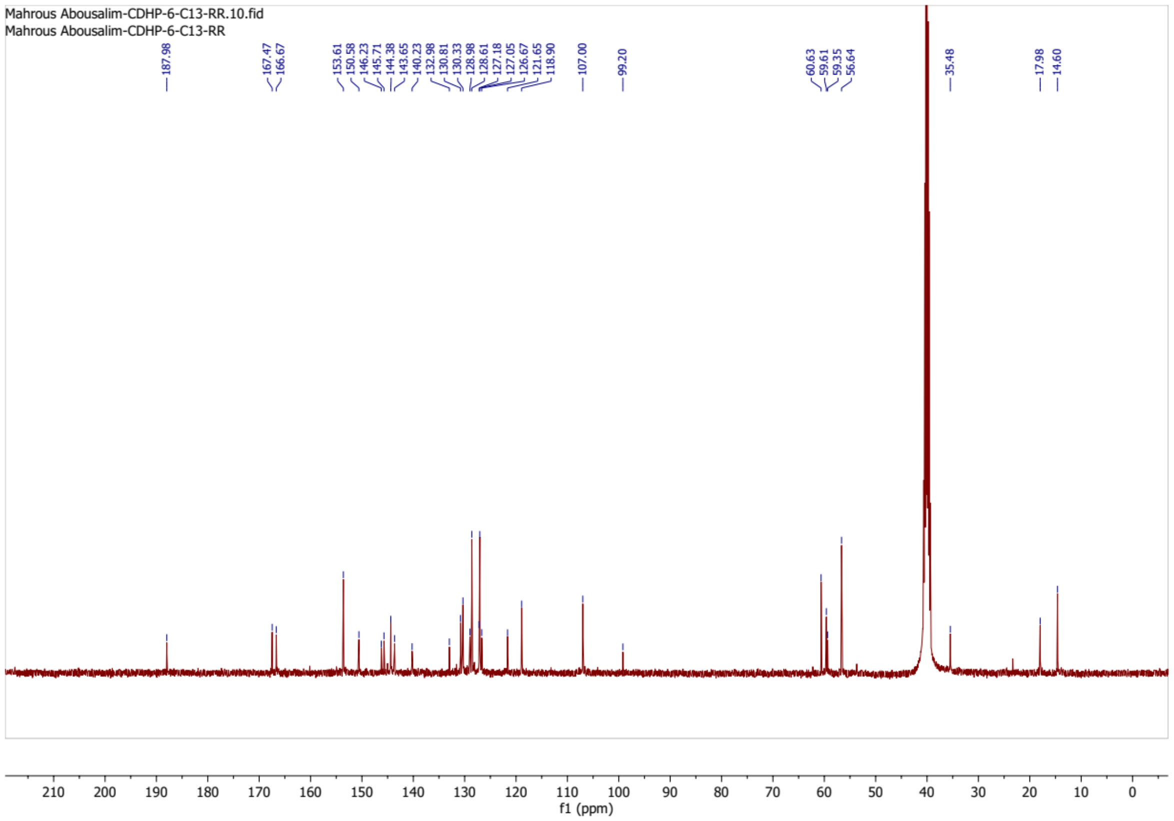

Figure S 16. ^{13}C NMR (101 MHz, DMSO-d_6) of **CDHPM-10a.**

1.10 *CDHPM-10b*

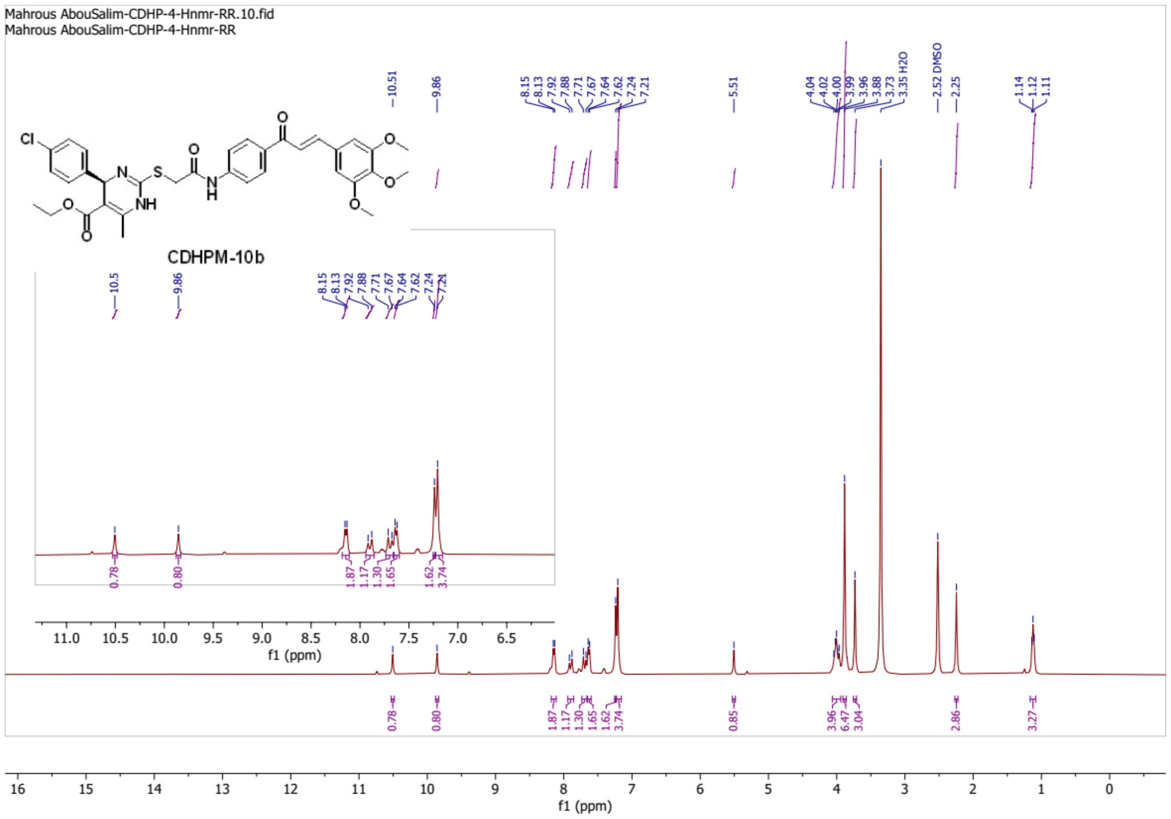

Figure S 17. ^{1}H NMR (400 MHz, DMSO-d_6) of **CDHPM-10b.**

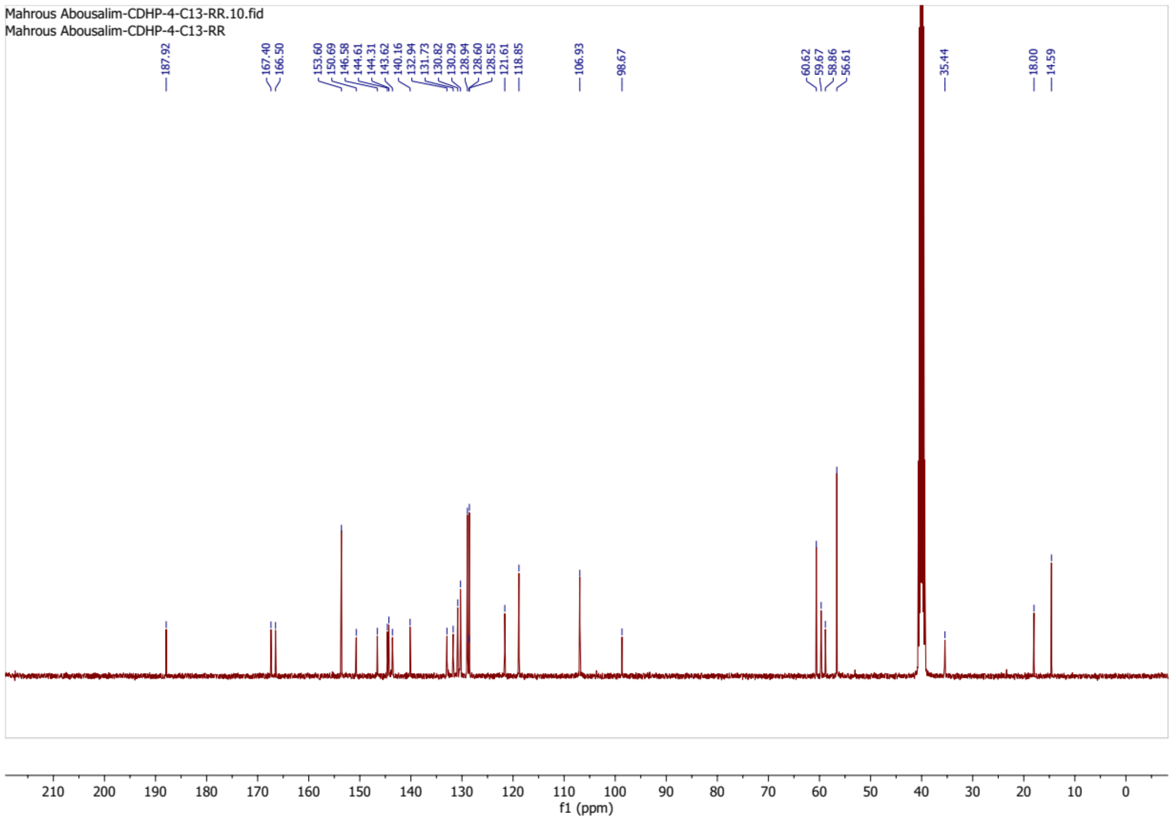

Figure S 18. ^{13}C NMR (101 MHz, DMSO-d_6) of **CDHPM-10b.**

1.11 *CDHPM-10c*

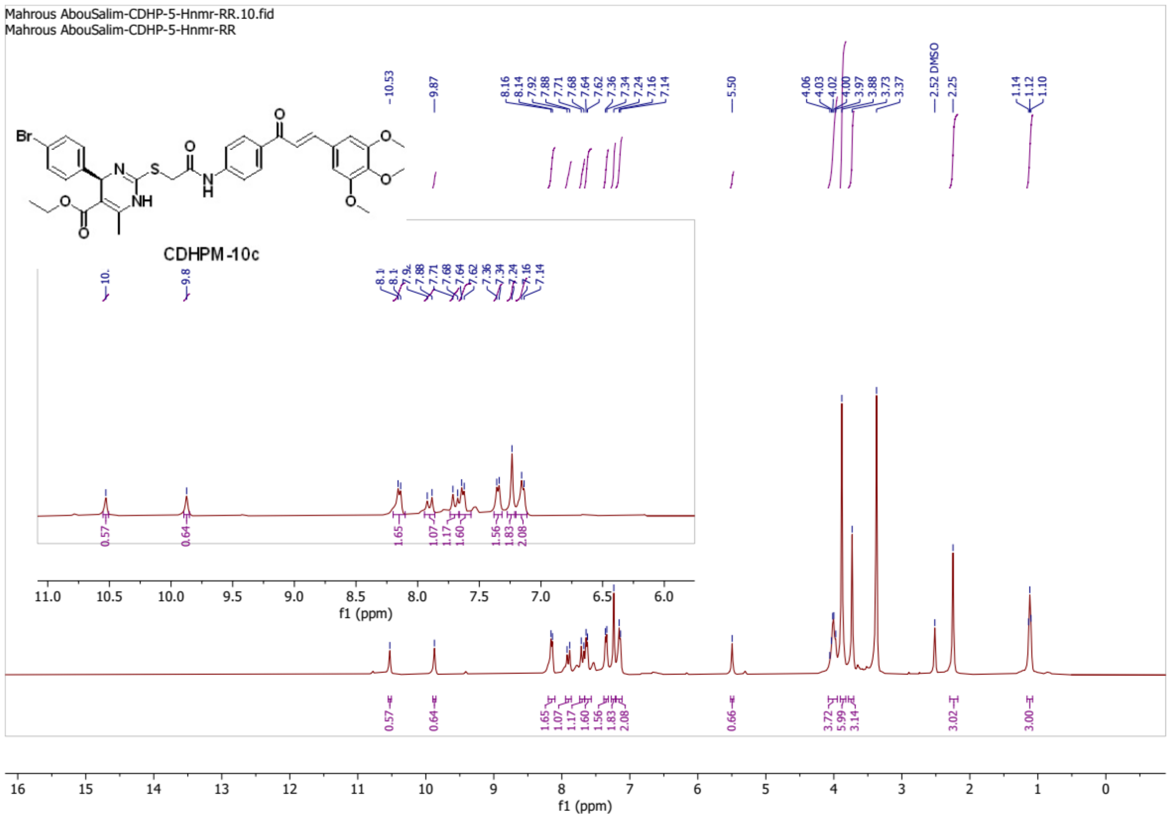

Figure S 19. ^{1}H NMR (400 MHz, DMSO-d_6) of **CDHPM-10c.**

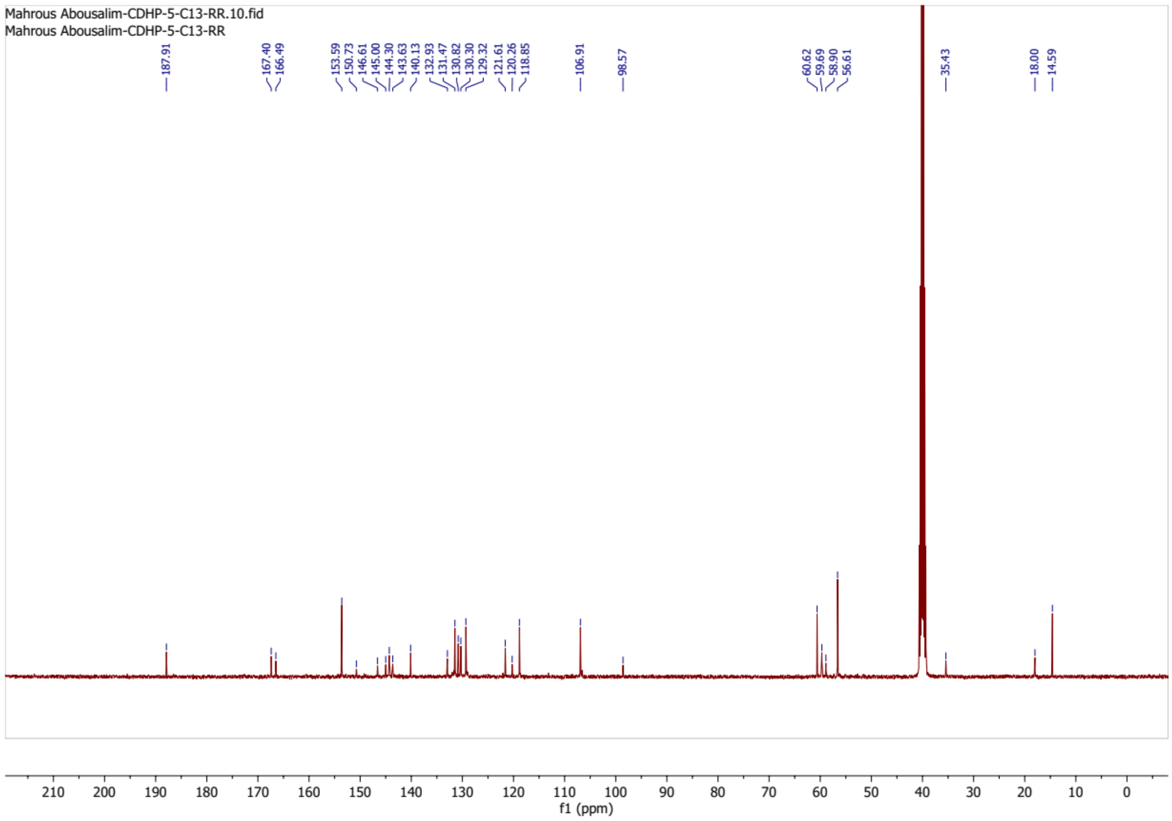

Figure S 20. ^{13}C NMR (101 MHz, DMSO-d_6) of **CDHPM-10c.**

1.12 *CDHPM-10d*

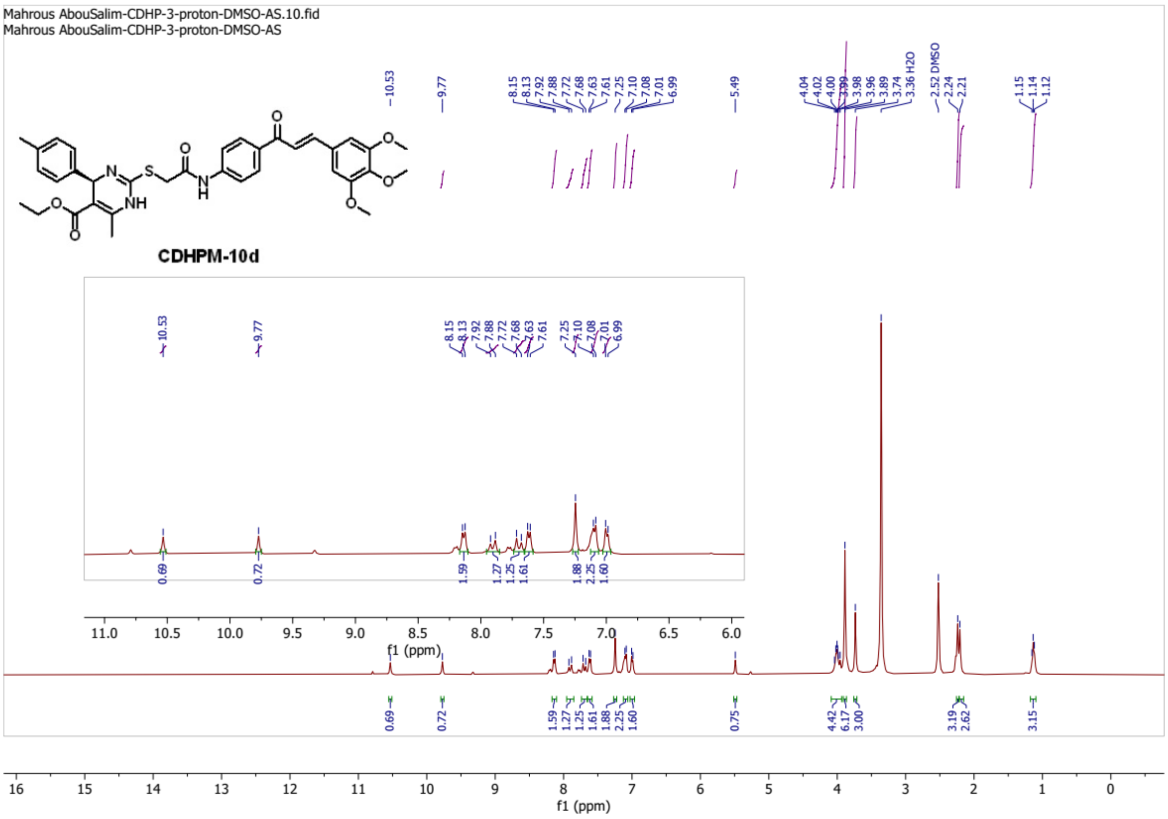

Figure S 21. ^{1}H NMR (400 MHz, DMSO-d_6) of **CDHPM-10d.**

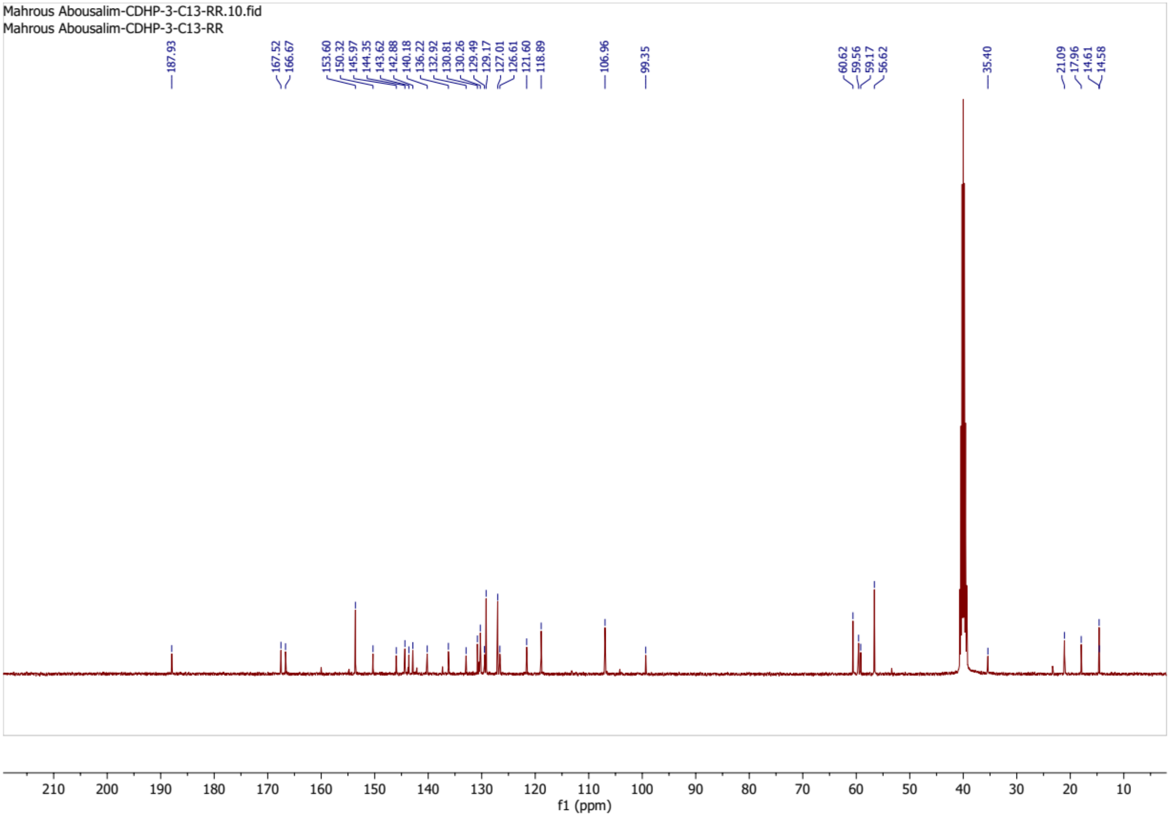

Figure S 22. ^{13}C NMR (101 MHz, DMSO-d_6) of **CDHPM-10d.**

1.13 CDHPM-10e

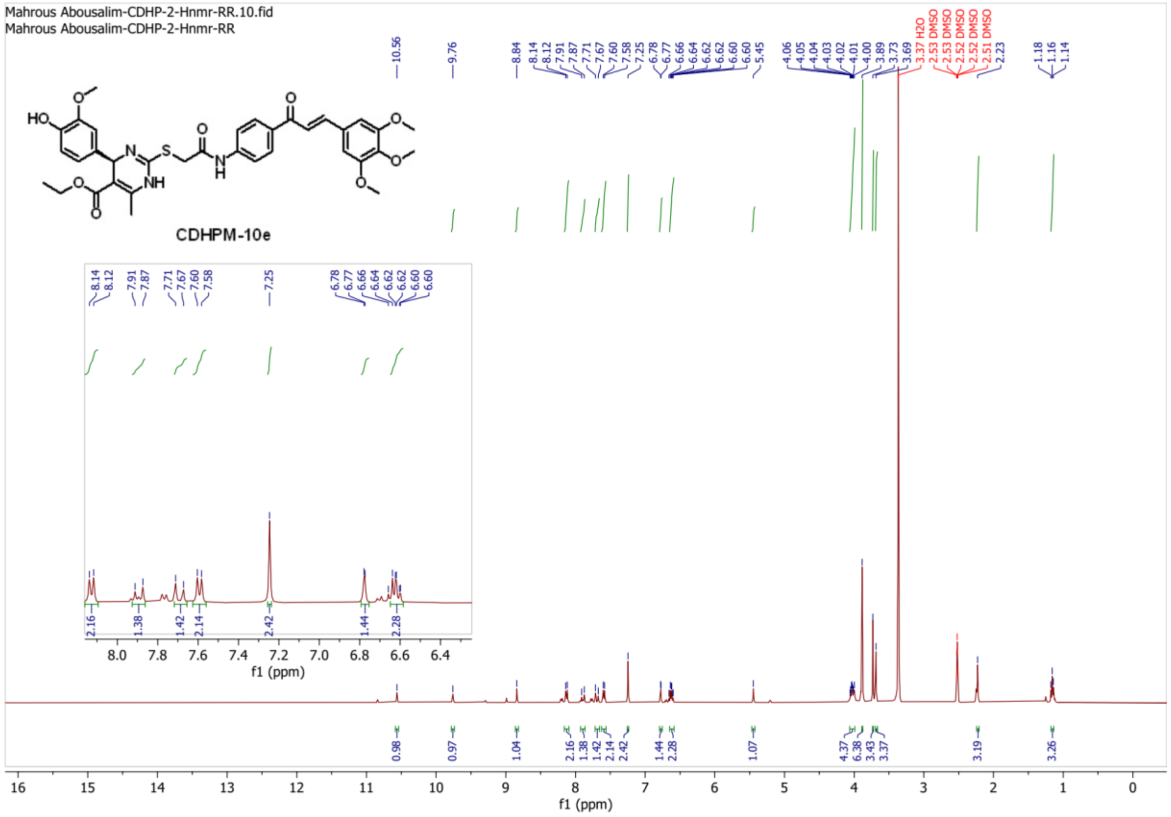

Figure S 23. ^{1}H NMR (400 MHz, DMSO-d_6) of **CDHPM-10e.**

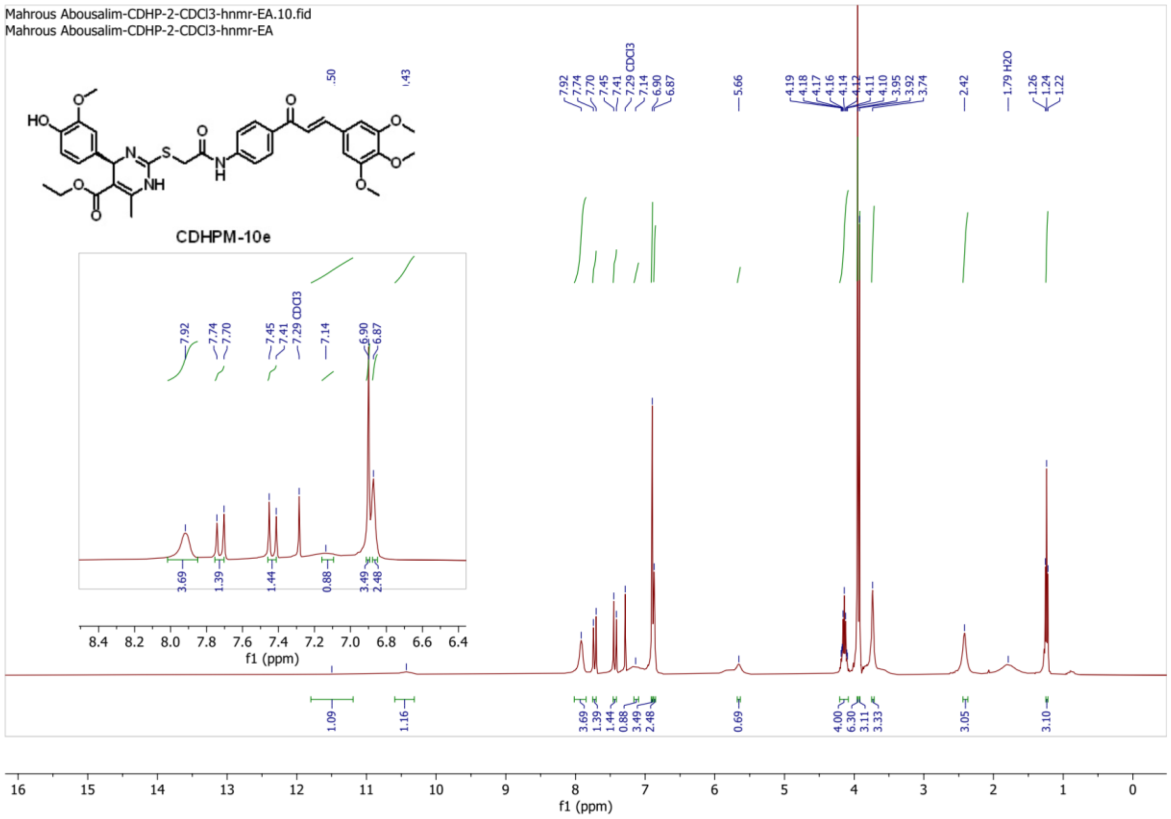

Figure S 24. ^{1}H NMR (400 MHz, $CDCl_3$) of **CDHPM-10e.**

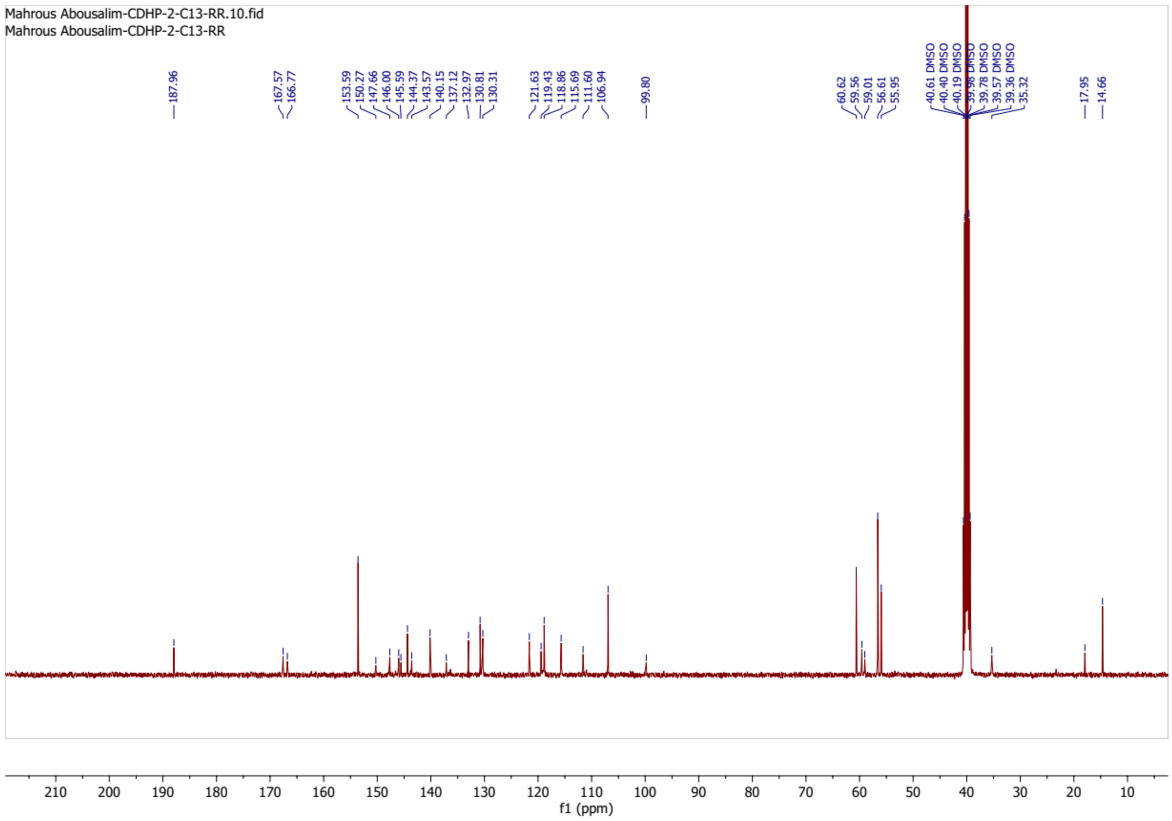

Figure S 25. ^{13}C NMR (101 MHz, DMSO-d_6) of **CDHPM-10e.**

1.14 *CDHPM-10f*

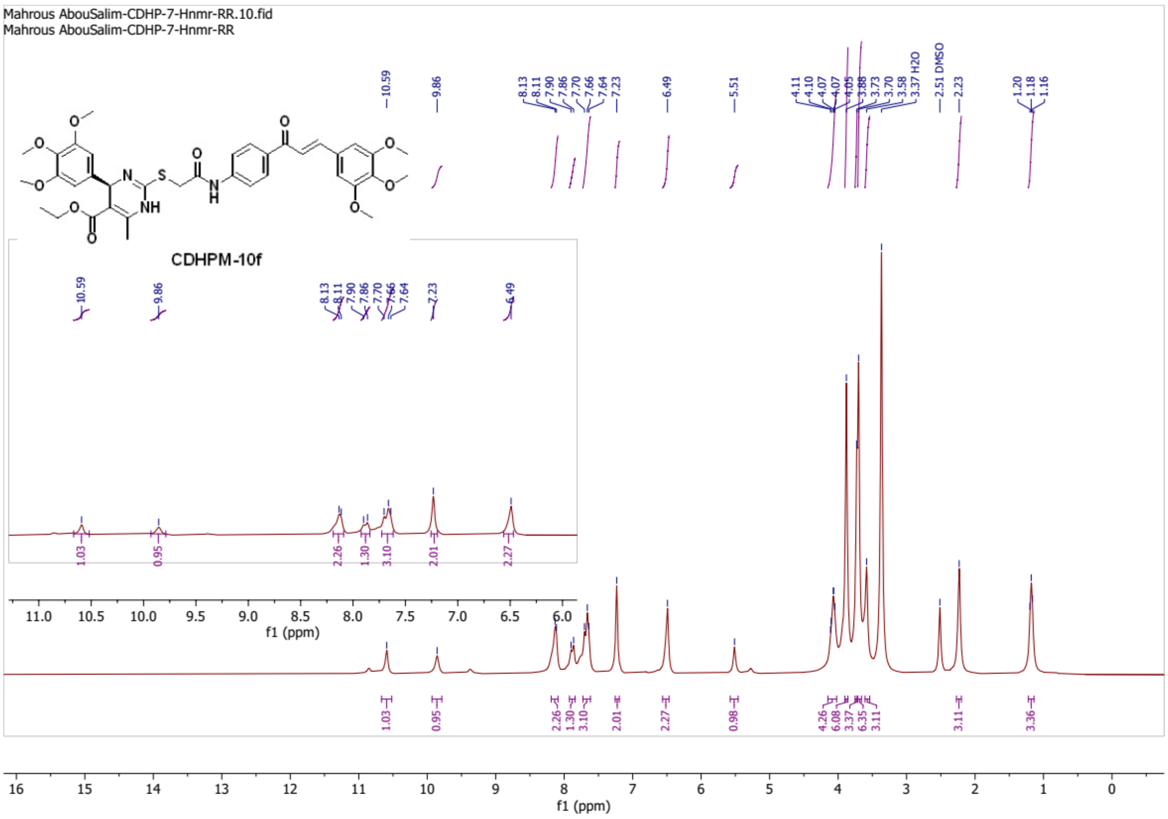

Figure S 26. ^{1}H NMR (400 MHz, DMSO-d_6) of **CDHPM-10f.**

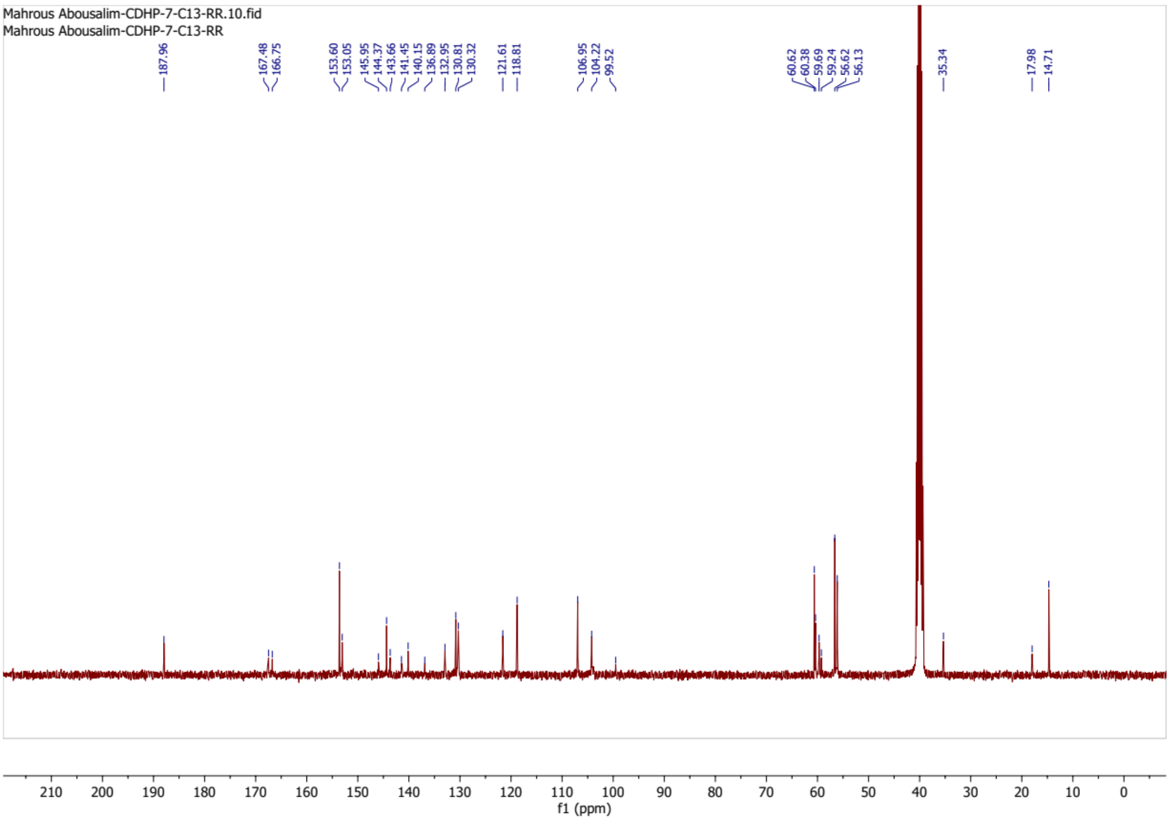

Figure S 27. ^{13}C NMR (101 MHz, DMSO-d_6) of **CDHPM-10f.**

1.15 CDHPM-10g

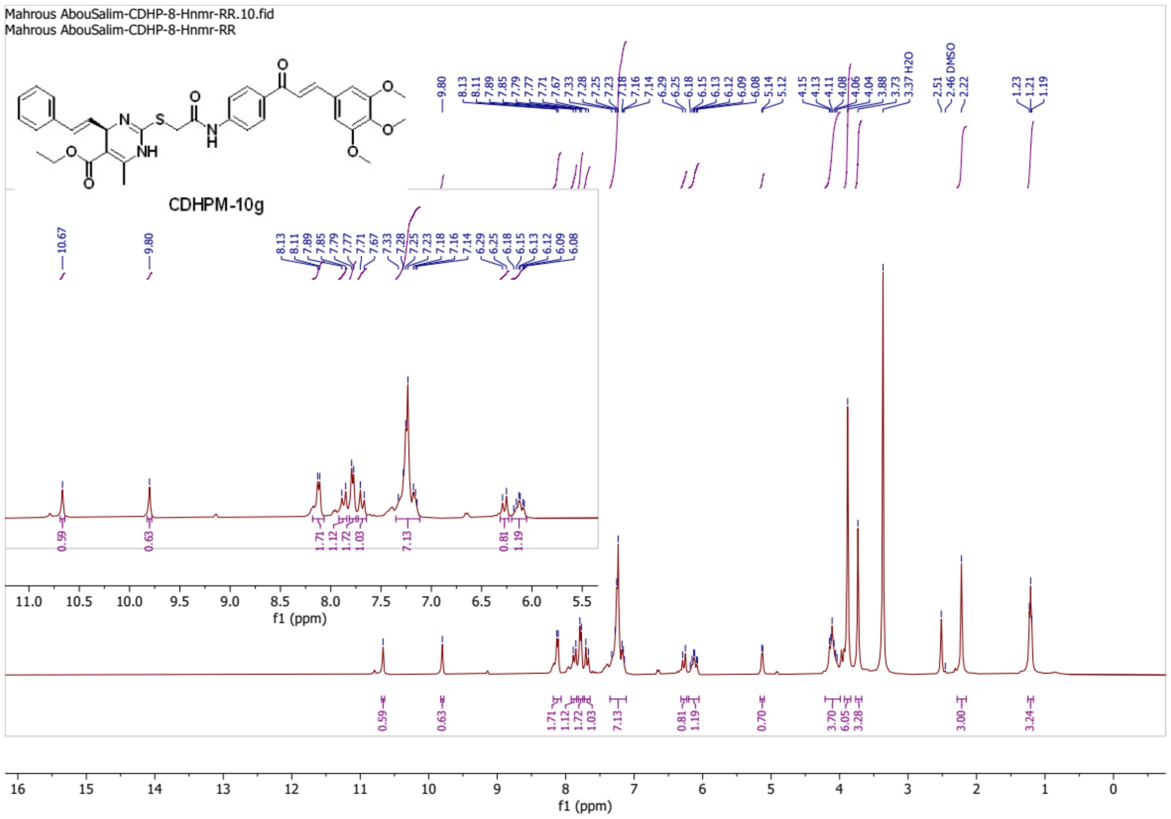

Figure S 28. ^{1}H NMR (400 MHz, DMSO-d_6) of **CDHPM-10g.**

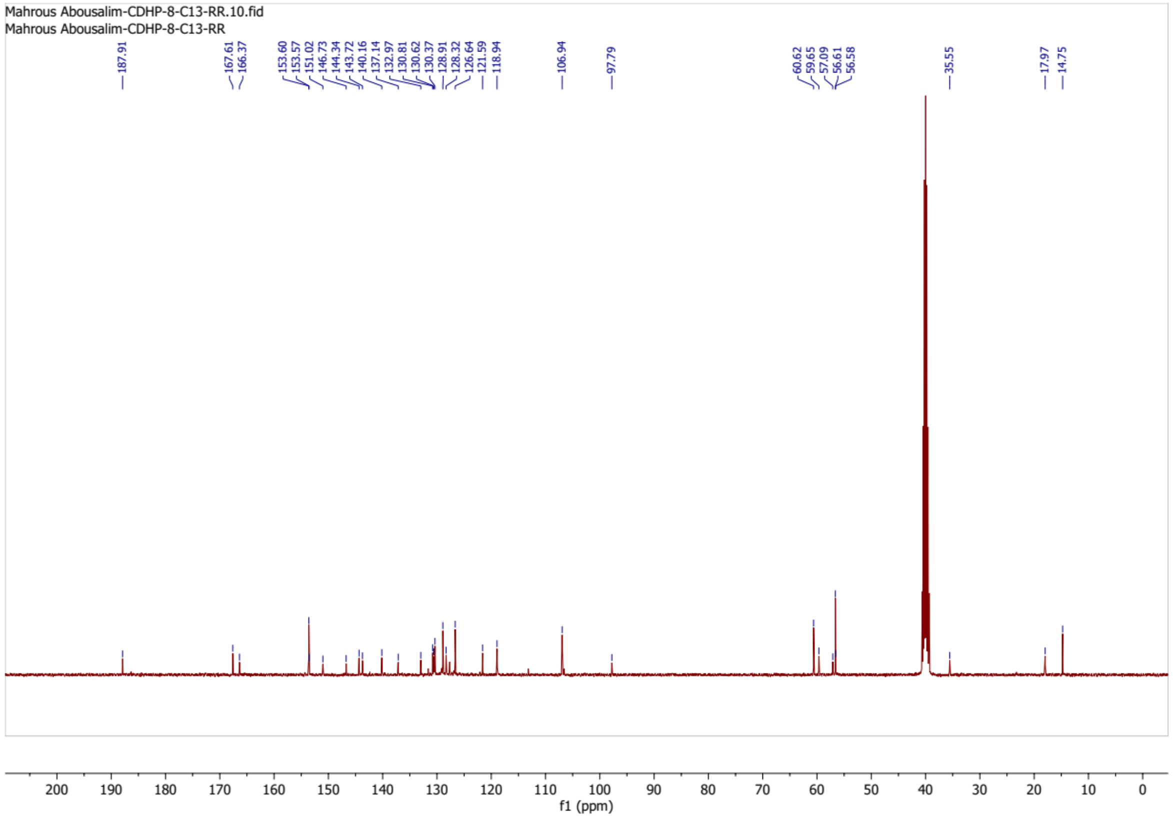

Figure S 29. ^{13}C NMR (101 MHz, DMSO-d_6) of **CDHPM-10g.**

2 NCI-60 cell assay results

2.1 One-dose mean graphs

2.1.1 NO-TZD-3a

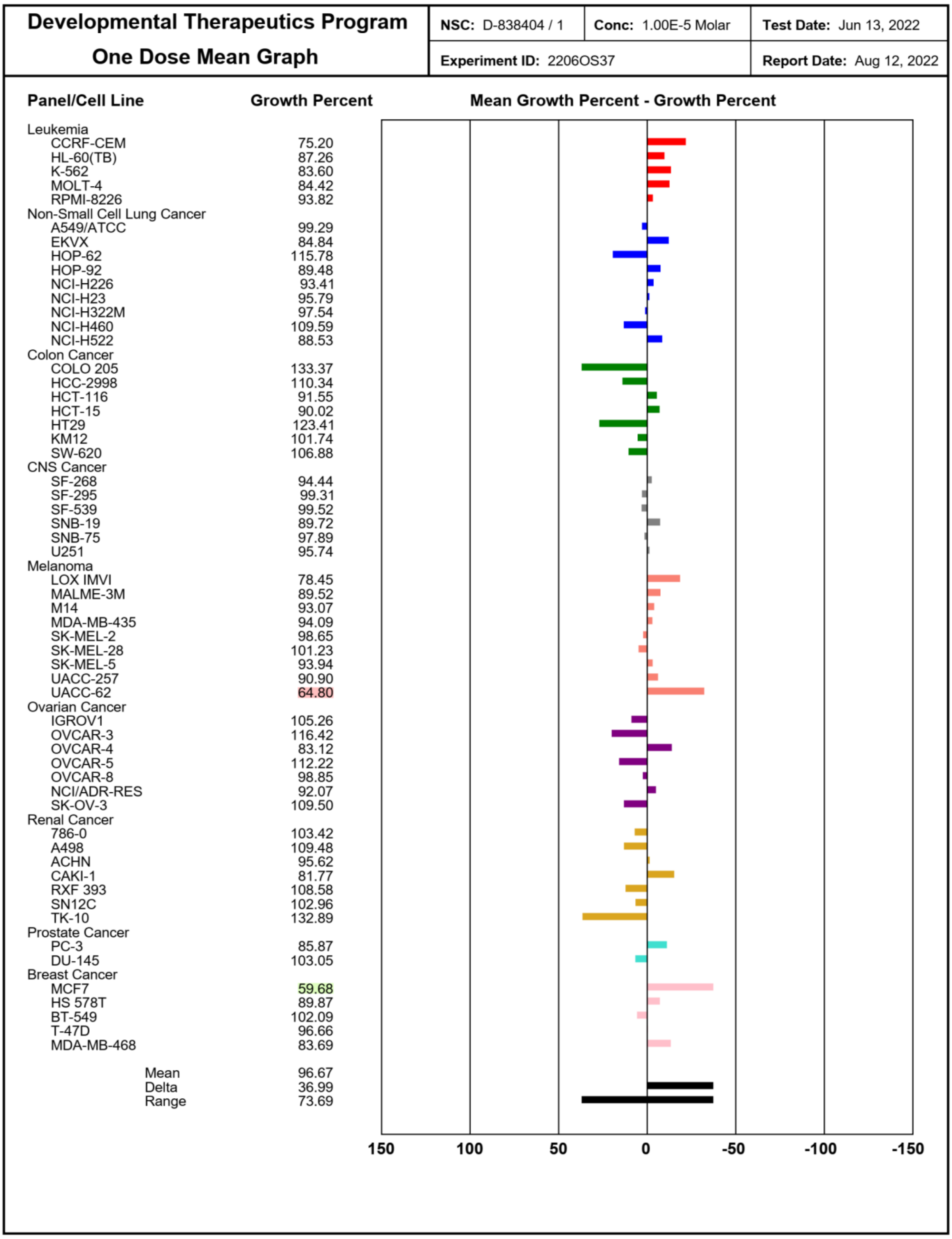

Figure S 30. One-dose mean graph of **NO-TZD-3a**.

2.1.2 NO-TZD-3b

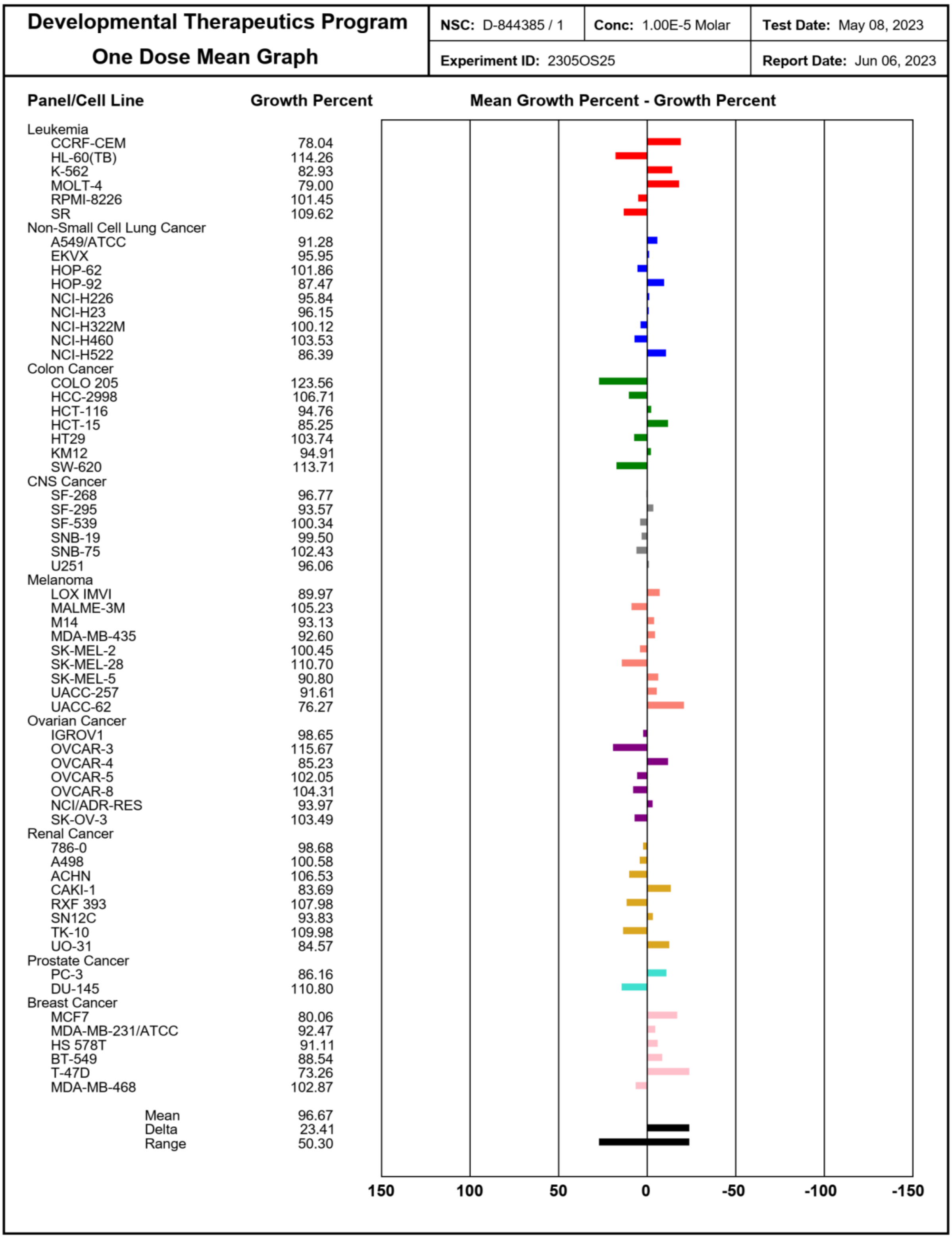

Figure S 31. One-dose mean graph of **NO-TZD-3b**.

2.1.3 NO-TZD-3c

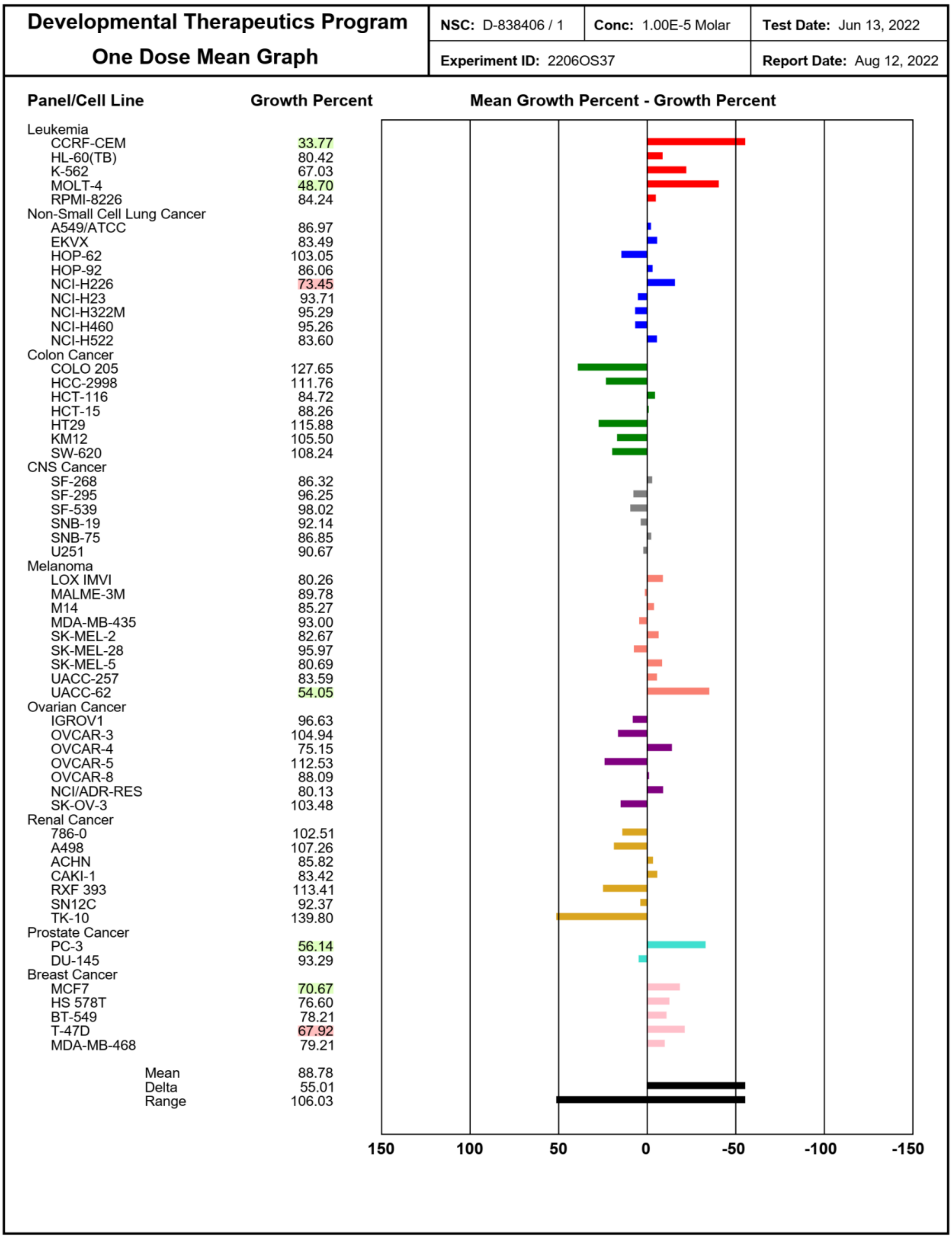

Figure S 32. One-dose mean graph of **NO-TZD-3c**.

2.1.4 NO-TZD-3d

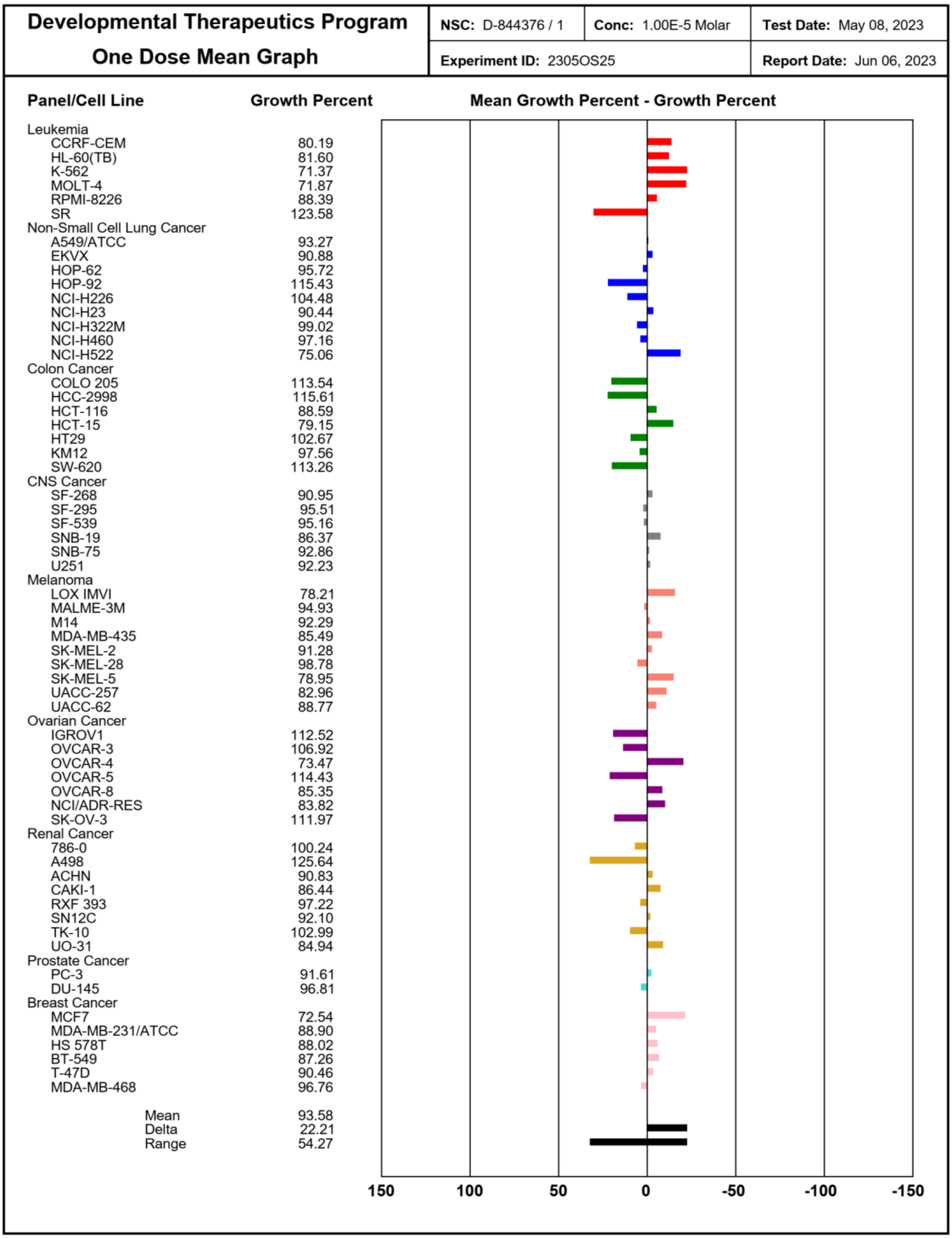

Figure S 33. One-dose mean graph of **NO-TZD-3d**.

2.1.5 NO-TZD-5

Developmental Therapeutics Program One Dose Mean Graph	NSC: D-838405 / 1	Conc: 1.00E-5 Molar	Test Date: Jun 13, 2022
	Experiment ID: 2206OS37		Report Date: Aug 12, 2022

Panel/Cell Line	Growth Percent
Leukemia	
CCRF-CEM	97.63
HL-60(TB)	89.55
K-562	99.85
MOLT-4	100.58
RPMI-8226	104.51
Non-Small Cell Lung Cancer	
A549/ATCC	94.80
EKVX	94.56
HOP-62	109.66
HOP-92	96.56
NCI-H226	97.45
NCI-H23	100.61
NCI-H322M	104.53
NCI-H460	108.91
NCI-H522	84.60
Colon Cancer	
COLO 205	122.25
HCC-2998	109.87
HCT-116	99.77
HCT-15	101.31
HT29	125.55
KM12	106.11
SW-620	107.08
CNS Cancer	
SF-268	96.30
SF-295	106.24
SF-539	97.81
SNB-19	100.20
SNB-75	82.29
U251	96.49
Melanoma	
LOX IMVI	90.39
MALME-3M	88.52
M14	100.59
MDA-MB-435	98.63
SK-MEL-2	104.52
SK-MEL-28	97.74
SK-MEL-5	101.94
UACC-257	94.93
UACC-62	84.93
Ovarian Cancer	
IGROV1	110.06
OVCAR-3	111.41
OVCAR-4	96.90
OVCAR-5	112.27
OVCAR-8	101.50
NCI/ADR-RES	98.61
SK-OV-3	101.31
Renal Cancer	
786-0	99.61
A498	97.42
ACHN	99.95
CAKI-1	89.90
RXF 393	115.20
SN12C	111.15
TK-10	136.25
Prostate Cancer	
PC-3	93.36
DU-145	100.48
Breast Cancer	
MCF7	66.43
HS 578T	96.41
BT-549	97.84
T-47D	95.01
MDA-MB-468	88.85
Mean	100.30
Delta	33.87
Range	69.82

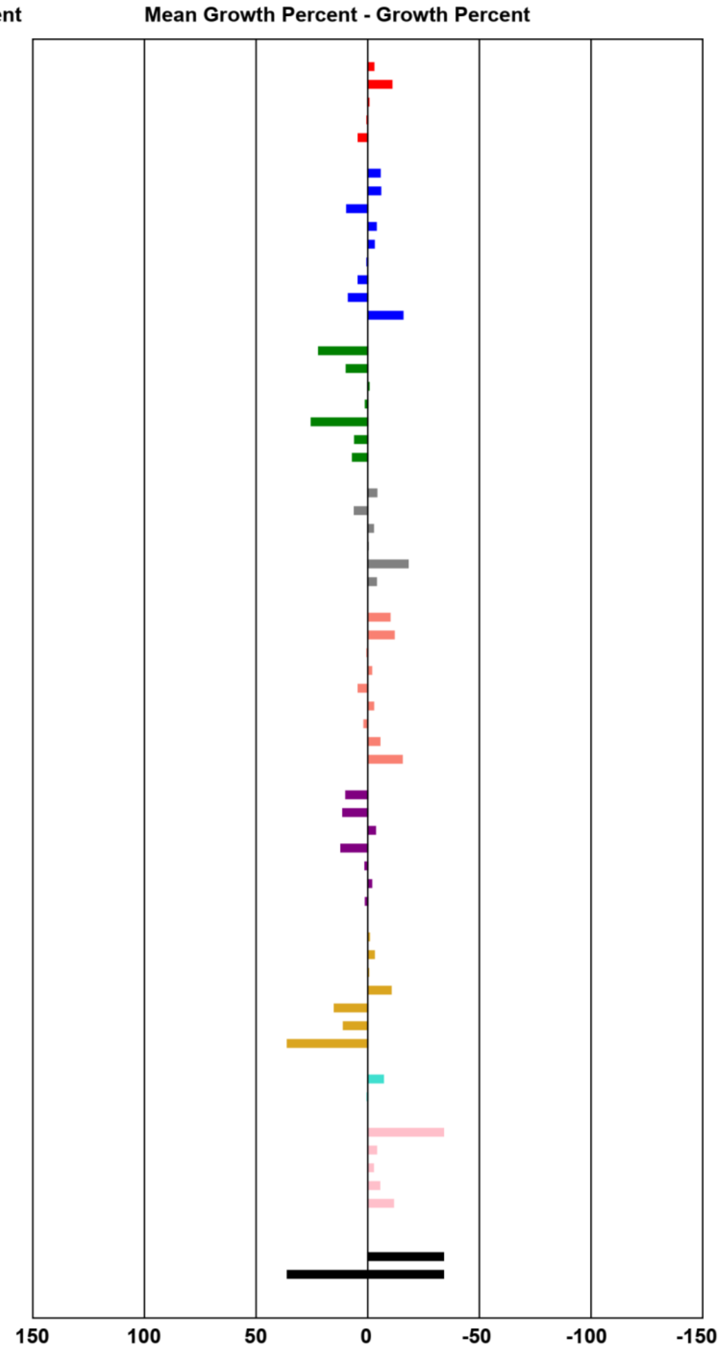

Figure S 34. One-dose mean graph of **NO-TZD-5**.

2.1.6 NO-TZD-6

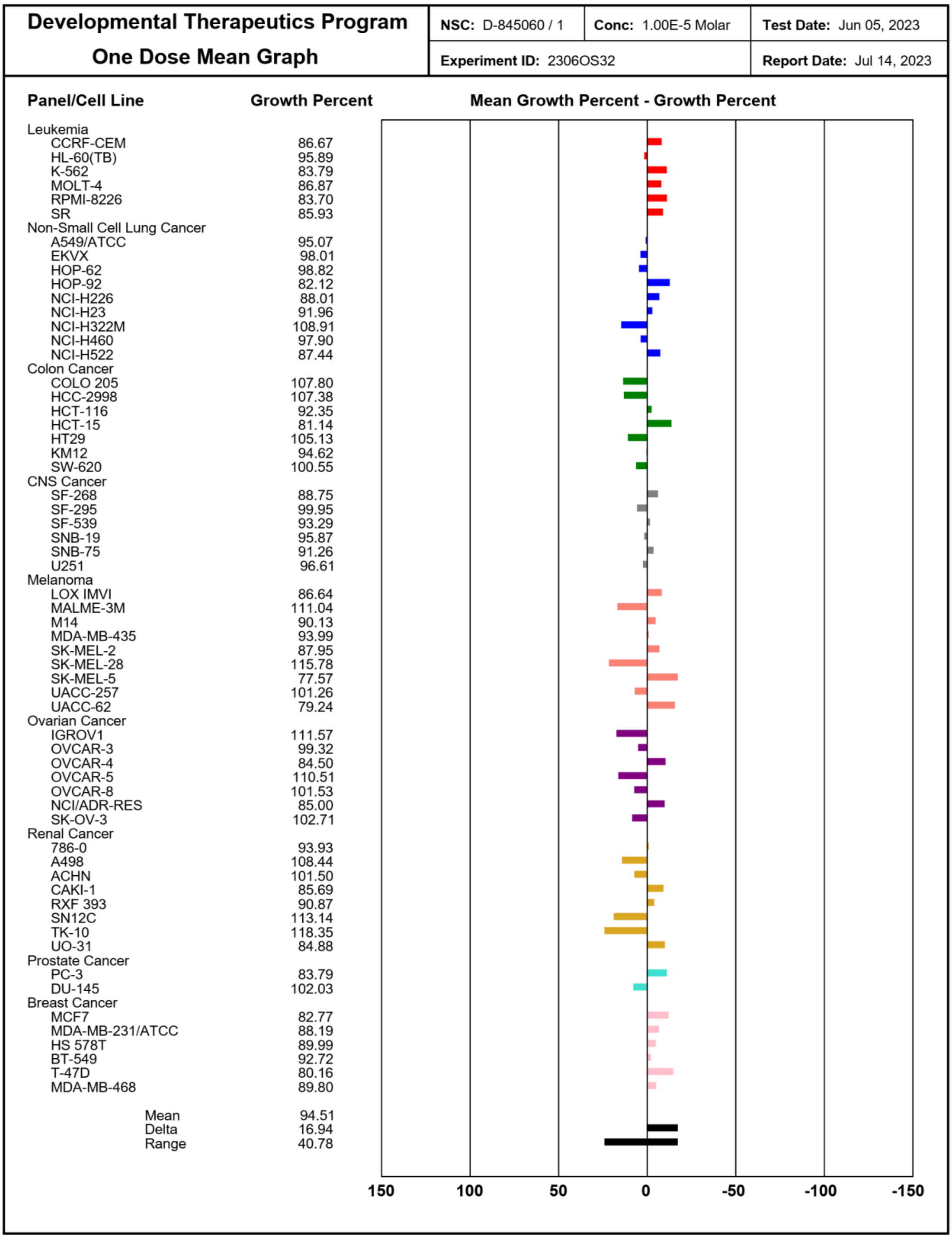

Figure S 35. One-dose mean graph of **NO-TZD-6**.

2.1.7 CDHPM-10a

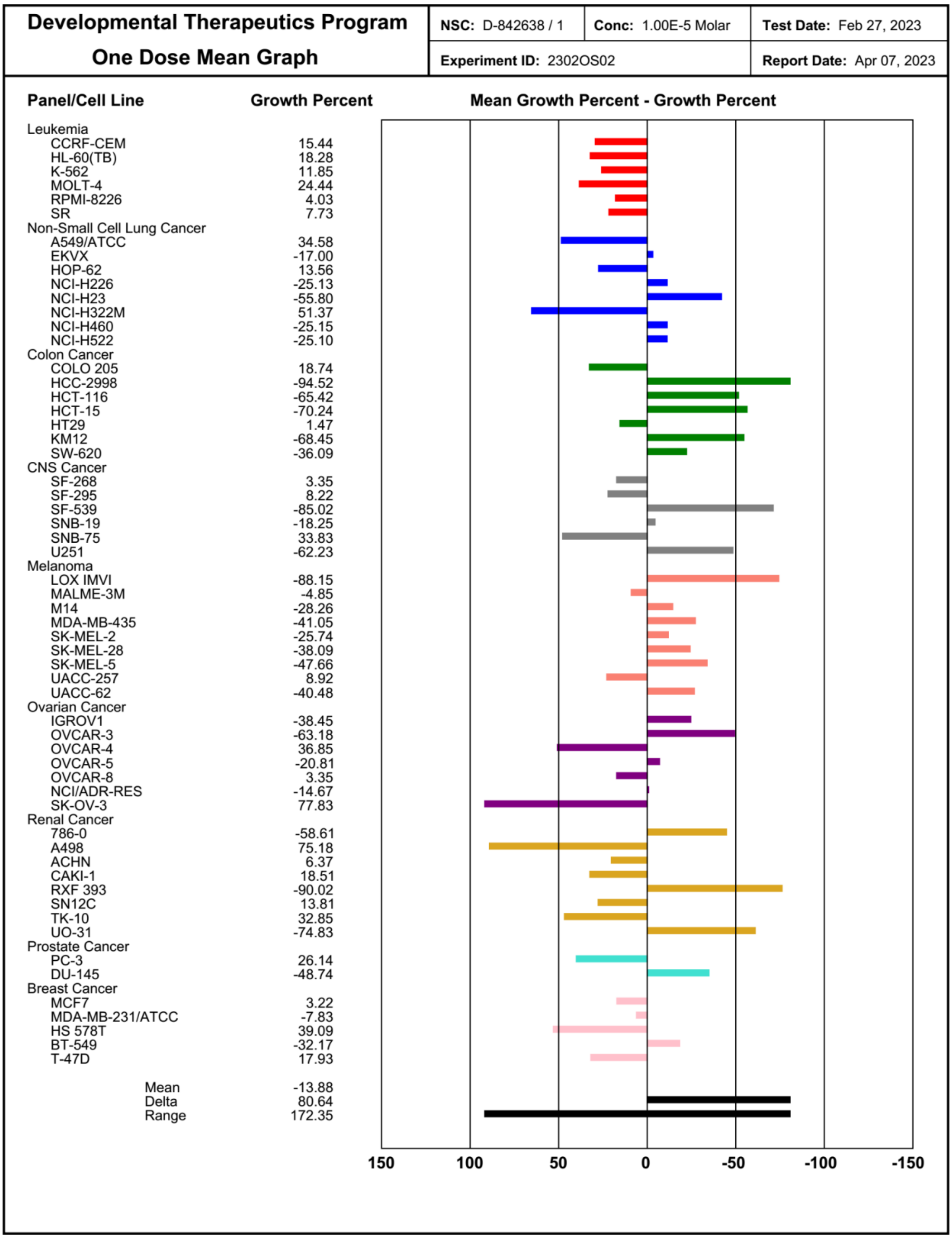

Figure S 36. One-dose mean graph of compound **CDHPM-10a.**

2.1.8 CDHPM-10b

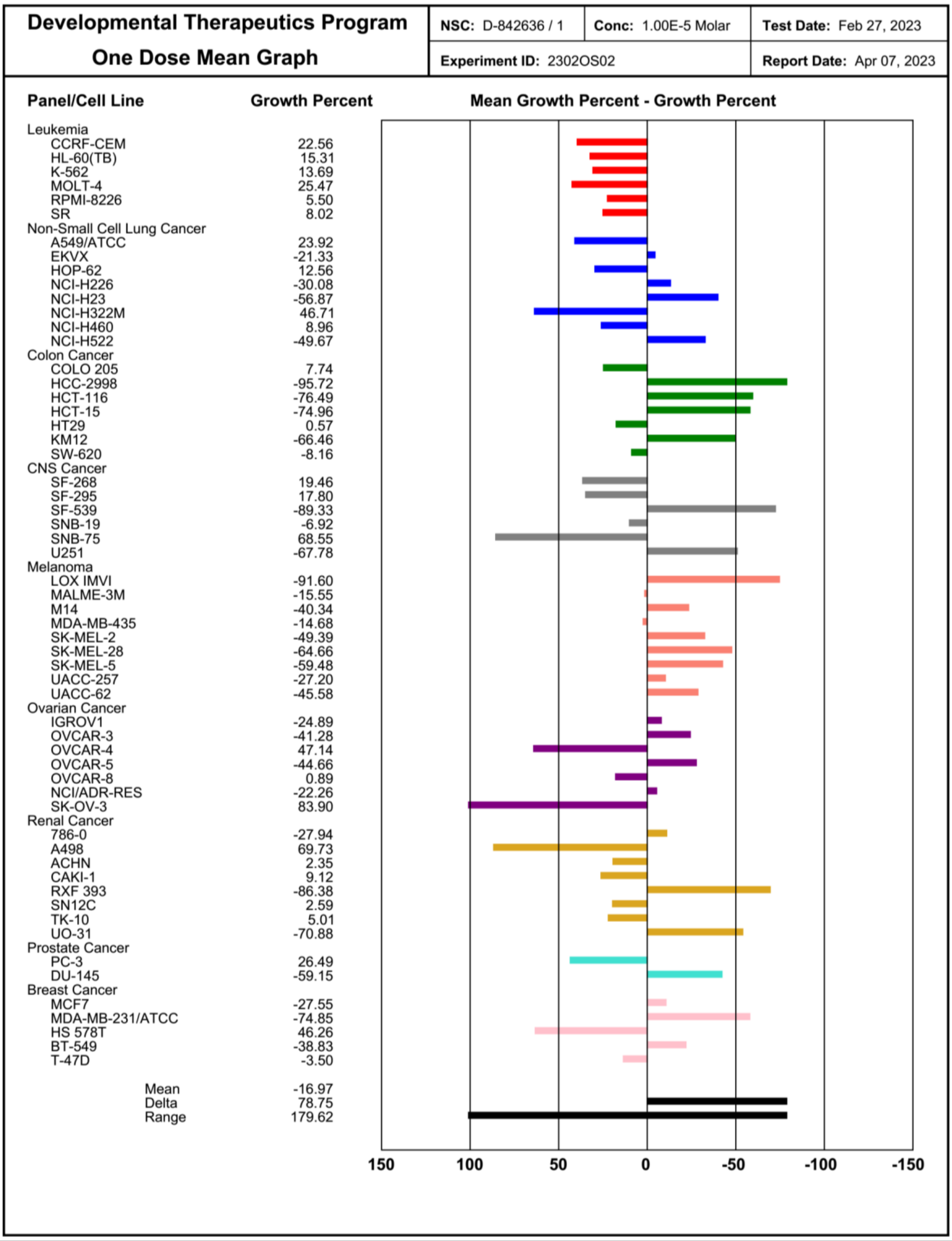

Figure S 37. One-dose mean graph of compound **CDHPM-10b**.

2.1.9 *CDHPM-10c*

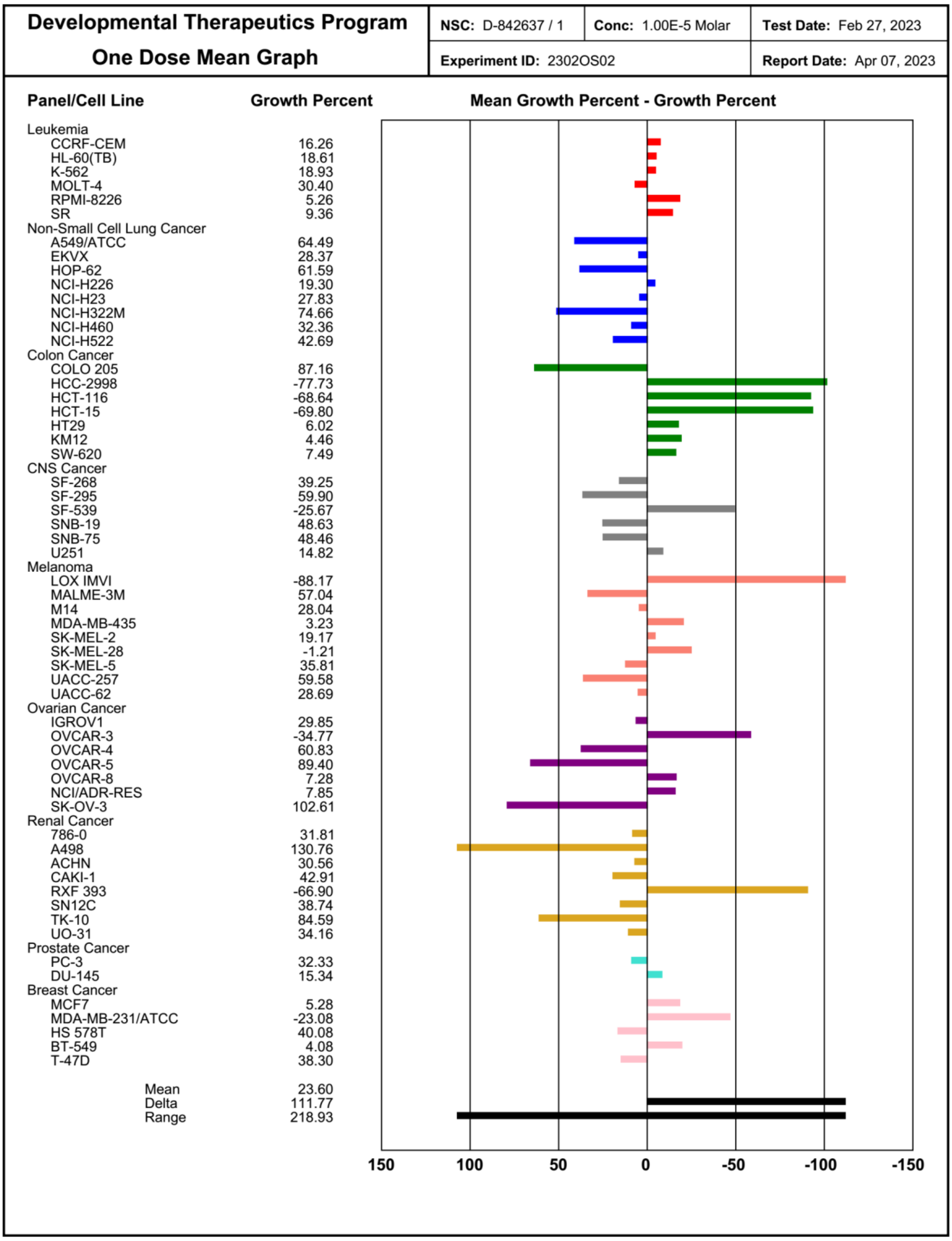

Figure S 38. One-dose mean graph of compound **CDHPM-10c.**

2.1.10 CDHPM-10d

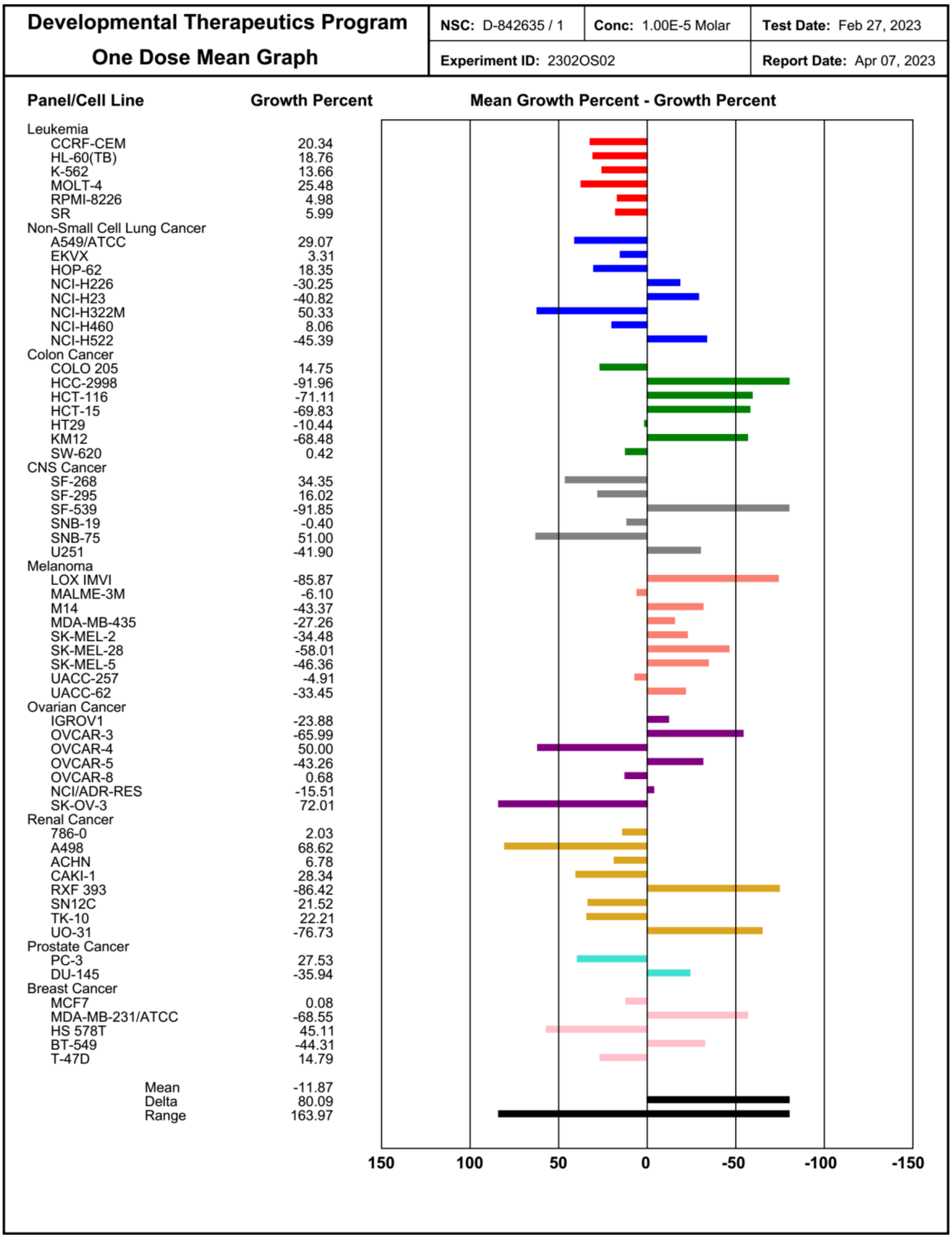

Figure S 39. One-dose mean graph of compound **CDHPM-10d**.

2.1.11 CDHPM-10e

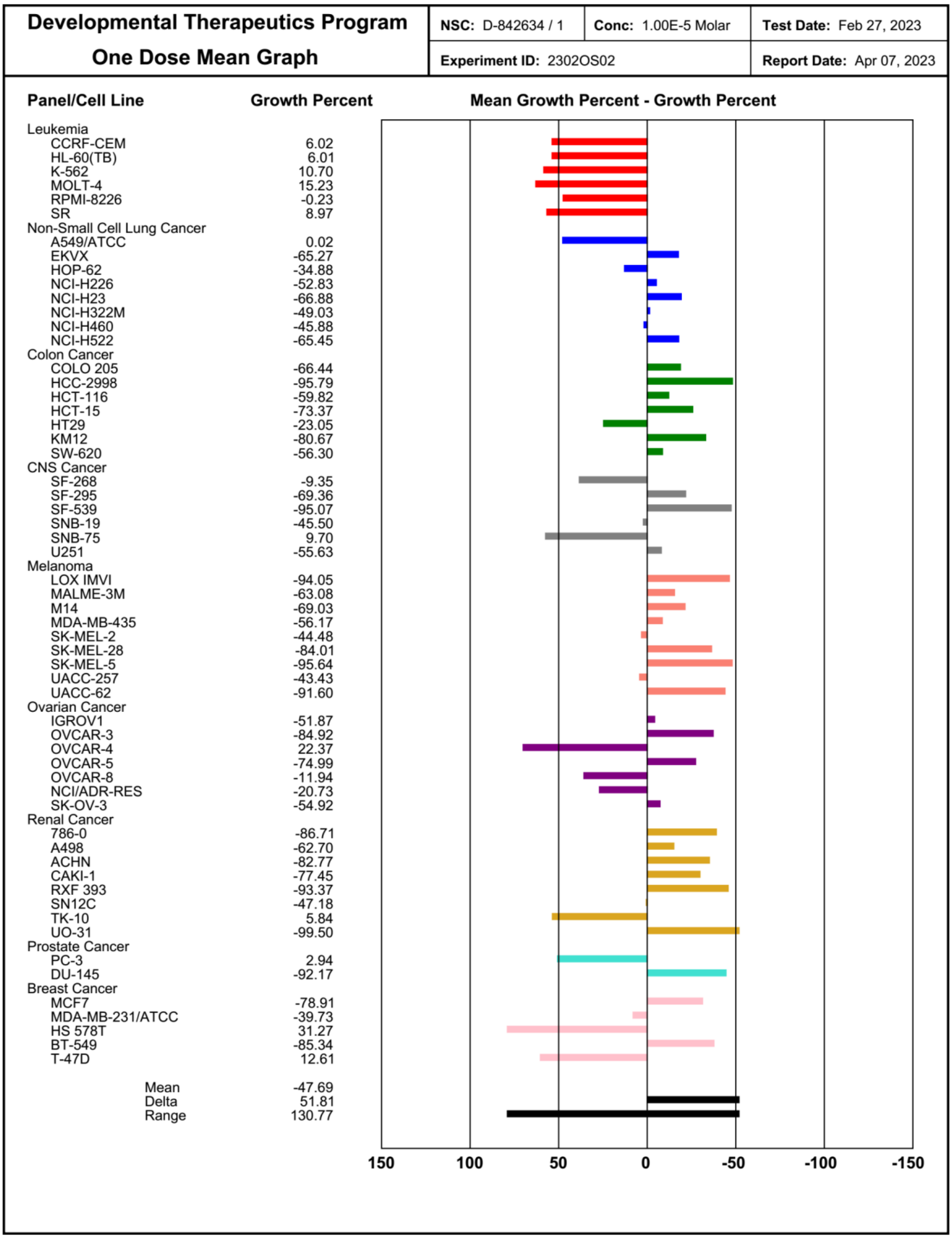

Figure S 40. One-dose mean graph of compound **CDHPM-10e.**

2.1.12 CDHPM-10f

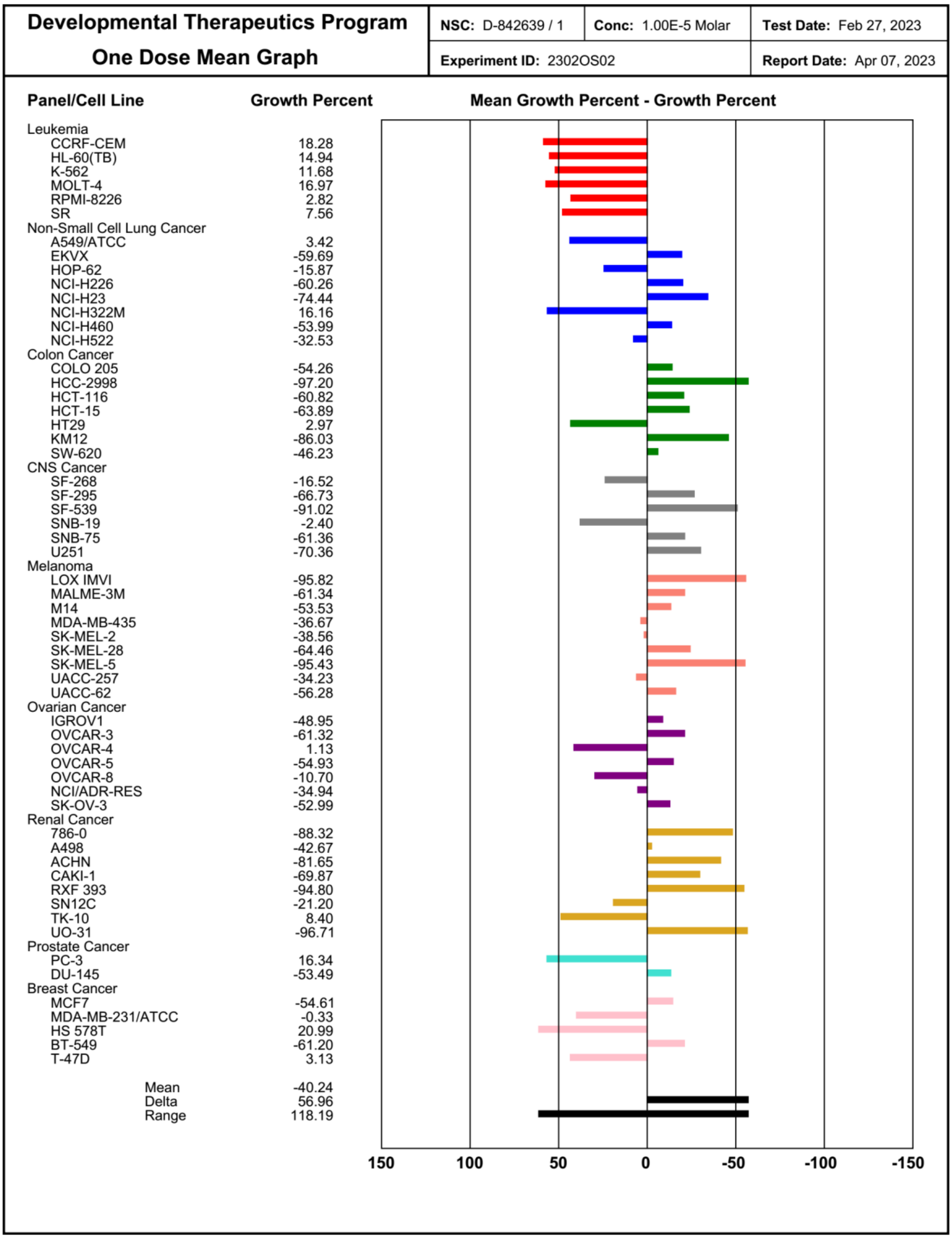

Figure S 41. One-dose mean graph of compound **CDHPM-10f.**

2.1.13 CDHPM-10g

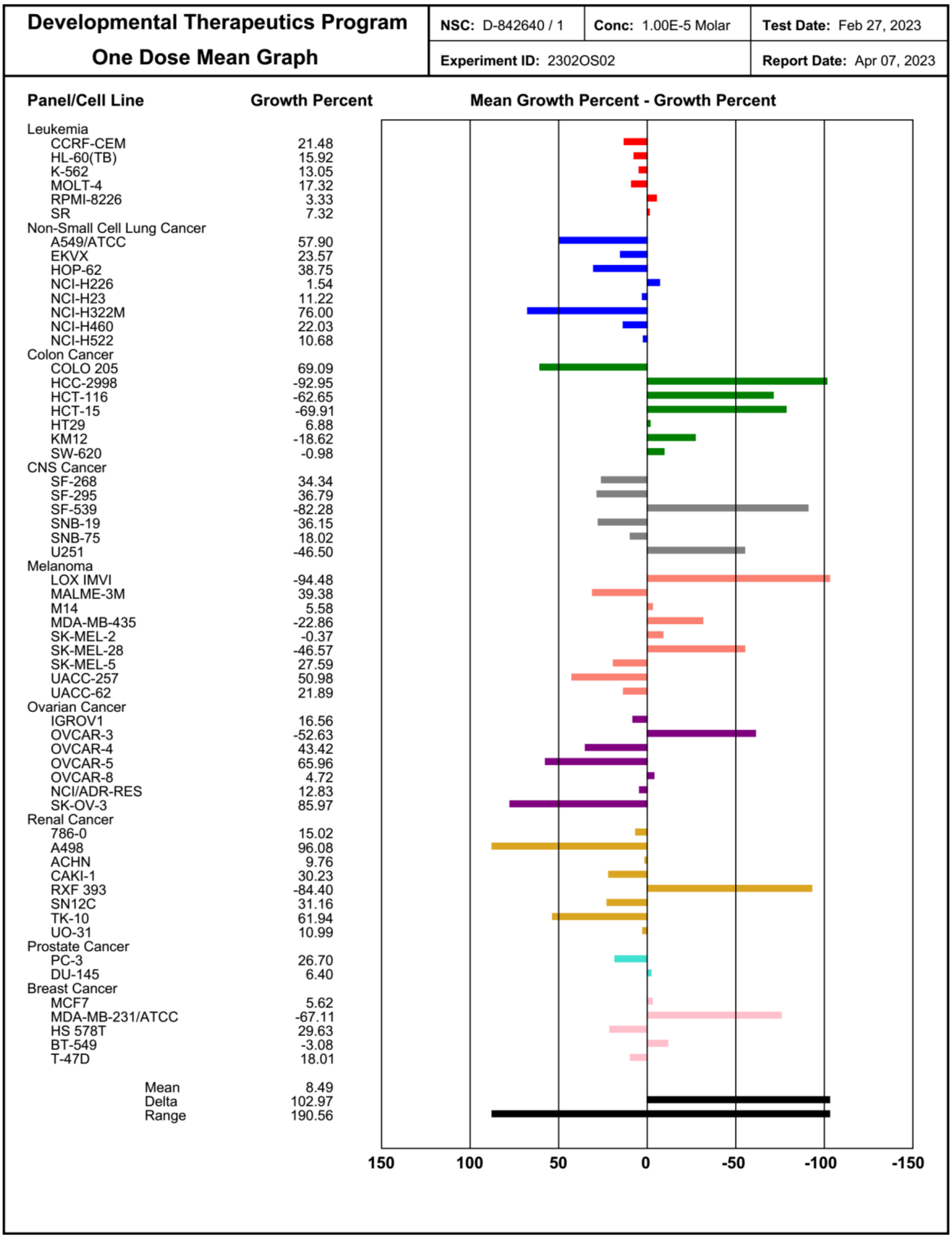

Figure S 42. One-dose mean graph of compound **CDHPM-10g.**

2.2 *Five-dose screen (Dose response curves)*

2.2.1 *CDHPM-10a*

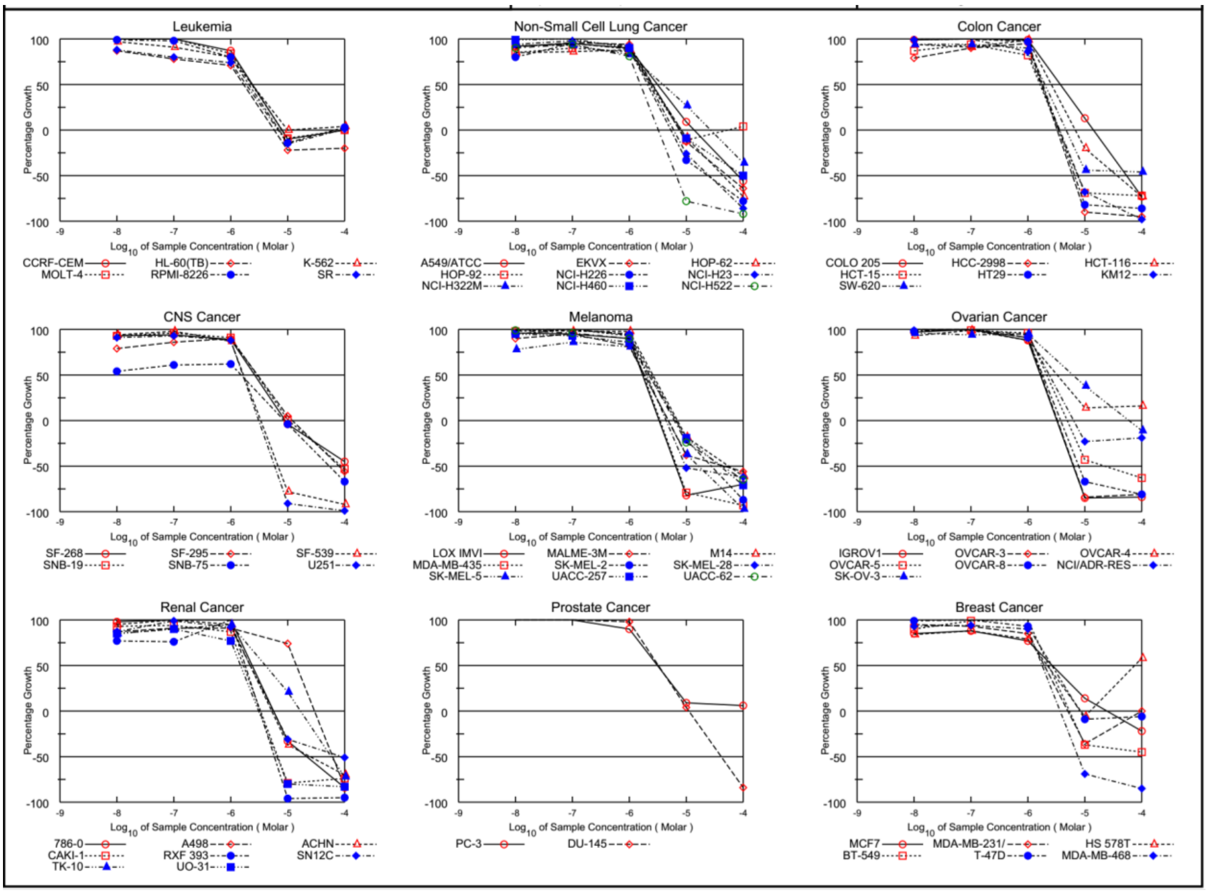

Figure S 43. Dose response curves of compound **CDHPM-10a.**

2.2.2 CDHPM-10b

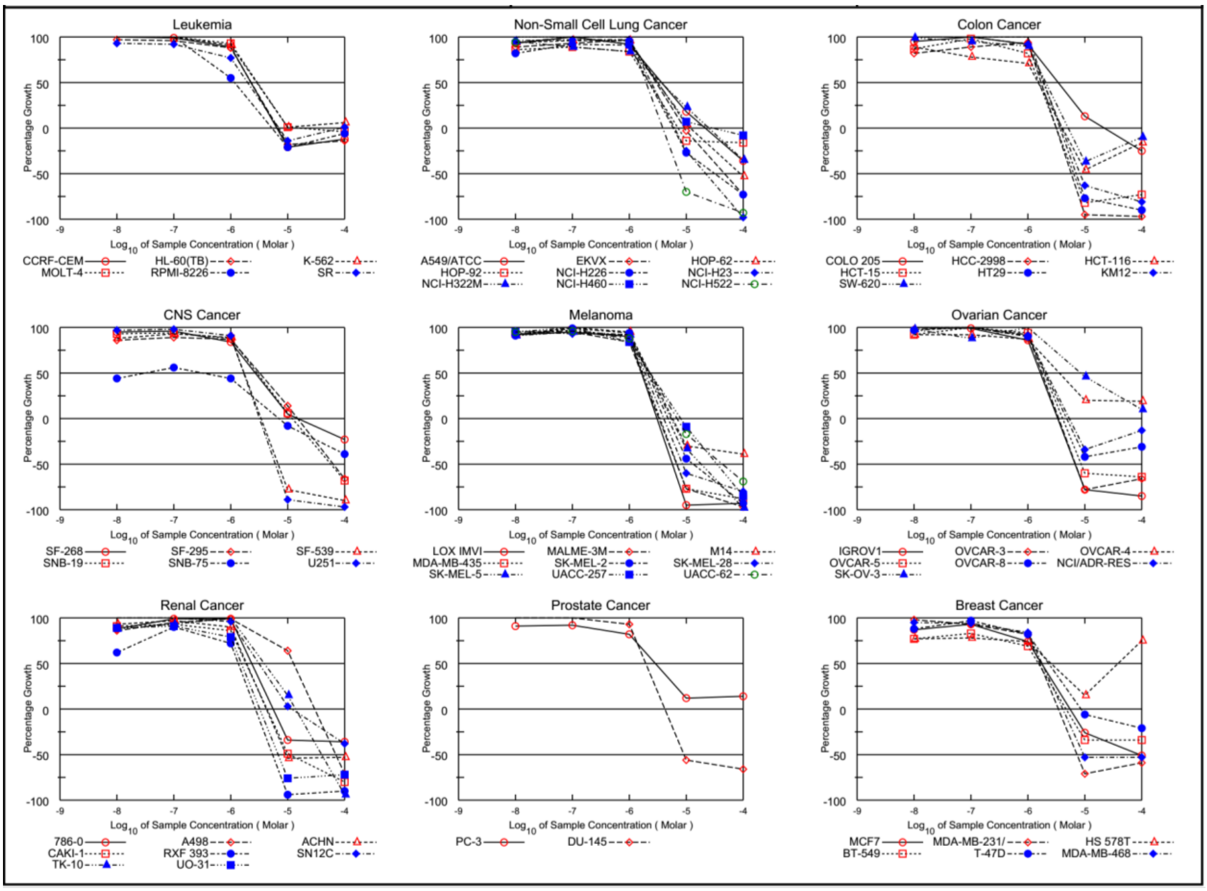

Figure S 44. Dose response curves of compound **CDHPM-10b.**

2.2.3 CDHPM-10c

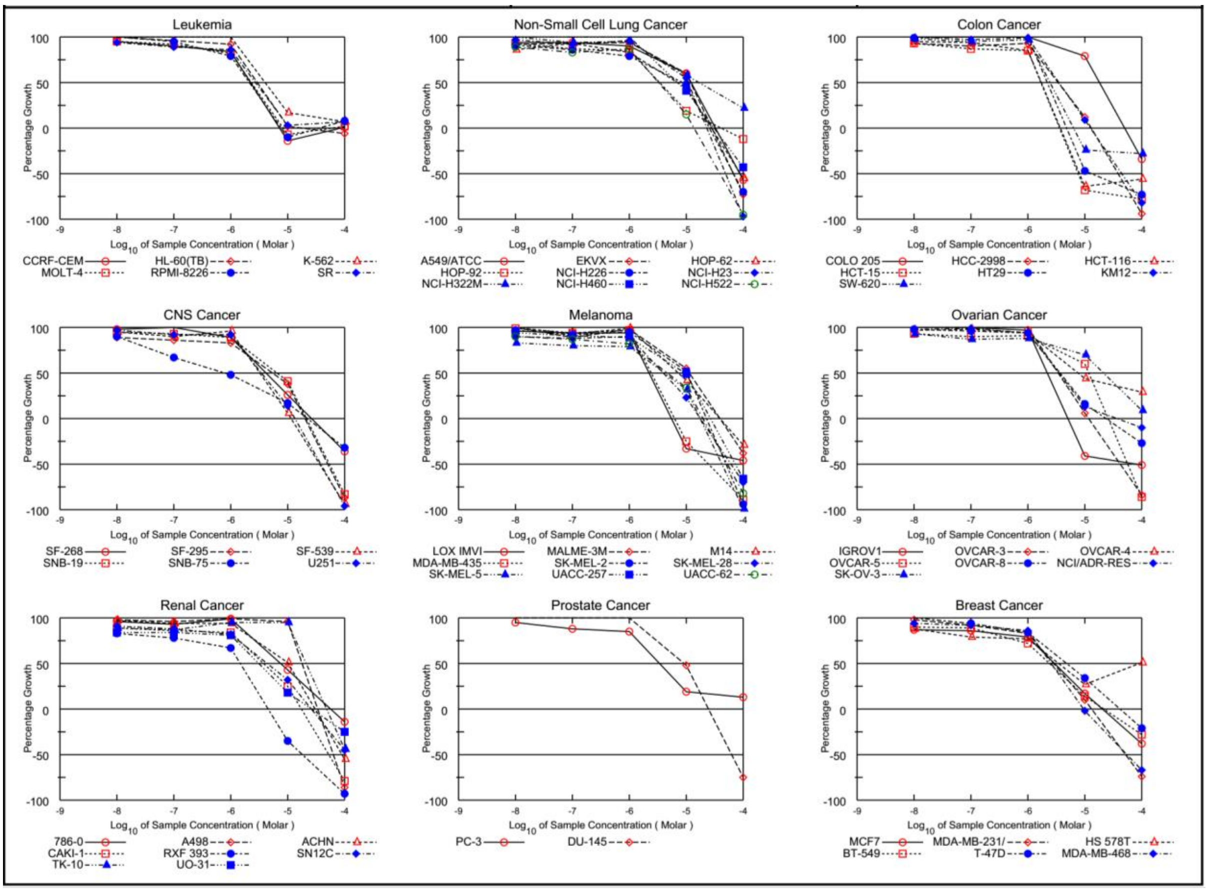

Figure S 45. Dose response curves of compound **CDHPM-10c.**

2.2.4 CDHPM-10d

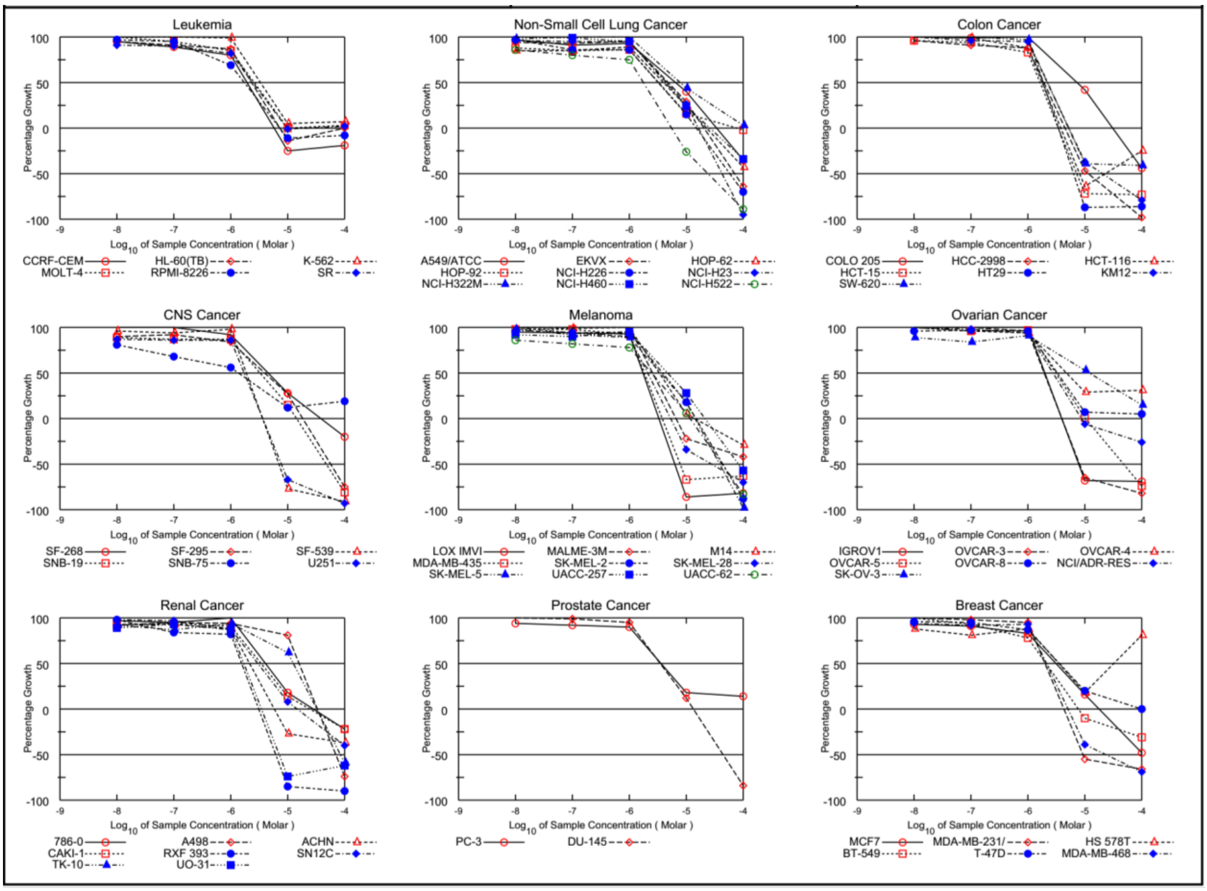

Figure S 46. Dose response curves of compound **CDHPM-10d.**

2.2.5 CDHPM-10f

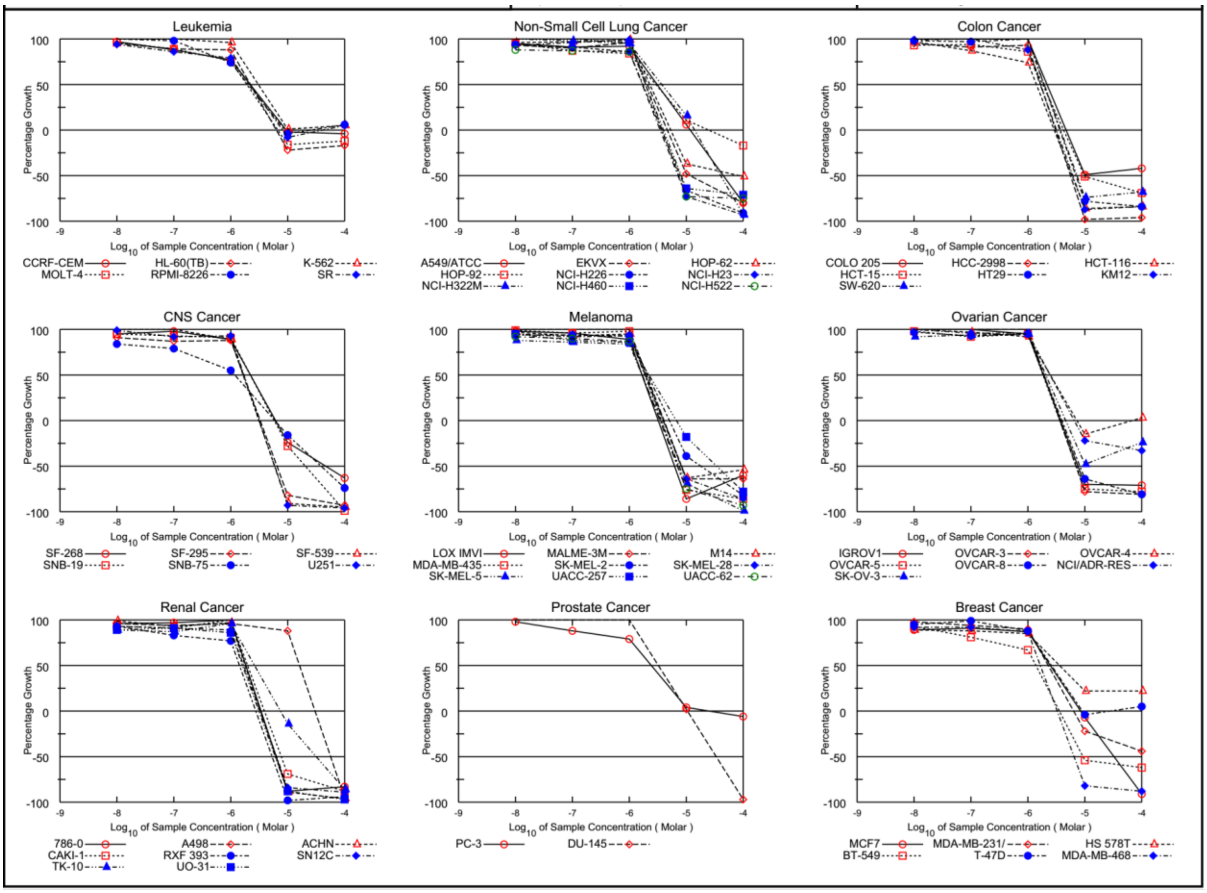

Figure S 47. Dose response curves of compound **CDHPM-10f.**

2.2.6 *CDHPM-10g*

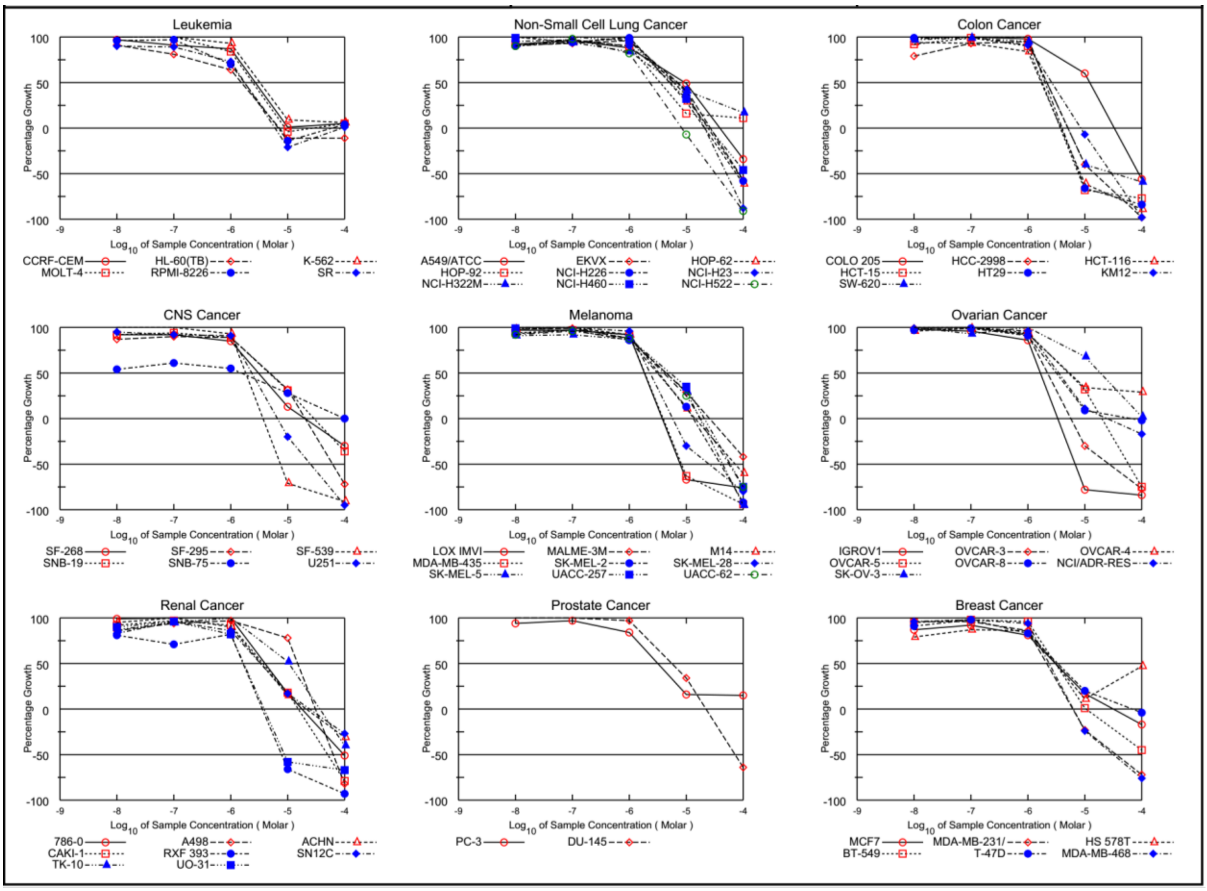

Figure S 48. Dose response curves of compound **CDHPM-10g.**

2.3 TGIs and LC50s

Table S 1. TGI of target compounds **CDHPM-10a-g**.

		**CDHPM-**						
**Panel**	**Cell line**	**10a**	**10b**	**10c**	**10d**	**10e**	**10f**	**10g**
Leukemia	CCRF-CEM	ND	6.40	ND	5.75	5.90	9.36	> 100
	HL-60(TB)	5.76	6.77	10.96	7.30	5.48	6.22	7.08
	K-562	> 100	> 100	> 100	> 100	9.68	> 100	> 100
	MOLT-4	ND	10.50	ND	> 100	7.03	6.69	ND
	RPMI-8226	ND	5.27	ND	7.37	5.76	ND	ND
	SR	ND	ND	> 100	ND	ND	ND	ND
Non-Small Cell Lung Cancer	A549/ATCC	10.39	20.19	13.27	30.44	7.33	10.18	30.88
	EKVX	7.60	9.42	20.82	20.04	3.99	4.61	20.65
	HOP-62	8.28	10.19	20.78	20.28	3.06	5.33	20.09
	HOP-92	ND	7.16	40.04	80.21	6.80	20.42	> 100
	NCI-H226	5.36	5.87	20.56	10.50	3.50	3.70	20.41
	NCI-H23	6.00	6.20	20.31	10.63	3.31	3.78	20.12
	NCI-H322M	20.70	20.48	> 100	> 100	4.70	10.41	> 100
	NCI-H460	8.16	20.82	30.06	20.62	3.88	3.97	20.55
	NCI-H522	3.22	3.91	10.36	5.50	2.97	3.48	8.37
Colon Cancer	COLO 205	10.42	20.21	40.97	30.08	3.72	4.79	30.28
	HCC-2998	3.25	3.14	10.30	4.50	3.10	3.06	5.09
	HCT-116	6.83	4.04	3.73	3.81	4.48	2.92	3.79
	HCT-15	3.51	3.17	3.62	3.45	3.29	4.23	3.71
	HT29	3.48	3.78	4.88	3.43	3.63	3.64	4.00
	KM12	3.60	3.92	10.26	5.27	2.84	3.18	8.50
	SW-620	4.70	5.08	6.37	5.20	3.55	3.83	5.03
CNS Cancer	SF-268	8.99	10.48	20.65	30.83	4.04	6.12	20.05
	SF-295	10.22	10.49	20.03	10.84	3.36	3.31	20.03
	SF-539	3.37	3.41	10.15	3.63	2.95	3.12	3.68
	SNB-19	10.06	10.19	20.15	10.43	8.83	5.82	20.90
	SNB-75	8.75	7.12	20.22	> 100	3.49	5.95	90.62
	U251	3.11	3.20	10.35	3.65	3.14	3.16	6.55
Melanoma	LOX IMVI	3.33	3.05	5.50	3.30	2.80	3.23	3.79

Panel	Cell line	CDHPM-						
		10a	10b	10c	10d	10e	10f	10g
	MALME-3M	4.84	3.34	30.93	6.63	2.98	4.06	20.57
	M14	6.98	5.72	30.89	10.44	4.32	3.99	10.42
	MDA-MB-435	3.47	3.46	6.23	3.78	4.46	3.66	3.90
	SK-MEL-2	6.59	4.78	20.27	10.47	3.90	5.08	10.33
	SK-MEL-28	4.43	4.03	10.76	5.31	3.98	3.90	5.76
	SK-MEL-5	4.83	5.45	10.76	10.44	3.04	3.53	10.73
	UACC-257	6.56	7.96	20.66	20.11	5.17	6.77	20.09
	UACC-62	6.17	6.85	10.98	10.16	3.04	3.45	10.78
Ovarian Cancer	IGROV1	3.22	3.34	5.08	3.99	3.15	3.76	3.36
	OVCAR-3	3.31	3.49	10.16	3.94	3.43	3.68	5.63
	OVCAR-4	> 100	> 100	> 100	> 100	> 100	ND	> 100
	OVCAR-5	4.93	4.09	20.57	10.04	3.53	3.61	20.01
	OVCAR-8	3.77	4.81	20.33	> 100	5.32	3.96	60.05
	NCI/ADR-RES	6.39	5.32	30.63	8.66	5.08	6.52	20.45
	SK-OV-3	5.93	> 100	> 100	> 100	3.76	4.65	> 100
Renal Cancer	786-0	5.75	5.53	50.68	20.86	3.80	3.42	10.77
	A498	20.95	20.98	30.36	30.34	7.24	30.04	30.07
	ACHN	5.25	4.22	30.03	5.81	3.14	3.32	20.14
	CAKI-1	3.34	4.33	10.74	20.46	3.27	3.96	10.53
	RXF 393	3.26	2.72	4.56	3.09	2.43	2.76	3.56
	SN12C	5.60	10.17	20.59	10.46	3.35	3.26	20.46
	TK-10	10.69	10.36	40.79	30.26	5.71	7.51	30.68
	UO-31	3.10	3.23	20.60	3.48	2.60	3.12	3.83
Prostate Cancer	PC-3	> 100	> 100	> 100	> 100	6.55	20.56	> 100
	DU-145	10.11	4.23	20.46	10.33	3.20	10.04	20.23
Breast Cancer	MCF7	20.40	5.53	20.04	10.78	4.31	8.44	30.16
	MDA-MB-231/ATCC	ND	3.43	10.33	4.32	4.37	6.35	6.11
	HS 578T	ND	> 100	> 100	> 100	30.79	> 100	> 100
	BT-549	5.50	4.76	20.12	7.76	3.69	3.57	10.07

Panel	Cell line	CDHPM-						
		10a	10b	10c	10d	10e	10f	10g
	T-47D	8.24	8.61	40.18	90.84	9.81	ND	60.71
	MDA-MB-468	3.70	4.10	9.52	5.05	3.49	3.26	6.27

ND: Not determined

Table S 2. LC_{50} of target compounds **CDHPM-10a-g**.

Panel	Cell line	CDHPM-						
		**10a**	**10b**	**10c**	**10d**	**10e**	**10f**	**10g**
Leukemia	CCRF-CEM	> 100	> 100	> 100	> 100	> 100	> 100	> 100
	HL-60(TB)	> 100	> 100	> 100	> 100	> 100	> 100	> 100
	K-562	> 100	> 100	> 100	> 100	> 100	> 100	> 100
	MOLT-4	> 100	> 100	> 100	> 100	> 100	> 100	> 100
	RPMI-8226	> 100	> 100	> 100	> 100	> 100	> 100	> 100
	SR	> 100	> 100	> 100	> 100	> 100	> 100	> 100
Non-Small Cell Lung Cancer	A549/ATCC	80.19	> 100	80.77	> 100	30.79	40.46	> 100
	EKVX	50.27	40.67	60.70	17.05	8.10	10.12	80.46
	HOP-62	40.45	80.87	80.96	> 100	6.45	80.34	70.59
	HOP-92	> 100	> 100	> 100	> 100	70.60	> 100	> 100
	NCI-H226	20.38	30.12	60.75	50.80	6.74	7.89	80.26
	NCI-H23	20.49	20.18	40.93	40.24	6.43	7.39	50.14
	NCI-H322M	> 100	> 100	> 100	> 100	10.51	14.04	> 100
	NCI-H460	> 100	> 100	> 100	> 100	8.29	8.16	> 100
	NCI-H522	6.62	7.65	30.91	20.39	6.22	7.19	30.26
Colon Cancer	COLO 205	50.38	> 100	> 100	> 100	7.29	> 100	80.88
	HCC-2998	6.06	5.76	30.87	10.15	5.65	5.60	10.51
	HCT-116	30.72	> 100	8.05	ND	5.31	5.98	8.38
	HCT-15	7.53	6.38	7.68	7.25	6.99	9.79	7.70
	HT29	6.63	7.09	10.32	6.34	7.18	6.95	8.02
	KM12	7.60	8.23	40.43	20.09	5.86	6.13	20.99
	SW-620	> 100	> 100	> 100	> 100	7.40	7.30	30.22
CNS Cancer	SF-268	> 100	> 100	> 100	> 100	9.26	40.66	> 100
	SF-295	80.06	60.29	50.08	50.72	6.31	6.50	60.18
	SF-539	6.77	6.83	30.63	7.03	5.69	5.90	7.42
	SNB-19	18.92	50.75	50.44	40.73	3.03	20.04	> 100
	SNB-75	50.35	> 100	> 100	> 100	8.78	30.89	> 100
	U251	5.90	6.09	30.83	7.75	5.99	5.87	20.50
Melanoma	LOX IMVI	6.51	5.68	> 100	6.29	5.39	6.21	7.79

Panel	Cell line	CDHPM-						
		10a	10b	10c	10d	10e	10f	10g
	MALME-3M	40.51	6.82	> 100	> 100	6.30	8.20	> 100
	M14	50.63	> 100	> 100	> 100	9.43	8.24	70.19
	MDA-MB-435	6.80	6.91	20.45	7.81	10.08	7.09	8.19
	SK-MEL-2	20.76	10.30	50.01	40.34	8.45	10.81	30.98
	SK-MEL-28	9.70	8.60	60.13	20.76	8.23	8.11	20.51
	SK-MEL-5	10.63	10.83	40.22	30.87	5.65	7.52	40.36
	UACC-257	30.92	30.42	70.28	80.19	2.04	30.41	50.95
	UACC-62	40.30	40.25	50.33	40.25	5.93	7.02	50.62
Ovarian Cancer	IGROV1	6.26	6.74	70.91	7.85	6.39	7.57	6.78
	OVCAR-3	6.38	6.86	40.16	8.04	7.14	6.99	20.62
	OVCAR-4	> 100	> 100	> 100	> 100	> 100	> 100	> 100
	OVCAR-5	20.30	8.62	50.68	40.77	6.98	7.10	50.88
	OVCAR-8	7.82	> 100	> 100	> 100	20.07	8.14	> 100
	NCI/ADR-RES	> 100	> 100	> 100	> 100	> 100	> 100	> 100
	SK-OV-3	> 100	> 100	> 100	> 100	8.90	> 100	> 100
Renal Cancer	786-0	20.14	> 100	> 100	> 100	7.42	6.28	90.70
	A498	60.13	60.98	60.34	60.99	20.98	50.69	60.33
	ACHN	20.47	9.43	80.99	> 100	5.81	6.16	> 100
	CAKI-1	6.70	10.08	50.24	> 100	6.51	7.77	50.04
	RXF 393	5.86	5.42	10.82	6.17	5.05	5.34	7.76
	SN12C	90.11	> 100	> 100	> 100	6.71	6.36	> 100
	TK-10	50.78	30.92	> 100	80.46	10.91	30.19	> 100
	UO-31	6.47	6.77	> 100	7.10	5.10	6.03	8.72
Prostate Cancer	PC-3	> 100	> 100	> 100	> 100	> 100	> 100	> 100
	DU-145	40.13	9.17	60.25	40.42	6.28	30.33	70.21
Breast Cancer	MCF7	> 100	90.11	> 100	> 100	10.71	30.27	> 100
	MDA-MB-231/ATCC	> 100	7.27	50.18	9.32	10.34	> 100	30.57
	HS 578T	> 100	> 100	> 100	> 100	> 100	> 100	> 100
	BT-549	> 100	> 100	> 100	> 100	8.49	9.31	> 100

Panel	Cell line	CDHPM-						
		10a	10b	10c	10d	10e	10f	10g
	T-47D	> 100	> 100	> 100	> 100	> 100	> 100	> 100
	MDA-MB-468	7.65	9.49	50.48	20.33	7.29	6.44	30.18

3 Docking report of compound CDHPM-10e

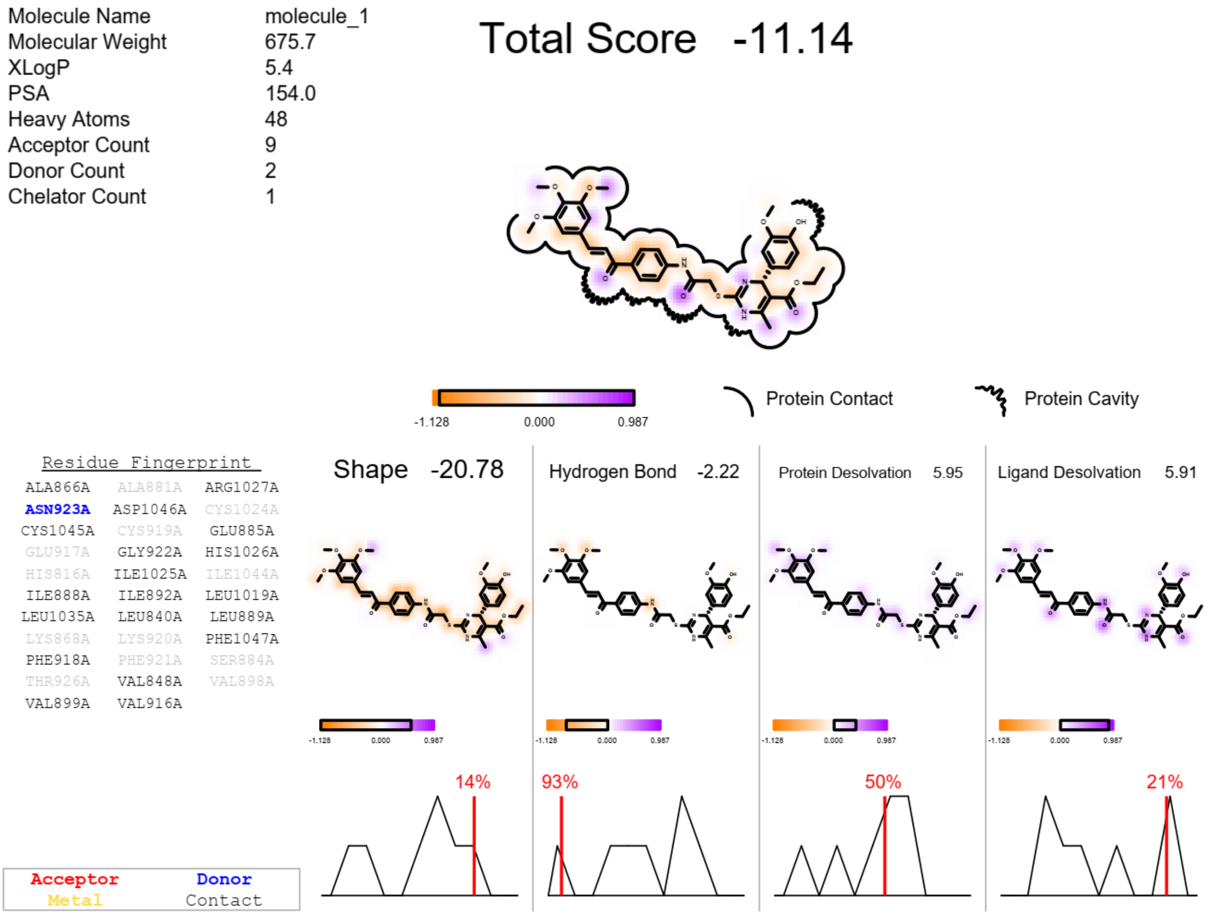

Figure S 49. Docking report of compound **CDHPM-10e**.

4 NCI-60 screening methodology

4.1 NCI 60 cell one-dose screen

General Description:

As of early 2007 all compounds submitted to the NCI 60 Cell screen are tested initially at a single high dose (10^{-5} M) in the full NCI 60 cell panel. Only compounds which satisfy pre-determined threshold inhibition criteria in a minimum number of cell lines will progress to the full 5-dose assay. The threshold inhibition criteria for progression to the 5-dose screen was selected to efficiently capture compounds with anti-proliferative activity based on careful analysis of historical DTP screening data. The threshold criteria may be updated as additional data becomes available [1, 2].

4.2 NCI 60 cell five-dose screen

Compounds which exhibit significant growth inhibition in the One-Dose Screen are evaluated against the 60-cell panel at five concentration levels [1, 2].

The human tumor cell lines of the cancer screening panel are grown in RPMI 1640 medium containing 5% fetal bovine serum and 2 mM L-glutamine. For a typical screening experiment, cells are inoculated into 96 well microtiter plates in 100 µL at plating densities ranging from 5,000 to 40,000 cells/well depending on the doubling time of individual cell lines. After cell inoculation, the microtiter plates are incubated at 37° C, 5 % CO_2, 95 % air and 100 % relative humidity for 24 h prior to addition of experimental drugs.

After 24 h, two plates of each cell line are fixed *in situ* with TCA, to represent a measurement of the cell population for each cell line at the time of drug addition (Tz). Experimental drugs are solubilized in dimethyl sulfoxide at 400-fold the desired final maximum test concentration and stored frozen prior to use. At the time of drug addition, an aliquot of frozen concentrate is thawed and diluted to twice the desired final maximum test concentration with complete medium containing 50 µg/ml gentamicin. Additional four, 10-fold or ½ log serial dilutions are made to provide a total of five drug concentrations plus control. Aliquots of 100 µl of these different drug dilutions are added to the appropriate microtiter wells already containing 100 µl of medium, resulting in the required final drug concentrations.

Following drug addition, the plates are incubated for an additional 48 h at 37°C, 5 % CO_2, 95 % air, and 100 % relative humidity. For adherent cells, the assay is terminated by the addition of cold TCA. Cells are fixed *in situ* by the gentle addition of 50 µl of cold 50 % (w/v) TCA (final concentration, 10 % TCA) and incubated for 60 minutes at 4°C. The supernatant is discarded, and the plates are washed five times with tap water and air dried. Sulforhodamine B (SRB) solution (100 µl) at 0.4 % (w/v) in 1 % acetic acid is added to each well, and plates are incubated for 10 minutes at room temperature. After staining, unbound dye is removed by washing five times with 1 % acetic acid and the plates are air dried. Bound stain is subsequently solubilized with 10 mM trizma base, and the absorbance is read on an automated plate reader at a wavelength of 515 nm. For suspension cells, the methodology is the same except that the assay is terminated by fixing settled cells at the bottom of the wells by gently adding 50 µl of 80 % TCA (final concentration, 16 % TCA). Using the seven absorbance measurements [time zero, (Tz), control growth, (C), and test growth in the presence of drug at the five concentration levels (Ti)], the percentage growth is calculated at each of the drug concentrations levels. Percentage growth is calculated as:

[(Ti-Tz)/(C-Tz)] x 100 for concentrations for which Ti>/=Tz

[(Ti-Tz)/Tz] x 100 for concentrations for which Ti<Tz.

Three dose response parameters are calculated for each experimental agent. Growth inhibition of 50 % (GI_{50}) is calculated from [(Ti-Tz)/(C-Tz)] x 100 = 50, which is the drug concentration resulting in a 50% reduction in the net protein increase (as measured by SRB staining) in control cells during the drug incubation. The drug concentration resulting in total growth inhibition (TGI) is calculated from Ti = Tz. The LC_{50} (concentration of drug resulting in a 50% reduction in the measured protein at the end of the drug treatment as compared to that at the beginning) indicating a net loss of cells following treatment is calculated from [(Ti-Tz)/Tz] x 100 = -50. Values are calculated for each of these three parameters if the level of activity is reached; however, if the effect is not reached or is exceeded, the value for that parameter is expressed as greater or less than the maximum or minimum concentration tested.

5 References

1. Elbadawi MM, Eldehna WM, Wang W, Agama KK, Pommier Y, Abe M. Discovery of 4-alkoxy-2-aryl-6,7-dimethoxyquinolines as a new class of topoisomerase I inhibitors endowed with potent in vitro anticancer activity. European Journal of Medicinal Chemistry 2021;215:113261-113281.
2. NCI. NCI-60 Screening Methodology 2021 [https://dtp.cancer.gov/discovery_development/nci-60/methodology.htm.
